# Computational modelling of wet adhesive mussel foot proteins (Bivalvia): Insights into the evolutionary convolution in diverse perspectives

**DOI:** 10.1038/s41598-020-59169-y

**Published:** 2020-02-13

**Authors:** P. P. Anand, Y. Shibu Vardhanan

**Affiliations:** Biochemistry & Toxicology Division, Department of Zoology, University of Calicut, Kerala, 673 635 India

**Keywords:** Protein sequence analyses, Protein structure predictions

## Abstract

Underwater adhesion in mussels (Bivalvia) is an extreme adaptation to achieve robust and firm wet adhesion in the freshwater/brackish/ocean, which biochemically shaped through millions of years. The protein-based adhesion has huge prospective in various fields like industry, medical, etc. Currently, no comprehensive records related to the systematic documentation of structural and functional properties of Mussel foot proteins (Mfps). In this study, we identified the nine species of bivalves in which the complete sequence of at least one adhesive protein is known. The insilico characterization revealed the specific physio-chemical structural and functional characters of each Mfps. The evolutionary analyses of selected bivalves are mainly based on Mfps, Mitogenome, and TimeTree. The outcome of the works has great applications for designing biomimetic materials in future.

## Introduction

Some groups of mussels are capable to produce proteinaceous glue- like sticky material known as byssus thread made by an array of foot proteins (fps). This byssus contains mainly four parts i.e. Plaque, thread, stem, and root. Individual threads proximally merged together to form stem and base of the stem (root) deeply anchored at the base of animal foot. Each byssus threads terminating distally with a flattened plaque which mediates adhesion to the substratum^1–4^. Each part of the byssus thread complex formed by the auto-assembly of secretory products originating from four distinct glands enclosed in the mussel foot^4,5^. These mussel foot protein (Mfps), mastered the ability to binding the diverse substratum by using adhesive plaques. 3,4- Dihydroxy phenylalanine (DOPA), is the core constituents in the Mfps, is formed by the post-translational hydroxylation of tyrosine. During the post- translational modification, polyphenol oxidases catalysis the o-hydroxylation of monophenols (tyrosine) to o-diphenols (DOPA), and the adhesion ability of Mfps are strongly correlated with the amount of DOPA^2,5–7^.

The byssal threads are engineered to withstand elevated mechanical loads applied by waves and currents in subtidal and intertidal zones.^4,8^. In recent decades, there has been significant understanding of Bivalvia origin, diversities and Mfps. The magnificent moisture-resistant adhesive property of Mfps has inspired to the development of a wide variety of functional materials^2,4,9^. Designing of mussel-mimetic adhesive materials, initially we need to understand the specific physio-chemical and functional property of each Mfps. This works aims to divulge the physio-chemical structural and functional characterization of currently available all Mfps of various species. And also disclose the evolutionary diversification and molecular clock level speciation of byssus thread producing bivalves and Mfps. The structural modeling and functional analysis of Mfps helps to understanding the which Mfps is highly promising for specific industrial and therapeutical applications.

## Results and Discussion

### Distribution frequency of available Mfps

A total of 78 Mfps are available in NCBI protein bank. Among these, 34 Mfps in *Mytilus californianus* Conrad,1837 (Mytilida: Mytilidae), 26 in *Mytilus unguiculatus* Valenciennes,1858 (Mytilida: Mytilidae) (Synonym of *M.coruscus*), five in *Perna viridis* (Linnaeus,1758) (Mytilida: Mytilidae), four in *Perna canaliculus* (Gmelin,1791) (Mytilida: Mytilidae), three in *Mytilus galloprovincialis* Lamarck,1819 (Mytilida: Mytilidae), two in *Mytilus edulis* Linnaeus,1758 (Mytilida: Mytilidae) and *Mizuhopecten yessoensis* (Jay,1857) (Pectinida: Pectinidae) and one in *Atrina pectinata* (Linnaeus,1767) (Pteriida: Pinnindae) and *Dreissena polymorpha* (Pallas,1771) (Myida: Dreissenidae). Scientific names of selected Bivalvia species were validated in Catalogue of Life:2019 Annual Checklist (http://www.catalogueoflife.org/annual- checklist/2019) and in World Register of Marine Species (WORMS) (http://www.marinespecies. org/index.php).

### Molecular modeling of Mfps

**Figure 1:**
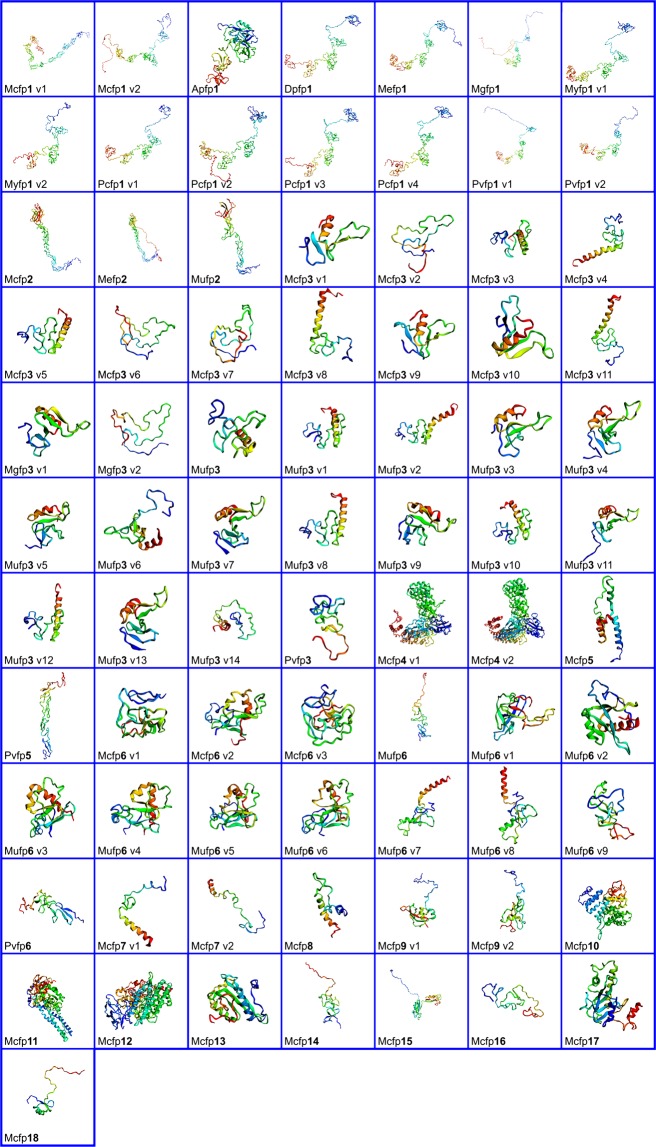
Ribbon diagram of the three-dimensional structure of mussel foot proteins (Mfps), visualized in EzMol 2.1.

Structural information of Mfps is not available in PDB (Protein Data Bank). The complete structure of each Mfps is mandatory for analyzing their structural and functional aspects. Comparative homology modeling of Mfps was done by using the MUSTER server^10^. The best model template was selected to develop a full protein model (Table 1: Template used for each Mfps modelling). All protein models are visualized using PyMol tool and EzMol 2.1. (Fig.1 and Supplementary Data S2- Table 2).

**Table 1:** List of Mfps used for *In silico* analysis with results of Ramachandran Plot analysis (Generated in PDBsum and PROCHECK).

Sl. No	Bivalve	GenBank Accession No.	Foot protein (fps)	Variant (v)	AA residues	Template	Ramachandran plot		
							Core region %	Additional allowed region %	Total %
1	*Mytilus californianus*	AAY29131.1	Fp1	Mcfp v1	763	3gavA	67.4	23.7	91.1
2		AAY29132.1		Mcfp v2	672	2nbiA	65.0	24.0	89.0
3		AST36139.1	FP2	Mcfp2	431	4xbmB	76.5	17.7	94.2
4		AAY29124.1	FP3	Mcfp3 v1	66	4ntqA	83.7	12.2	95.9
5		AAY29125.1		Mcfp3 v2	68	5w3nA	82.4	15.7	98.1
6		AAY29126.1		Mcfp3 v3	78	5xn14	82.5	15.8	98.3
7		AAY29127.1		Mcfp3 v4	78	5xn14	81.0	15.5	96.5
8		AAY29128.1		Mcfp3 v5	78	5xn14	81.0	15.5	96.5
9		AAY29129.1		Mcfp3 v6	78	5w3nA	77.6	17.2	94.8
10		AAY29130.1		Mcfp3 v7	75	5w3nA	83.6	12.7	96.3
11		AAZ94726.1		Mcfp3 v8	69	6igz7	80.4	15.7	96.1
12		AAZ94727.1		Mcfp3 v9	69	4ntqA	72.0	20.0	92.0
13		AAZ94728.1		Mcfp3 v10	69	4ntqA	74.0	18.0	92.0
14		AAZ94729		Mcfp3 v11	69	6igz7	86.0	10.0	96.0
15		ABC84184.1	Fp4	Mcfp4 v1	770	5a1uE	83.7	13.3	97.0
16		ABC84185.1		Mcfp4 v2	810	5a1uE	82.0	13.5	95.5
17		ABE01084.1	Fp5	Mcfp5	96	5yfpE1	74.7	18.7	93.4
18		ABC84186.1	Fp6	Mcfp6 v1	121	5ubmI	73.0	23.0	96.0
19		ABC84187.1		Mcfp6 v2	121	6ntwA	82.2	15.8	98.0
20		ABC84188.1		Mcfp6 v3	121	5ubmI	80.2	15.8	96.0
21		AST36124.1	Fp7	Mcfp7 v1	59	6ijoH	91.3	8.7	100.0
22		AST36126.1		Mcfp7 v2	65	2rmsB	89.4	8.5	97.9
23		AST36125.1	Fp8	Mcfp8	68	3dl8E	87.2	10.6	97.8
24		AST36127.1	Fp9	Mcfp9 v1	128	2kfwA2	78.4	15.5	93.9
25		AST36129.1		Mcfp9 v2	125	2kfwA2	75.0	18.8	93.8
26		AST36128.1	Fp10	Mcfp10	319	4eqfA	83.6	12.9	96.5
27		AST36130.1	Fp11	Mcfp11	496	6fgzA	87.8	8.3	96.1
28		AST36131.1	Fp12	Mcfp12	694	6hzeA	79.8	15.6	95.4
29		AST36132.1	Fp13	Mcfp13	158	2vezA	86.0	11.0	97.0
30		AST36133.1	Fp14	Mcfp14	120	2kraA	83.0	14.0	97.0
31		AST36134.1	Fp15	Mcfp15	249	2zx0A	86.6	10.2	96.8
32		AST36135.1	Fp16	Mcfp16	94	1n7dA	62.5	28.8	91.3
33		AST36136.1	Fp17	Mcfp17	203	4ixjA	81.4	14.1	95.5
34		AST36137.1	Fp18	Mcfp18	72	1c55A	89.1	7.8	96.9
35	*Atrina pectinata*	AIW04139.1	Fp1	Apfp1	352	5xtsA	78.1	18.6	96.7
36	*Dreissena polymorpha*	AAF75279.1	Fp1	Dpfp1	430	2nbiA	58.3	27.8	86.1
37	*Mytilus edulis*	AAX23968.1	Fp1	Mefp1	565	2nbiA	64.3	23.5	87.8
38		AAX23970.1	Fp2	Mefp2	504	4xbmB	82.9	14.5	97.4
39	*Mytilus galloprovincialis*	BAA09851.1	Fp1	Mgfp1	751	2nbiA	70.3	18.6	88.9
40		BAB16314.1	Fp3	Mgfp3 v1	70	4ntqA	90.4	7.7	98.1
41		BAB16315.1		Mgfp3 v2	77	5w3nA	84.5	13.8	98.3
42	*Mytilus unguiculatus*	ALA16015.1	Fp2	Mufp2	284	4xbmB	79.1	17.7	96.8
43		ACT66140.1	Fp3	Mufp3	77	5xnl4	75.0	19.6	94.6
44		ADB79738.1		Mufp3 v1	78	5xnl4	82.5	14.0	96.5
45		ADB79739.1		Mufp3 v2	78	5xnl4	82.5	14.0	96.5
46		ADB79740.1		Mufp3 v3	66	4ntqA	86.3	11.8	98.1
47		ADB79741.1		Mufp3 v4	78	4ntqA	82.1	12.5	94.6
48		ADB79742.1		Mufp3 v5	78	4ntqA	77.2	17.5	94.7
49		ADB79743.1		Mufp3 v6	77	3ck9B	84.2	14.0	98.2
50		ADB79744.1		Mufp3 v7	78	4ntqA	77.2	17.5	94.7
51		ADB79745.1		Mufp3 v8	78	5xnl4	81.1	13.2	94.3
52		ADB79746.1		Mufp3 v9	78	4ntqA	77.2	14.0	91.2
53		ADB79747.1		Mufp3 v10	78	5xnl4	85.7	10.7	96.4
54		ADB79748.1		Mufp3 v11	80	4ntqA	74.6	20.3	94.9
55		ADB79749.1		Mufp3 v12	78	5xnl4	82.5	12.3	94.8
56	*Mytilus unguiculatus*	ADB79750.1		Mufp3 v13	80	4ntqA	75.0	17.9	92.9
57		ADB79751.1		Mufp3 v14	72	5z62G	80.8	15.4	96.2
58		ALA16019.1	Fp6	Mufp6	122	2ahxB3	82.4	13.7	96.1
59		ADB79752.1		Mufp6 v1	123	6jyxA	74.5	19.6	94.1
60		ADB79753.1		Mufp6 v2	123	5uvdA1	87.1	12.9	100.0
61		ADB79754.1		Mufp6 v3	123	6ntwA2	89.2	9.8	99.0
62		ADB79755.1		Mufp6 v4	123	6ntwA2	87.4	11.7	99.1
63		ADB79756.1		Mufp6 v5	123	6ntwA2	81.4	11.8	93.2
64		ADB79757.1		Mufp6 v6	123	6ntwA2	81.4	15.7	97.1
65		ADB79758.1		Mufp6 v7	102	5x0mA	77.4	20.2	97.6
66		ADB79759.1		Mufp6 v8	97	5x0mA2	74.7	20.3	95.0
67		ADB79760.1		Mufp6 v9	96	1yy9A3	68.4	25.3	93.7
68	*Mizuhopecten yessoensis*	OWF35062.1	Fp1	Myfp1 v1	505	2nbiA	60.2	27.0	87.2
69		OWF42107.1		Myfp1 v2	324	2nbiA	65.5	25.0	90.5
70	*Perna canaliculus*	AAY29133.1	Fp1	Pcfp1 v1	436	2nbiA	57.6	29.7	87.3
71		AAY29134.1		Pcfp1 v2	432	2nbiA1	60.2	28.1	88.3
72		AAY29135		Pcfp1 v3	404	2nbiA	59.0	25.2	84.2
73		AAY29136.1		Pcfp1 v4	388	2nbiA	58.4	29.4	87.8
74	*Perna viridis*	AAY46226.1	Fp1	Pvfp1 v1	561	2nbiA	66.0	22.4	88.4
75		AAY46227.1		Pvfp1 v2	431	2nbiA1	61.7	25.3	87.0
76		AGZ84285.1	Fp3	Pvfp3	70	3ds1a2	77.4	17.7	95.1
77		AGZ84279.1	Fp5	Pvfp5	176	5uk5A	84.3	15.7	100.0
78		AGZ84283.1	Fp6	Pvfp6	122	2mhpA	75.8	20.0	95.8

### Validation of Mfps model

**Figure 2:**
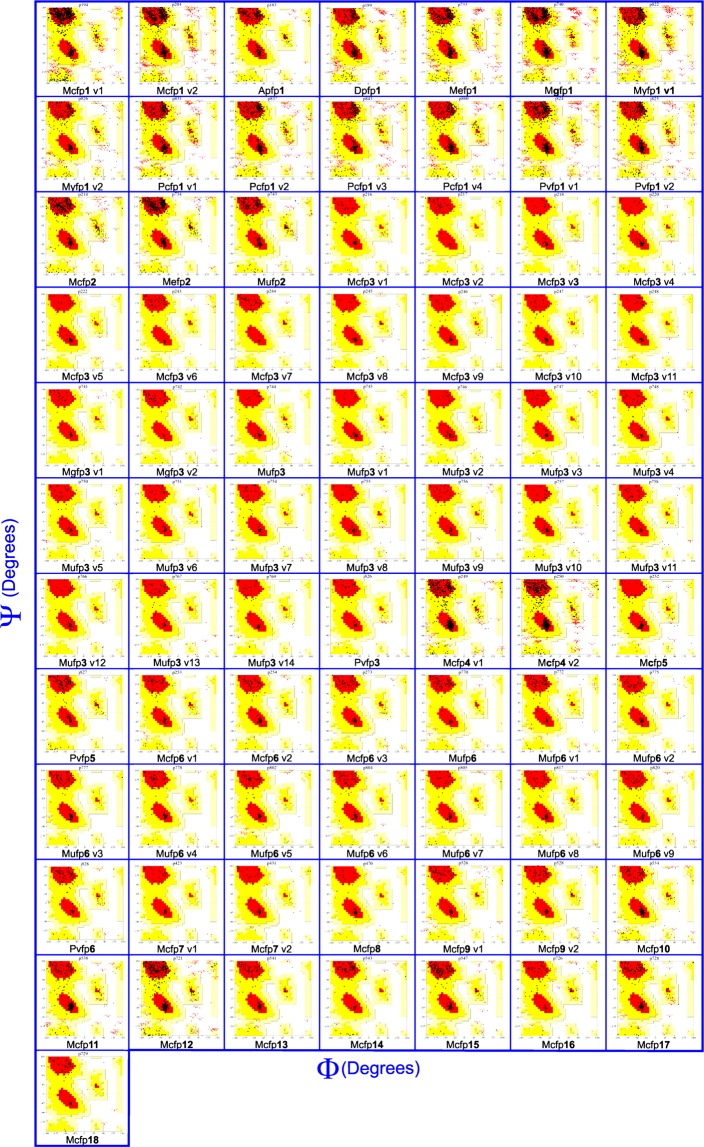
Ramachandran plot of mussel foot proteins (Mfps), generated in PROCHECK, PDBSum.

In a good protein model, is expected that there should be more than 90% of the residue in the core or favored region and additional allowed regions^11^. By analyzing the Ramachandran plot, among the 78 Mfps models, 67 protein models are highly stable because of the 90% residue occurred in core and additional allowed regions of Ramachandran plot. The other 11 protein models are moderately stable because the 85–90 % of residue occurred in core and additional allowed regions of Ramachandran plot (Table 1 and Fig. 2).

### Promotif documentation of Mfps

The simulated Mfps models were further analyzed using PDBsum for the promotif documentation. In PDBsum server^12^, analyze the secondary structure characterization of Mfps likes, sheets, beta-alpha beta units, beta hairpins, Psi loops, strands, helices, helix-helix interactions, beta turns, gamma turns and disulfides. The disulfide bond only present in Mcfp2, Mcfp6 v1, Mcfp6 v3, Mcfp14, Mcfp15, Mcfp16, Mcfp18, Apfp1, Mefp2, Mufp2, Mufp6 v9, Pvfp3, Pvfp5 and Pvfp6. The disulfide bond can be formed under oxidizing conditions and play an important role in the folding and stability of the extracellular proteins^5,13–15^. Normally the disulfide bonds are the crosslinking groups that contribute strength of the protein. All Mfps contained beta turns in varying numbers, but the psi loops present in Mcfp11 only. By analyzing the promotif of Mfps indicated as most of the Mfps exhibited the moderate structural complexity because of the limited number of secondary structural modifications (Table 2).

**Table 2:** Promotif documentation of all Mfps, generated in PDBsum server.

Sl. No	Bivalve	Variant	Sheets	Beta alpha beta unit	Beta hairpins	Psi loops	Beta bulge	Strand	helices	Helix-helix interactions	Beta turns	Gamma turns	Disulphides
1	*Mytilus californianus*	Mcfp1 v1	7	0	6	0	1	15	2	0	97	11	0
2		Mcfp1 v2	0	0	0	0	0	0	6	0	120	19	0
3		Mcfp2	12	0	11		3	27	2	0	71	5	17
4		Mcfp3 v1	2	0	2	0	1	4	1	0	12	1	0
5		Mcfp3 v2	0	0	0	0	0	0	0	0	8	0	0
6		Mcfp3 v3	0	0	0	0	0	0	2	0	20	1	0
7		Mcfp3 v4	0	0	0	0	0	0	2	0	21	0	0
8		Mcfp3 v5	0	0	0	0	0	0	2	0	17	0	0
9		Mcfp3 v6	0	0	0	0	0	0	0	0	10	0	0
10		Mcfp3 v7	0	0	0	0	0	0	0	0	11	1	0
11		Mcfp3 v8	0	0	0	0	0	0	1	0	13	2	0
12		Mcfp3 v9	2	0	2	0	0	4	1	0	10	2	0
13		Mcfp3 v10	2	0	2	0	0	4	1	0	9	2	0
14		Mcfp3 v11	0	0	0	0	0	0	1	0	11	1	0
15		Mcfp4 v1	3	0	2	0	0	7	38	54	74	22	0
16		Mcfp4 v2	5	0	3	0	4	12	41	78	79	19	0
17		Mcfp5	0	0	0	0	0	0	5	4	19	1	0
18		Mcfp6 v1	2	0	2	0	1	4	1	0	26	1	2
19		Mcfp6 v2	2	1	2	0	1	6	4	2	20	3	0
20		Mcfp6 v3	2	0	2	0	1	4	3	0	27	2	2
21		Mcfp7 v1	0	0	0	0	0	0	1	0	11	2	0
22		Mcfp7 v2	0	0	0	0	0	0	1	0	12	1	0
23		Mcfp8	0	0	0	0	0	0	4	1	9	1	0
24		Mcfp9 v1	1	0	1	0	1	3	2	0	17	2	0
25		Mcfp9 v2	0	0	0	0	0	0	1	0	21	1	0
26		Mcfp10	0	0	0	0	0	0	17	32	40	2	0
27		Mcfp11	2	2	1	2	1	8	22	33	33	5	0
28		Mcfp12	5	5	0	0	2	13	25	21	78	13	0
29		Mcfp13	1	1	2	0	2	4	6	2	25	4	0
30		Mcfp14	1	0	1	0	1	3	1	0	13	3	5
31		Mcfp15	3	0	1	0	3	7	6	1	25	5	2
32		Mcfp16	0	0	0	0	0	0	0	0	15	4	2
33		Mcfp17	2	0	5	0	2	7	7	2	28	5	0
34		Mcfp18	1	0	1	0	0	2	1	0	3	5	3
35	*Atrina pectinate*	Apfp1	2	0	4	0	3	10	6	0	39	9	3
36	*Dreissena polymorpha*	Dpfp1	0	0	0	0	0	0	2	0	105	12	0
37	*Mytilus edulis*	Mefp1	0	0	0	0	0	0	9	0	113	11	0
38		Mefp2	10	0	10	0	4	23	2	0	74	7	17
39	*Mytilus galloprovincialis*	Mgfp1	0	0	0	0	0	0	8	0	127	9	0
40		Mgfp3 v1	1	0	3	0	1	4	2	0	11	1	0
41		Mgfp3 v2	0	0	0	0	0	0	0	0	10	1	0
42	*Mytilus unguiculatus*	Mufp2	8	0	8	0	2	16	1	0	46	1	12
43		Mufp3	0	0	0	0	0	0	2	0	22	1	0
44		Mufp3 v1	0	0	0	0	0	0	3	1	17	1	0
45		Mufp3 v2	0	0	0	0	0	0	3	2	19	1	0
46		Mufp3 v3	2	0	2	0	1	4	1	0	13	1	0
47		Mufp3 v4	1	0	1	0	0	2	2	0	12	2	0
48		Mufp3 v5	1	0	1	0	0	2	1	0	11	3	0
49		Mufp3 v6	1	0	1	0	0	2	3	0	11	1	0
50		Mufp3 v7	2	0	2	0	2	6	1	0	10	0	0
51		Mufp3 v8	0	0	0	0	0	0	2	1	24	0	0
52		Mufp3 v9	2	0	4	0	2	6	1	0	14	2	0
53		Mufp3 v10	0	0	0	0	0	0	3	1	19	1	0
54		Mufp3 v11	2	0	3	0	0	5	1	0	13	1	0
55		Mufp3 v12	0	0	0	0	0	0	1	0	22	0	0
56	*Mytilus unguiculatus*	Mufp3 v13	3	0	3	0	1	6	1	0	14	1	0
57		Mufp3 v14	0	0	0	0	0	0	1	0	21	4	0
58		Mufp6	3	0	3	0	0	6	0	0	24	2	0
59		Mufp6 v1	4	0	3	0	1	9	0	0	14	4	0
60		Mufp6 v2	1	0	3	0	1	5	3	0	19	5	0
61		Mufp6 v3	2	1	2	0	0	5	3	1	18	3	0
62		Mufp6 v4	2	1	2	0	0	5	4	2	17	1	0
63		Mufp6 v5	2	1	2	0	0	5	4	1	16	2	0
64		Mufp6 v6	1	0	1	0	0	2	2	0	20	5	0
65		Mufp6 v7	1	0	1	0	0	2	3	0	12	2	0
66		Mufp6 v8	1	0	1	0	0	2	4	0	10	4	0
67		Mufp6 v9	2	0	2	0	2	4	1	0	23	4	1
68	*Mizuhopecten yessoensis*	Myfp1 v1	0	0	0	0	0	0	8	1	111	20	0
69		Myfp1 v2	0	0	0	0	0	0	3	0	78	11	0
70	*Perna canaliculus*	Pcfp1 v1	0	0	0	0	0	0	5	0	103	9	0
71		Pcfp1 v2	0	0	0	0	0	0	6	0	100	11	0
72		Pcfp1 v3	0	0	0	0	0	0	4	0	96	21	0
73		Pcfp1 v4	0	0	0	0	0	0	6	0	85	6	0
74	*Perna viridis*	Pvfp1 v1	0	0	0	0	0	0	7	0	120	16	0
75		Pvfp1 v2	0	0	0	0	0	0	5	0	102	12	0
76		Pvfp3	0	0	0	0	0	0	2	0	15	3	2
77		Pvfp5	5	0	5	0	2	10	2	0	27	0	11
78		Pvfp6	4	0	5	0	2	9	1	0	20	3	5

### Signal peptide predictions of Mfps

To verify the signal peptide in Mfps, Phobius and SignaIP 5.0 server were used. *Mizuhopecten yessoensis* foot protein, Myfp1 V1 and V2 don’t contain any signal peptide region. Except Myfp, all other proteins have the signal peptide regions (~1–20 amino acid sequences), the efficiency of protein secretion in extracellular region is highly determined by the signal peptide and also the signal peptide are extremely heterogenous in nature^16^ (Table 3).

**Table 3:** Physio-chemical characterization of Mfps (Generated in Expasy protparam) with signal peptide prediction (Generated in Phobius and SignaIP 5.0).

Sl. No	Bivalve	Variant	Signal Peptide Prediction		pI	Mw (Dalton)	II	AI	EC	GRAVY	Half-life (in Hours) – *in vivo*	
			SignaIP 5.0	Phobius							Yeast	*E. coli*
1	*Mytilus californianus*	Mcfp1 v1	1–24	1–20	10.04	85024.22	41.20	26.57	204255	−1.357	20	10
2		Mcfp1 v2	1–24	1–20	10.04	78048.08	40.93	28.07	186375	−1.329	20	10
3		Mcfp2	1–27	1–17	9.04	46753.52	14.42	27.80	58545	−0.890	20	10
4		Mcfp3 v1	1–24	1–22	10.09	7548.70	25.90	61.97	31400	−0.658	20	10
5		Mcfp3 v2	1–26	1–19	10.05	7791.92	23.61	61.62	36900	−0.629	20	10
6		Mcfp3 v3	1–24	1–22	8.80	8836.73	13.29	55.00	42860	−0.505	20	10
7		Mcfp3 v4	1–24	1–22	9.07	8973.87	19.12	48.72	44350	−0.649	20	10
8		Mcfp3 v5	1–24	1–22	9.07	9000.90	13.12	48.72	44350	−0.683	20	10
9		Mcfp3 v6	1–24	1–22	9.70	9120.28	32.33	48.72	46870	−0.791	20	10
10		Mcfp3 v7	1–24	1–22	7.88	8623.45	11.60	50.67	42860	−0.631	20	10
11		Mcfp3 v8	1–24	1–22	8.86	7920.73	12.00	55.07	39880	−0.626	20	10
12		Mcfp3 v9	1–24	1–22	7.94	7799.59	8.98	60.72	38390	-0.501	20	10
13		Mcfp3 v10	1–24	1–22	7.94	7786.59	8.17	60.72	38390	−0.461	20	10
14		Mcfp3 v11	1–24	1–22	7.94	7790.59	12.19	56.52	38390	−0.439	20	10
15		Mcfp4 v1	1–26	1–19	10.20	90254.35	43.14	87.04	30955	−0.835	20	10
16		Mcfp4 v2	1–26	1–19	10.48	95385.30	42.38	87.65	30955	−0.853	20	10
17		Mcfp5	1–25	1–18	9.77	10928.51	20.17	47.71	29925	−0.847	20	10
18		Mcfp6 v1	1–24	1–22	8.85	13928.70	45.50	41.07	33530	−0.550	20	10
19		Mcfp6 v2	1–25	1–22	9.24	13911.86	57.41	42.73	30550	−0.544	20	10
20		Mcfp6 v3	1–24	1–22	9.24	13824.73	55.42	42.73	30550	−0.551	20	10
21		Mcfp7 v1	1–30	1–24	10.41	6333.36	23.99	71.02	8940	−0.163	20	10
22		Mcfp7 v2	1–30	1–25	10.17	6688.79	7.99	62.92	8940	−0.245	20	10
23		Mcfp8	1–26	1–26	10.00	7281.66	−7.47	70.58	16390	−0.122	20	10
24		Mcfp9 v1	1–21	1–19	9.74	13430.87	22.53	69.84	17420	−0.330	20	10
25		Mcfp9 v2	1–22	1–19	9.74	13312.69	29.75	66.08	17420	−0.466	20	10
26		Mcfp10	1–24	1–16	8.59	6316.67	29.41	68.68	82795	−0.285	20	10
27		Mcfp11	1–30	1–20	9.89	58635.75	36.53	55.46	108585	−0.914	20	10
28		Mcfp12	1–25	1–21	9.92	82004.53	41.55	55.55	144845	−0.831	20	10
29		Mcfp13	1–30	1–22	10.35	15181.82	44.67	72.20	35425	−0.482	20	10
30		Mcfp14	1–23	1–23	8.65	13006.00	41.16	75.50	10720	−0.071	20	10
31		Mcfp15	1–21	1–18	9.52	2876.43	42.30	50.12	47635	−0.794	20	10
32		Mcfp16	1–25	1–19	9.33	10630.48	33.36	50.74	3730	−0.666	20	10
33		Mcfp17	1–27	1–27	9.21	23576.23	36.72	65.22	70205	−0.446	20	10
34		Mcfp18	1–22	1–20	8.81	7925.25	47.24	63.75	9565	−0.026	20	10
35	*Atrina pectinate*	Apfp1	1–25	1–18	9.47	40382.27	33.74	57.76	121185	−0.662	20	10
36	*Dreissena polymorpha*	Dpfp1	1–30	1–19	5.24	49361.70	53.65	31.40	122860	−1.331	20	10
37	*Mytilus edulis*	Mefp1	1–30	1–20	9.99	6467.22	36.51	26.02	152105	−1.280	20	10
38		Mefp2	1–23	1–17	9.14	54459.22	18.05	26.47	59630	−0.896	20	10
39	*Mytilus galloprovincialis*	Mgfp1	1–34	1–34	9.99	85791.18	44.31	20.80	213195	−1.353	20	10
40		Mgfp3 v1	1–25	1–24	10.24	8003.02	48.41	55.71	31400	−0.801	20	10
41		Mgfp3 v2	1–25	1–24	10.32	843.79	54.86	49.35	38390	−0.908	20	10
42	*Mytilus unguiculatus*	Mufp2	1–26	1–17	9.23	31206.12	16.16	34.30	42145	−0.842	20	10
43		Mufp3	1–27	1–27	9.13	8769.96	6.05	63.72	42860	−0.350	20	10
44		Mufp3 v1	1–26	1–19	9.13	8715.64	15.14	66.28	38850	−0.426	20	10
45		Mufp3 v2	1–25	1–19	8.64	8701.63	13.24	66.28	44350	−0.383	20	10
46		Mufp3 v3	1–25	1–19	10.33	7696.70	42.06	60.61	29910	−0.889	20	10
47		Mufp3 v4	1–25	1–19	8.80	8729.62	18.18	61.28	44350	−0.449	20	10
48		Mufp3 v5	1–25	1–19	8.66	8685.63	14.33	66.28	42860	−0.331	20	10
49		Mufp3 v6	1–25	1–19	7.90	8689.55	22.48	67.14	38850	−0.451	20	10
50		Mufp3 v7	1–25	1–19	7.93	8699.60	15.74	67.56	41370	−0.428	20	10
51		Mufp3 v8	1–27	1–22	9.19	8612.64	15.36	60.00	32890	−0.406	20	10
52		Mufp3 v9	1–27	1–24	7.90	8453.68	15.74	70.00	42860	−0.373	20	10
53		Mufp3 v10	1–25	1–22	9.30	8609.75	17.49	66.28	36900	−0.110	20	10
54		Mufp3 v11	1–26	1–22	8.83	9034.05	20.9	64.62	41370	−0.310	20	10
55		Mufp3 v12	1–27	1–19	7.90	8725.63	15.74	67.56	42860	−0.404	20	10
56	*Mytilus unguiculatus*	Mufp3 v13	1–27	1–19	9.63	8625.82	14.03	64.62	19940	−0.184	20	10
57		Mufp3 v14	1–26	1–19	10.05	8196.27	25.11	54.17	38390	−0.772	20	10
58		Mufp6	1–22	1–22	9.05	14175.06	38.31	39.18	36510	−0.678	20	10
59		Mufp6 v1	1–25	1–25	9.13	14410.36	39.18	34.07	36510	−0.694	20	10
60		Mufp6 v2	1–25	1–22	9.13	14455.30	38.70	34.88	39490	−0.750	20	10
61		Mufp6 v3	1–25	1–22	9.07	14392.29	40.29	37.24	36510	−0.669	20	10
62		Mufp6 v4	1–25	1–25	9.18	14386.30	40.58	31.15	38000	−0.788	20	10
63		Mufp6 v5	1–25	1–25	9.12	14454.42	36.96	34.07	38000	−0.727	20	10
64		Mufp6 v6	1–25	1–25	9.07	14538.45	33.80	27.72	36510	−0.677	20	10
65		Mufp6 v7	1–25	1–25	9.16	11972.66	36.17	37.25	30300	−0.639	20	10
66		Mufp6 v8	1–21	1–17	9.08	11396.92	40.19	39.18	31790	−0.668	20	10
67		Mufp6 v9	1–24	1–16	8.88	11138.42	33.51	30.52	30300	−0.938	20	10
68	*Mizuhopecten yessoensis*	Myfp1 v1	ND	ND	5.12	56961.06	23.62	6.97	8480	−2.038	20	10
69		Myfp1 v2	ND	ND	8.66	33579.64	62.60	56.48	118370	−0.615	20	10
70	*Perna canaliculus*	Pcfp1 v1	1–20	1–16	9.92	51565.91	−10.03	65.96	131995	−0.637	20	10
71		Pcfp1 v2	1–20	1–16	9.92	51078.31	−9.97	65.90	130505	−0.637	20	10
72		Pcfp1 v3	1–20	1–16	9.97	48252.05	−10.61	66.83	127150	−0.673	20	10
73		Pcfp1 v4	1–20	1–16	9.97	46301.65	−10.38	66.60	121190	−0.674	20	10
74	*Perna viridis*	Pvfp1 v1	1–20	1–20	11.20	63247.45	11.20	32.57	510930	−0.800	20	10
75		Pvfp1 v2	1–28	1–20	11.15	48042.87	15.66	33.67	362430	−0.780	20	10
76		Pvfp3	1–23	1–23	5.93	7919.17	44.26	64.00	10595	0.260	20	10
77		Pvfp5	1–18	1–18	9.29	19695.09	24.06	33.75	39000	−0.514	20	10
78		Pvfp6	1–19	1–17	6.48	13262.41	39.62	57.46	12920	0.102	20	10

pI (Isoelectric point), Mw (Molecular weight), II (Instability index), AI (Aliphatic index), EC (Extinction coefficient) and GRAVY (Grand average of hydropathicity). ND: Not detected.

### Accessible surface area (ASA) of Mfps

ASA of each Mfps is extremely unique because the size of the fps varies from each species. In the wet adhesion, the hydrophobic nature is very important^5^, so that perspectives the percentage of side ASA hydrophobic analysis of all Mfps revealed. Among the all Mfps, Pvfp1 v1 is showed the highest percentage of side ASA hydrophobic and followed by Pvfp1 v2 (Table 4).

**Table 4:** Accessible surface area of Mfps generated in VADAR server. (ND: Not detected).

Sl. No	Bivalve	Variant	Total ASA (A^2^)	Exposed Nonpolar ASA (A^2^)	Exposed polar ASA (A^2^)	Exposed charged ASA (A^2^)	% side ASA Hydrophobic (A^2^)
1	*Mytilus californianus*	Mcfp1 v1	54354.3	38193.8	10977.8	5182.7	25.70
2		Mcfp1 v2	61703.6	43389.1	13480.7	4833.9	28.23
3		Mcfp2	28106.8	17011.2	6720.1	4375.5	20.77
4		Mcfp3 v1	5209.0	3426.9	1196.6	585.5	26.86
5		Mcfp3 v2	7940.2	5438.8	1815.6	685.7	40.12
6		Mcfp3 v3	6817.9	4748.2	1700.9	368.8	40.25
7		Mcfp3 v4	6791.7	4804.3	1673.4	314.0	38.67
8		Mcfp3 v5	6589.2	4634.3	1576.7	378.2	37.06
9		Mcfp3 v6	8915.2	6049.7	2197.4	668.1	37.85
10		Mcfp3 v7	8316.2	5645.2	2294.8	376.2	39.56
11		Mcfp3 v8	6735.7	4668.2	1719.3	348.2	38.24
12		Mcfp3 v9	5298.4	3552.6	1417.1	328.7	37.24
13		Mcfp3 v10	5102.5	3400.6	1369.4	332.6	37.02
14		Mcfp3 v11	6827.5	4750.1	1644.7	432.8	39.74
15		Mcfp4 v1	36397.0	20650.4	5193.8	10552.8	26.84
16		Mcfp4 v2	37308.0	21249.2	5329.3	10729.5	27.11
17		Mcfp5	7366.2	4984.2	1362.8	1019.2	18.65
18		Mcfp6 v1	7754.1	4656.3	2288.2	809.6	24.16
19		Mcfp6 v2	8738.1	5182.1	2323.2	1232.8	21.21
20		Mcfp6 v3	7795.0	4889.5	1971.3	934.2	28.39
21		Mcfp7 v1	6455.1	4372.2	1094.8	988.2	32.13
22		Mcfp7 v2	6558.7	4620.7	1111.1	826.8	34.34
23		Mcfp8	6711.5	4944.5	1093.0	674.0	32.52
24		Mcfp9 v1	10823.5	6564.7	1960.0	2298.8	21.70
25		Mcfp9 v2	10833.7	6494.7	2013.4	2325.6	25.15
26		Mcfp10	16718.7	10454.6	3681.4	2582.7	34.18
27		Mcfp11	26224.0	15844.6	4453.3	5926.1	21.63
28		Mcfp12	32927.4	19211.7	6023.4	7692.3	19.09
29		Mcfp13	9718.7	5925.5	1991.9	1801.3	24.99
30		Mcfp14	9945.3	6185.1	2242.1	1518.1	40.15
31		Mcfp15	18644.2	11144.4	4544.9	2954.9	22.35
32		Mcfp16	8871.5	5481.7	1610.8	1779.0	24.91
33		Mcfp17	12578.3	7834.7	2931.0	1812.7	29.82
34		Mcfp18	7341.0	4762.4	1714.4	864.2	31.82
35	*Atrina pectinate*	Apfp1	21754.0	15043.6	3808.2	2902.2	38.67
36	*Dreissena polymorpha*	Dpfp1	34184.7	22793.1	7307.0	4084.6	32.77
37	*Mytilus edulis*	Mefp1	47802.4	33622.1	10934.8	3245.6	27.44
38		Mefp2	37694.4	23355.4	9188.8	5150.1	24.66
39	*Mytilus galloprovincialis*	Mgfp1	ND	ND	ND	ND	ND
40		Mgfp3 v1	5218.0	3288.9	1121.9	807.2	26.06
41		Mgfp3 v2	8730.4	5357.9	2293.6	1078.9	35.98
42	*Mytilus unguiculatus*	Mufp2	22841.5	14506.4	4889.4	3445.7	25.23
43		Mufp3	6482.2	4683.3	1543.8	255.1	40.33
44		Mufp3 v1	6943.9	4860.6	1757.3	326.1	38.58
45		Mufp3 v2	6347.8	4474.4	1658.8	214.5	39.76
46		Mufp3 v3	5364.3	3081.3	1508.0	775.0	27.20
47		Mufp3 v4	5975.8	4043.0	1660.2	272.6	32.45
48		Mufp3 v5	5424.1	3661.8	1409.8	352.4	33.41
49		Mufp3 v6	6801.4	4421.9	1863.7	515.7	31.92
50		Mufp3 v7	5328.9	3451.3	1498.0	379.5	31.69
51		Mufp3 v8	6623.1	4863.7	1378.7	380.8	45.93
52		Mufp3 v9	5328.9	3451.3	1498.0	379.5	31.69
53		Mufp3 v10	6562.7	4872.7	1427.7	262.3	49.20
54		Mufp3 v11	6048.9	4037.5	1621.3	390.1	34.00
55		Mufp3 v12	6574.2	4638.2	1680.8	255.1	42.08
56	*Mytilus unguiculatus*	Mufp3 v13	5690.5	4228.7	1226.2	235.5	54.01
57		Mufp3 v14	6830.6	4613.0	1679.1	538.4	37.47
58		Mufp6	11131.4	6985.8	3254.7	890.9	25.68
59		Mufp6 v1	9075.1	5278.1	2618.8	1178.2	16.89
60		Mufp6 v2	8518.2	5014.0	2389.0	1115.3	17.64
61		Mufp6 v3	8743.5	5300.6	2387.7	1055.3	20.17
62		Mufp6 v4	8680.7	5090.1	2460.18	1129.8	16.06
63		Mufp6 v5	8951.9	5493.0	2307.8	1151.1	18.11
64		Mufp6 v6	9017.3	5705.2	2288.0	1024.1	20.63
65		Mufp6 v7	8437.5	5233.0	2275.4	929.2	27.41
66		Mufp6 v8	7897.2	5104.9	1955.0	837.3	26.36
67		Mufp6 v9	7606.6	4752.6	2074.6	779.5	19.32
68	*Mizuhopecten yessoensis*	Myfp1 v1	37068.2	19088.8	13688.4	4291.0	11.35
69		Myfp1 v2	27092.1	17700.9	6792.2	2599.0	37.77
70	*Perna canaliculus*	Pcfp1 v1	3554.1	25946.1	6726.4	2881.7	35.45
71		Pcfp1 v2	35115.6	25368.2	7105.6	2641.8	34.81
72		Pcfp1 v3	32759.4	24128.3	6088.8	2542.2	35.47
73		Pcfp1 v4	31497.6	22851.4	5771.4	2874.8	36.18
74	*Perna viridis*	Pvfp1 v1	47417.8	36101.7	7917.3	3398.8	59.97
75		Pvfp1 v2	35152.4	26011.5	6002.5	3138.4	57.11
76		Pvfp3	5458.0	3605.8	1299.7	552.5	47.02
77		Pvfp5	13685.6	9005.2	3104.4	1575.9	22.71
78		Pvfp6	9501.7	6390.9	2301.5	808.3	45.36

### Functional characterization of Mfps

Functional characterization of Mfps, FFPred 3 server analyze the protein in three different categories like biological process prediction, cellular component prediction and molecular function prediction with GO (Gene Ontology) term^17^. This is the first attempt to revealing the molecular function, cellular process and biological activity of Mfps. By analyzing the biological process of Mfps, most of the Mfps show the cell surface receptor signaling pathway (GO:0007166) and cellular component prediction of Mfps shows that all Mfps is in the extracellular region (GO:0005576). The functional characterization of each Mfps showed unique functions. The wet adhesion is the core feature of all known Mfps^5,9^, other than this property the protein showed some different features also. G-protein coupled receptor activity (GO:0004930), is the common molecular function of all Mfps. (Supplementary data S3-Tables 3, 4 and 5).

*Mytilus californianus* foot protein (Mcfp), the fp1 showed the growth factor activity (GO:0008083). The mcfp2, exhibit the nine molecular function, and this protein also showed the zinc ion binding activity (GO:0008270) and endopeptidase activity (GO:0004175). In Mcfp3, contained 11 variants, all protein variants showed the receptor activity (GO:0004872), G-protein coupled receptor activity (GO:0004930) and Peptidase inhibitor activity (GO:0030414). Except for Mcfp3 v1, all other variants of Mcfp3 showed the enzyme inhibitory activity (GO:0004857). Purine nucleoside binding (GO:0001883) and catalytic activity (GO:0003824) are showed by Mcfp4 v1 and v2 respectively. Mcfp6 v2, Mcfp11, Mcfp15, Mcfp16 and Mcfp18 exhibit the zinc ion binding (GO:0008270) activity and Mcfp9 showed the co-factor binding activity (GO:0048037).

*Atrina pectinata* foot protein (Apfp1) showed the growth factor (GO:0008083) and cytokine activity (GO:0005125). *Dreissena polymorpha* foot protein (Dpfp1) showed the growth factor and G- protein-coupled receptor activity. In *Mytilus edulis* foot protein (Mefp), Mefp2, exhibit the zinc ion binding (GO:0008270) activity. Mefp2 showed the highest number of molecular functional activities comparing to the Mefp1. In *Mytilus galloprovincialis* foot protein (Mgfp), all proteins and their variant revealed the G-protein coupled receptor binding activity and Mgfp3 v1 and v2 showed the peptidase inhibitory activity.

The *Mytilus unguiculatus* foot protein (Mufp), contains three types of protein and their variant. Mufp2 showed the transmembrane signaling receptor activity, endopeptidase activity, signal transducer activity, serine hydrolase activity, cytokine, and zinc ion binding activity. In Mufp3 and their variants exhibit the peptidase inhibitory activity and G-protein coupled receptor activity. All proteins and their variables in Mufp6 showed the cytokine activity. Mufp6 and their variant like v3 and v9 exhibit the zinc ion binding activity. Except for Mufp6, all variants showed the cytokine receptor binding activity.

*Mizuhopecten yessoensis* foot protein (Myfp), showed the DNA binding (GO:0003677), cytoskeleton protein binding (GO:0008092) and nucleic acid binding (GO:0003676) activities. The Myfp1 v2 showed the sequence-specific DNA binding transcription factor activity. The *Perna canaliculus* foot protein (Pcfp), the fp1 have four variants and all variants showed the poly (A) RNA binding (GO:0044822) except Pcfp1 v1. *Perna viridis* foot protein (Pvfp), each protein has unique molecular functions. The Pvfp1 v1 and Pvfp6 showed the G-protein coupled receptor binding activity. Glycosaminoglycan binding (GO:0005539) activity observed in Pvfp1 v2. The variants like fp3 and fp5 exhibit the zinc ion binding activity.

### Chemical structural evaluation of Mfps

Amino acid compositional analysis of Apfp1, the major amino acid composition is lysine (15.3%) and proline (15.1%). Most of the amino acid in neutral charge and with positive charge clusters from 121 to 146, (KKPPVYKPKKPVYKPKKRPAYKPKKK), mixed and negative charge clusters are absent in Apfp1. Core block tandem repeats like PPVD, KPPV and PDYKP repeated two times and YKPKK repeated three times. Dpfp1 showed the highest abundance of proline (22.3%) and tyrosine (14.9%), the charge cluster analysis revealed the absence of positive, negative and mixed charge clusters in Dpfp1. tandem repeated blocks, FTTK, PVYPT, PVYPY, PVYPP, PEYP repeated two times and a four-times repetition of PVYP are also observed.

Mcfp1 v1- 23.6 % of amino acid contributed by proline and followed by tyrosine (18.7%). And the absence of specific charge clusters like positive, negative and mixed. Interestingly the presence of 66 copy repetition of YK.K...YPP. the element from location 82–741. Comparing to the first variants of Mcfp1, the v2 was identified as 23.2% of proline and followed by lysine (20.1%) and tyrosine (18.6%). Same as the v1, v2 doesn′t contain any charge clusters. The KKSYPPAYK tandemly repeated four times and also 60 copies of YK.K...YPP. periodically present in-between location 82–681 of Mcfp1 v2. Mcfp2 contained the highest amount of cysteine (14.4%) and followed by glycine (13.5%). Absence of charge clusters and PCKN tandemly repeated five times. In Mcfp3 contained 11 variants, the highest abundance of glycine (18.2%) present in first variants (v1) and followed by tyrosine (15.2%). YPRG repeated two times and without any charge clusters. Glycine (17.6%), tyrosine (14.7%) is the abundant amino-acid present in v2, and this variant doesn′t contain any charge clusters. GWNK is the only tandem repeats present in v2, and it repeated by two times. The highest abundant amino acid in v3 is glycine (20.5%) and tyrosine (17.9%). This variant doesn′t contain any tandem repeated blocks and charge clusters. Tyrosine composition in each variant like v4, v5, v6, v7, v8, v9, v10 and v11 is 19.2, 19.2, 16.2, 18.7, 17.4, 15.9, 15.9, 15.9% respectively. And these variants don′t show any tandem repeated regions and charge clusters. Mcfp4 contained two variants, tyrosine content same in both variants (2.2%). Histidine is the most abundant amino acid in both variants, v1 (23.0%) and v2 (23.8%). Negative charge cluster region in v1, sequence from 486–536 and 565–628 (DLSNDLHPDNNIEQIANDHVNDIAQSTDGDINDFADTHYNDVA PIADVHVD) but in v2, the negative cluster range from 526–576 and 599–668 (DLSNDLHPDNN IEQIANDHVNDIAQSTDGDINDFADTHYNDVAPIADVHVD). HRHVH is the tandemly repeated six times in v1 and HVHRH tandemly repeated three times in v2 and also 39 copies of H.HVH.H.VL periodic element present in between 50–439. Tyrosine (20.8%) is abundantly present in Mcfp5 and also the presence of positive charge clusters from 53–89 (KGKYYGKGKKYYY KYKRTGKYKYLKKARKYHRKGYKK). In Mcfp6, 3 variants are currently reported. The abundancy of tyrosine in each variant is v1(18.2%), v2 (16.5%) and v3(15.5%), and the three variants don′t contain any charge clusters. Mcfp7 v1 and v2 are almost same amino acid compositions, and also showed the same chemical structural characterizations. The tyrosine amount is 10.2% in v1 and 9.2% in v2. Glycine (27.5%) is the most abundant amino acid present in Mcfp8 and without any charge clusters and specified tandem repeats. Glycine and histidine are the abundant amino acid in both variants of Mcfp9. GGHH repeated four times in v2 and two times in v1. By analyzing the amino acid composition in Mcfp10–18 variants, each variant exhibits the unique amino acid characterization. Mcfp11 contained the mixed charge clusters from 357–386 (ENQHKRHL REREYQNKRHLSNEEHLHNKHE), positive charge clusters in Mcfp12 (231–257, RFRRFKIRHGR FRYGGKYYKLSCNKRR) and other variants doesn′t exist any charge clusters.

Two fps reported in Mefp, comparing both variants tyrosine abundantly present in Mefp1 (18.2%) and Mefp2 (7.3%). The tandem repeat distribution in fp1 is PVYKP (two times), YKPKI (four times) and in fp2, GKTGYKC (two times), KPNPC (seven times), NACKPN (five times), VCSPNP (five times) and KPNPC (three times). And these variants don′t contain any charge clusters.

The three-foot proteins reported in Mgfp (Mgfp1, Mgfp3 v1, and v2). Tyrosine abundance same in both variants of fp3 (14.3%) and in fp1 is 19.0%. Absence of tandem repeats in the fp3 v1 and v2, tandem repeats in fp1 is TYKPKPSYPATYKSKSSY (three times) and TYKPKPSYPAT YKSKSSYPSSYKPKKTY (three times). The charge clusters are absent in Mgfps.

In Mufp, major foot protein is fp2, fp3 (15 variants) and fp6 (ten variants). Amino acid composition in fp2, lysine is the most abundant (14.1%) and followed by cysteine (13.4%). This protein contained negative charge clusters from 18–37 (TAPTTQYDDDEDDYKPDTAY) and tandem repeats are KPNPC (4 times). In fp3, glycine (20.8%) is the most abundant amino acid and followed by tyrosine (18.2%) and this protein doesn't contain any specified charge clusters and tandem repeated blocks. The variants of fp3 (v1 to v14), glycine is the most abundant amino acid present in each variants v1 (20.5%), v2 (20.5%), v3 (15.2%), v4 (20.5%), v5 (20.5%), v6 (19.5%), v7 (20.5%), v8 (21.8%), v9 (20.5%), v10 (20.5%), v11 (20.0%), v12 (20.5%), v13 (22.5%) and v14 (20.8%). The tyrosine composition in each variants is v1 (19.2%), v2 (19.2%), v3 (13.6%), v4 (19.2%), v5 (17.9%), v6 (19.5%), v7 (16.7%), v8 (14.1%), v9 (17.9%), v10 (12.8%), v11 (16.2%), v12 (17.9%), v13 (7.5%) and v14 (15.3%). These variants don't contain any specified charge clusters and tandem repeated blocks. Interestingly tyrosine (19.7%) abundantly observed in fp6 and followed by glycine (12.3%). Specified charge cluster absent in this protein and the tandem repeated sequences is NCNSYAGCCL (repeated 2 times) and YCTNKGC (2 times). Fp6 has 9 variants (v1 to v9), tyrosine is the most abundant amino acids in each variant in order to v1 (19.5%), v2 (21.1%), v3 (19.5%), v4 (20.3%), v5 (20.3%), v6 (19.5%), v7 (19.6%), v8 (21.6%) and v9 (20.8%). Perfectly matched tandem repeated blocks present in variants like v2 – RGYC (two times) and v5 – RGYC (two times). And charged cluster is absent in all variants of fp6.

Two variants present in Myfp1, threonine (37.4%) is most abundantly present in v1 and in the case of v2 is glycine (26.9%). The tyrosine concentration in each variant is v1 (0.4%) and v2 (4.0%). Only v2 contained the mixed charge clusters from 2–24 (DAGFEALKKIIVRMDETERY KRR). The specified tandem repeated blocks in v1 is TSQTDT (nine times), TDTTQN (ten times), TDTTQNT TSQ (five times), QNTTSQ (eight times), NTTSQT (nine times), TSQTDTR (three times), TSQTDTT (five times), RQNTTP (nine times), DITQN (two times) and TSQTDTK (two times). In the case of v2, YGLG (seven times), YGLGQSPG (six times), YGLGQSPGTGYWLGQ SPGTG (four times) and YGLGQSPGTGYWLGQSPGTGYGLGQSPGTVYGLGQSPGTGYWL G (three times).

Under the Pcfp1, currently four variants are reported (v1 to v4). The most abundant amino acid in v1 is lysine (23.4%), and in other v2 (lysine-23.4%), v3 (lysine-24.3%), v4 (24.2%). The tyrosine abundancy in each variant is v1 (20.2%), v2 (20.1%), v3 (21.0%) and v4 (20.9%). Tandem repeated elements in v1- KPYV (88 times), v2- KPYV (87 times), v3- KPYV (91 times) and v4 – KPYV (87 times). And this variant doesn't contain any charge clusters.

In Pvfp, currently available major foot protein is fp1, fp3, fp5, and fp6. Two variants present in fp1, proline (19.6%) is abundantly present in v1 and followed by alanine (18.7%) and in v2- proline (19.5%) and then alanine (18.6%). The tyrosine level in v1 is 1.4% and in v2 is 1.6%. The tandem repeated blocks in v1 are HPPSWTAWIA (4 times), WTAWKAHPPAWTAWK (5 times), PPPAWTAWK (8 times), GKPGKPG (3 times) and PPPAWTAWKATLKPWTAWKATPKPW TAWKATPKPWTAWKATPKPWTAWK (3 times). PPPAWTAWK (9 times), GKPG (4 times) and PPAWTAWKATPKPWTAWKAP (4 times) are the tandem repeated block present in v2. Both variants don't contain any charge cluster regions. In fp3, cysteine (15.7%) is the most abundantly present amino acid and followed by phenylalanine (8.6%). 4.3% of tyrosine present in fp3 and absence of specified charge cluster regions and tandem repeated blocks. Cysteine (16.5%) is the most abundant amino acid in the fp5 and tyrosine abundancy is 14.2%. The tandem repeated sequence is GYYGKNCQ (2 times), TCKC (2 times) and CLNGG (2 times) and without any charge cluster regions. Cysteine (13.9%) is the most abundant amino acid present in fp6 and followed by glycine (10.7%) and also the presence of 6.6% of tyrosine. Fp6 without any specified tandem repeated blocks and charge clusters.

The amino acid composition of each Mfps, glycine, and lysine are the major component of the Mfps other than tyrosine (Detailed data in Supplementary File S4). Recently discovered as multiple pairs of Dopa-lysine contribute to the critical underwater adhesion^5,6,14,18^. The polymorphism in Mfps, may indicated as the versatility of adhesion as variety forms of an adhesive protein can interact the various surfaces.

### Physiochemical characterization of Mfps

**Figure 3:**
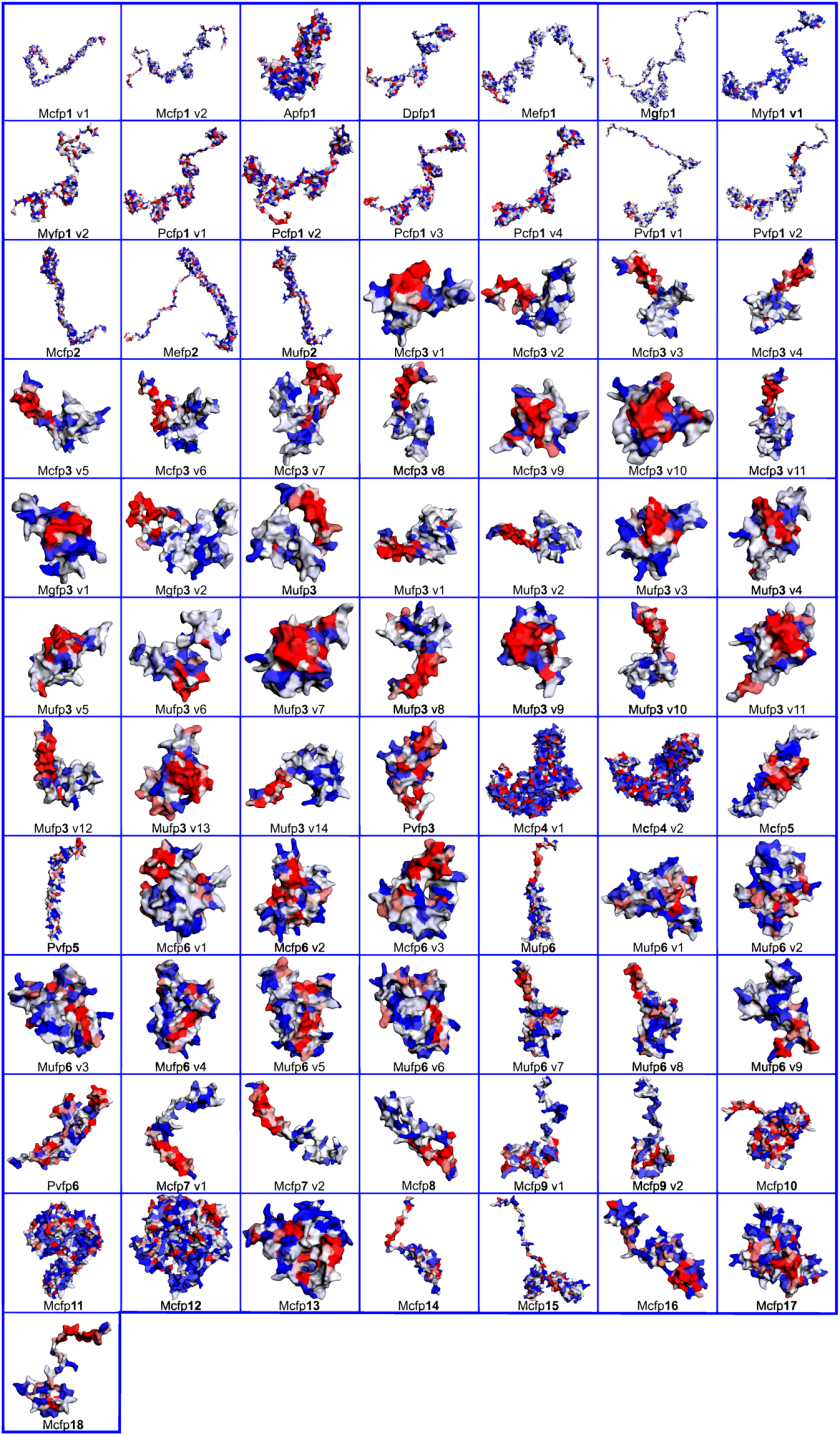
Hydrophobic contour map of mussel foot proteins (Mfps), The color code indicated as Red: High/Positive, Light color: Neutral and Blue: low/negative hydrophobicity. Generated in EzMol 2.1.

Expasy protparam server revealed the physio-chemical properties of each protein (Table 3 and Fig. 3). This server helps to the grouping of Mfps, the all Mfps half-life more than 10 hours in *E.coli* and >20 hours in yeast. In Mcfp1 v1 and v2 have the same isoelectric point 10.04, molecular weight in v1 (85024.22 D) and in v2 (78048.08 D), and other characteristic features like Instability Index (II), AI (Aliphatic Index), Extinction Coefficient (EC) and Grand Average of Hydropathicities (GRAVY) of first variant is 41.20, 26.57, 204255 and −1.357. And the second variant characters are 40.93 (II), 28.07 (AI), 186375 (EC) and −1.329 (GRAVY). Mcfp2 physiochemical properties are 9.04 (pI), 46753.52 D (Mw), 14.42 (II), 27.80 (AI), EC (58545) and −0.890 (GRAVY). The 11 variants of fp3, the highest isoelectric point was observed in v1 (10.09) and followed by v2 (10.05) and the lowest pI in v7 (7.88). The other protein in Mcfp, all variants of fp4, fp7 and fp13 the pI value is higher than 10.00. Most of the fps, II values are below 40 except the variants of fp1, fp4, fp6, fp12, fp13, fp14, fp15 and fp18. By analyzing the hydropathicity values (GRAVY analysis), all fps in Mcfps showed the non-polar nature.

Comparing the fp1 and fp2 of Mefp, only some slight difference is observed. The pI and AI of fp1 and fp2 almost similar. But the fp2 (54459.22 D) is higher molecular weight protein comparing to fp1 (6467.22 D). The hydropathicity nature of fp1 (−1.280) is higher than fp2 (−0.896).

The Mufp is the highly polymorphic group, the highest pI value observed in Mufp3 v3 (10.33) and followed by fp3 v14 (10.05). among the 26 Mfps, 15 fps molecular weight is below 10000 D and II values of fp3 v3 (42.06), fp6 v3 (40.29), fp6 v4 (40.58) and fp6 v8 (40.19) is moderately stable because the instability index is above 40. The highest hydropathicity observed in fp6 v9 (−0.938) and followed by fp3 v3 (−0.889) and fp2 (−0.842), and lowest GRAVY in fp3 v10 (−0.110).

Apfp1, Dpfp1 foot proteins are least polymorphic and less explored groups, the pI value in Apfp1 is 9.47 and pI of Dpfp1 is 5.24. In Apfp1 physiochemical features are Mw is 40382.27 D, II (33.47), AI (57.76), EC (121185) and GRAVY (−0.662). 49361.70 D molecular weighted Dpfp1, II is 53.65, AI (31.40), EC (122860) and GRAVY (−1.331). Myfp1 contained two variants (v1 and v2), but entirely different physiochemical characteristic features. The physiochemical features of v1, pI (5.12), Mw (56961.06 D), II (23.62), AI (6.97), EC (8480) and GRAVY (2.038). pI (8.66), Mw (33579.64 D),II (62.60), AI (56.48), EC (118370) and GRAVY (−0.615) is the physio-chemical feature of v2.

Comparing the all Mfps, instability index of four variants of Pcfp1 is negative (v1= −10.03, v2= −9.97, v3 = −10.61 and v4 = −10.38) and the AI is high (v1 = 65.96, v2 = 65.90, v3 = 66.83 and v4 = 66.60). Molecular weight is higher in v1 (51565.91) and lower in v4 (46301.65). Both v1 and v2 share the same hydropathicity nature and also in the case of v3 and v4. In Pvfp, the 2 variants of fp1 (v1 and v2) share the same physiochemical natures. Comparing the other fps, fp3 (0.260) and fp6 (0.102) is polar nature protein because the GRAVY value is positive in nature. Except for fp3 (44.26), other variants are highly stable because of the II is below 40.

The physiochemical structural and functional characterization of all Mfps is the first time. All protein is hydrophobic nature but except the Pvfp3 and Pvfp6 is polar but it is hydrophobic nature. In *Mytilus sp*. Mfp-3f is polar but hydrophobic nature, the protein may play vital role in metal and mineral surface adhesion^7,18,19^.

### Ion ligand binding sites of Mfps

Understanding the general properties of the ligand-binding ability of the protein sites is the great importance to understand the functional diversity of the Mfps. One of the fundamental features of the Mfps receptor surface is the set of amino acids available for interactions with ligands. The stabilization and interlinking of Mfps mainly mediated by metal ions, by divulging the metal and acid radical ion binding ability helps to understanding the functional diversity of Mfps. (Detailed predicted binding residues (s) of each Mfps provided in Supplementary File S5).

Foot protein: Apfp1, mainly 3 ligand binding sites were identified in this protein i.e., Zn^2+^, Ca^2+^ and Na^+^. Among these ligand binding sites; 67 sites are available for zinc-binding and followed by 22 sites for sodium binding and three amino acid sites for calcium (I249 E255 E341). Except for calcium, the zinc and sodium bind to the tyrosine and they may associate with DOPA to help them interlinking. No binding site detected for the following ions:(Cu^2+^, Fe^2+^, Fe^3+^, Mg^2+^, Mn^2+^, K^+^, CO_3_^2−^, NO_2_^−^, SO_4_^2−^, PO_4_^3−^).

Foot protein: Dpfp1, the four metal ions binding sites were identified in this foot protein (Zn^2+^, Ca^2+^, Na^+^ and K^+^). The binding sites of each metal ions are, Zn^2+^ binding site contained 44 sites and followed by Ca^2+^ binding site (ten sites): P174 N243 D267 K269 D289 D293 G316 P317 P402 Y403, Na^+^ binding site: C8 and K^+^ binding site (three binding sites): D267 P272 I276. No binding site detected for the following ions:(Cu^2+^, Fe^2+^, Fe^3+^, Mg^2+^, Mn^2+^, CO_3_^2−^, NO_2_^−^, SO_4_^2−^, PO_4_^3−^).

Foot proteins: Mcfps, the ligand-binding sites of each Mfps and their variants showed extremely unique features. In Mcfp1 v1, 145 binding sites were predicted for Zn^2+^, among these sites most of the zinc ions prefer the histidine amino acid for binding. Only two binding sites were available in Ca^2+^ (D458 E461). Approximately 292 sites for Na^+^ metal ion binding are present in this protein, comparing the other metal-ligand ions 2/3^rd^ portion of the amino acid in the protein capable to bind the Na^+^. The Na^+^ mostly prefer the histidine for the binding. The K^+^ metal ion binding sites are, M75 S79 I82 M83 H86 L99 H102 V103 V108. No binding site detected for the following ions:(Cu^2+^, Fe^2+^, Fe^3+^, Mg^2+^, Mn^2+^, CO_3_^2−^, NO_2_^−^, SO_4_^2−^, PO_4_^3−^). Mcfp1 v2, approximately 269 binding sites of Zn^2+^ is present, the histidine and tyrosine are the most common binding sites of Zn^2+^ metal ion. The other metal-binding sites are, Cu^2+^ binding site: H150 H152, Ca^2+^ binding site: D558 D561, Mn^2+^ binding site: H212 Q257 H260 H262, K^+^ binding site: H50 S51 and in Na^+^, approximately 186 metal ions sites were detected. No binding site detected for the following ions:(Fe^2+^, Fe^3+^, Mg^2+^, CO_3_^2−^, NO_2_^−^, SO_4_^2−^, PO_4_^3−^). Comparing the two variants of Mcfp1, the v2 contained different ligand binding sites such as Zn^2+^, Cu^2+^, Ca^2+^, Mn^2+^, K^+^ and Na^+^. The metal ion Na^+^ is mostly preferring the v1 and the Zn^2+^ prefers the v2.

Mcfp2: in the foot protein only two metal ion binding sites are present and also one acid radical ion sites also detected. Comparing the Zn^2+^ and Na^+^ metal ion binding sites, Zn^2+^ (~221 sites) is widely distributed or binding the most of the regions and followed by Na^+^ (~103 sites) and Ca^2+^ (97 binding sites). The Zn^2+^, Na^+^ and Ca^2+^ randomly bind the different amino acids present in the protein. The acid radical ion, SO^2−^ binding site: K206 R220 P221. No binding site detected for the following ions:(Cu^2+^, Fe^2+^, Fe^3+^, Mg^2+^, Mn^2+^, K^+^, CO_3_^2−^, NO_2_^−^, PO_4_^3−^).

The Mcfp3 contained 11 variants, each variant contained unique ligand binding sites were identified. In v1, mainly three metal ion binding sites and two acid radical ions were detected. The metal ion binding sites are, Zn^2+^ binding site (11 sites): Q21 D23 Y28 Y38 K39 N43 Y45 R47 Y50 W56 W61, Ca^2+^ binding site (Seven sites): L12 I15 G26 N27 G48 Y50 G51, Mg^2+^ binding site (two sites): R60 W61 and the acid radical binding sites are, CO_3_^2−^ binding site (five sites): L12 V13 I15 R63, SO_4_^2−^ binding site (11 sites): D23 R47 W52 W56 K57 K58 G59 R60 W61 K64 Y65. No binding site detected for the following ions:(Cu^2+^, Fe^2+^, Fe^3+^, Mn^2+^, Na^+^, K^+^, NO_2_^−^, PO_4_^3−^). Five different metal ions are ability to bind to the v2 (Zn^2+^, Ca^2+^, Mg^2+^, Na^+^, and K^+^). The metal ion binding sites of each metals are, Zn^2+^ binding site (14 sites): Q21 D23 K28 Y34 Y38 G39 Y42 Y48 R50 Y52 W54 K56 W58 W63, Ca^2+^ binding site (three site): G16 S22 G61, Mg^2+^ binding site (two sites): Y27 K41, Na^+^ binding site: Y48, K^+^ binding site (fives sites): K2 S3 S5 I6 L9, No binding site detected for the following ions:(Cu^2+^, Fe^2+^, Fe^3+^, Mn^2+^, CO_3_^2−^, NO_2_^−^, SO_4_^2−^, PO_4_^3−^). In v3, metal ion ligand binding sites are, Zn^2+^ binding site (23 sites): K3 Q21 D23 Y26 Y28 Y38 N39 Y42 Y45 N46 G47 Y48 Y51 H52 Y55 G56 W57 K59 W61 N62 W66 Y70 Y71, Ca^2+^ binding site (six sites): G56 W57 N58 G60 W61 N62, Na^+^ binding site (three sites): Y42 Y48 Y51. No binding site detected for the following ions:(Cu^2+^, Fe^2+^, Fe^3+^, Mg^2+^, Mn^2+^, K^+^, CO_3_^2−^, NO_2_^−^, SO_4_^2−^, PO_4_^3−^). The ligand-binding sites of v4: Zn^2+^ binding site (19 sites): K3 Q21 D23 D27 Y28 Y38 N39 Y45 Y48 Y51 H52 Y55 G56 K59 W61 N62 W66 Y70 Y71, Ca^2+^ binding site (six sites): D23 D27 W57 N58 W61 G64, Na^+^ binding site (two sites): Y48 Y51, K^+^ binding site (two sites): D23 D27, and the acid radical PO^3−^4 binding site (two sites): N58 G64. No binding site detected for the following ions: (Cu^2+^, Fe^2+^, Fe^3+^, Mg^2+^, Mn^2+^, CO_3_^2−^, NO_2_^−^, SO_4_^2−^). v5, ligand binding sites are: Zn^2+^ binding site (20 sites): K3 Q21 D23 Y28 Y38 N39 Y43 Y45 Y48 Y51 H52 Y55 G56 W57 K59 W61 N62 W66 Y70 Y71, Ca^2+^ binding site (six sites): G56 W57 N58 G60 W61 N62 and Na^+^ binding site (two sites): Y48 Y51. No binding site detected for the following ions:(Cu^2+^, Fe^2+^, Fe^3+^, Mg^2+^, Mn^2+^, K^+^, CO_3_^2−^, NO_2_^−^, SO_4_^2−^, PO_4_^3−^). v6, Zn^2+^ binding site (16 sites): K3 Q21 D23 Y28 Y44 G45 Y48 Y51 K52 Y54 R56 Y58 K62 W64 W68 W73, Ca^2+^ binding site (five sites): D29 G57 Y58 G59 N61, Mg^2+^ binding site (two sites): G46 K47, Na^+^ binding site: Y48 and acid radical ion, CO_3_^2−^ binding site (two sites): L30 Y32, SO_4_^2−^ binding site (six sites): Y28 N49 K52 G57 G71 R72, No binding site detected for the following ions:(Cu^2+^, Fe^2+^, Fe^3+^, Mn^2+^, K^+^, NO_2_^−^, PO_4_^3−^). v7, Zn^2+^ binding site (20 sites): K3 Q21 D23 Y26 Y28 D29 Y34 Y44 N45 Y48 Y51 Y54 Y57 H58 Y61 K65 W67 N68 N69 G70, Ca^2+^ binding site (14 sites): F4 S5 T7 D23 N40 P41 W42 G53 N55 G56 Y57 W63 N64 W67, Mn^2+^ binding site (two sites): N69 G70, Na^+^ binding site (four sites): Q21 Y54 Y57 N64, and acid radical CO_3_^2−^ binding site: Y32, No binding site detected for the following ions:(Cu^2+^, Fe^2+^, Fe^3+^, Mg^2+^, K^+^, NO_2_^−^, SO_4_^2−^, PO_4_^3−^). v8, Zn^2+^ binding site (20 sites): K3 Q21 S22 D23 Y26 Y28 Y32 Y38 N39 Y43 Y45 Y48 Y51 H52 Y55 W57 K59 W61 N62 W66, Fe^3+^ binding site (three site): S22 D23 Y26, Mn^2+^ binding site (eight sites): L17 A19 V20 S22 D23 A24 Y26 Y28, Na^+^ binding site (three sites): Y42 Y48 Y51, K^+^ binding site (11 sites): I15 L17 F18 A19 V20 S22 D23 A24 Y26 Y28 Y32. No binding site detected for the following ions:(Cu^2+^, Fe^2+^, Ca^2+^, Mg^2+^, CO_3_^2−^, NO_2_^−^, SO_4_^2−^, PO_4_^3−^). v9, Zn^2+^ binding site (20 sites): K3 Q21 D23 Y26 Y28 Y38 N39 Y42 Y43 Y45 N46 Y48 Y51 H52 Y55 W57 K59 W61 N62 W66, Mg^2+^ binding site (two sites): D23 H52, Mn^2+^ binding site (three sites): L9 L12 V13, Na^2+^ binding site (two sites): Y48 Y51, and acid radical SO_4_^2−^ binding site (four sites): L12 V13 G16 Y38, PO_4_^3−^ binding site (four sites): F18 A19 V20 Y42, No binding site detected for the following ions:(Cu^2+^, Fe^2+^, Fe^3+^, Ca^2+^, K^+^, CO_3_^2−^, NO_2_^−^). v10, Zn^2+^ binding site (18 sites): K3 Q21 D23 Y26 Y28 Y38 N39 Y43 Y45 Y48 Y51 H52 Y55 W57 K59 W61 N62 W66, Ca^2+^ binding site (seven sites): V13 I15 N39 G44 N46 Y55 W57, Mg^2+^ binding site (two sites): D23 H52, Na^+^ binding site (three site): Y42 Y48 Y51, No binding site detected for the following ions:(Cu^2+^, Fe^2+^, Fe^3+^, Mn^2+^, K^+^, CO_3_^2−^, NO_2_^−^, SO_4_^2−^, PO_4_^3−^). v11, Zn^2+^ binding site (18 sites): K3 Q21 D23 Y28 Y38 N39 Y43 Y45 Y48 Y51 H52 Y55 G56 W57 K59 W61 N62 W66, Fe^3+^ binding site (two sites): D23 G27, Ca^2+^ binding site (two sites): D23 G27, Mg^2+^ binding site (two sites): D23 H52, Mn^2+^ binding site (two sites): D23 G27, Na^+^ binding site (five sites): Y42 Y43 Y45 Y48 Y51, No binding site detected for the following ions:(Cu^2+^, Fe^2+^, K^+^, CO_3_^2−^, NO_2_^−^, SO_4_^2−^, PO_4_^3−^). Among the variants of Mcfp3, only v8 and v11 have the Fe^3+^ binding sites.

Ligand binding analysis of Mcfp5, a totally three metal ions and one acid radical ion binding site were predicted. The Zn^2+^ binding site (28 sites): K2 C5 C18 D20 S23 D26 Y28 D30 Y32 Y33 N39 Y40 P41 G43 H45 G46 Y47 H48 G49 H50 Y52 K53 Y57 K59 H83 Y87 Y90 Y91, Mg^2+^ binding site:K85 G86, Na^+^ binding site: N39 S44 K53, and acid radical CO_3_^2−^ binding site: H48 G51 K53 Y56, No binding site detected for the following ions:(Cu^2+^, Fe^2+^, Fe^3+^, Ca^2+^, Mn^2+^, K^+^, NO_2_^−^, SO_4_^2−^, PO_4_^3−^).

Mcfp6 contained three fps variants, the first variant v1contained 37 binding sites for Zn^2+^ and followed by Fe^3+^ binding site (three sites): S32 Y79 N113, Na^+^ binding site (18 sites): K34 C36 R37 G39 Y40 A64 C67 R75 P87 D88 F107 N108 C109 S111 Y112 N113 C115 C116, and acid radical SO_4_^2−^ binding site (six sites): S22 N45 C49 Y51 G52 S53. No binding site detected for the following ions:(Cu^2+^, Fe^2+^, Ca^2+^, Mg^2+^, Mn^2+^, K^+^, CO_3_^2−^, NO_2_^−^, PO_4_^3−^). The v2, 35 binding sites for Zn^2+^ and followed by Na^+^ binding site (14 sites): C36 R37 G39 Y40 A64 C67 N75 P87 Y107 D108 C109 S111 Y112 N113 and acid radical ion SO_4_^2−^ binding site (six sites): F11 I13 T14 C17 G18 I19. No binding site detected for the following ions:(Cu^2+^, Fe^2+^, Fe^3+^, Ca^2+^, Mg^2+^, Mn^2+^, K^+^, CO_3_^2−^, NO_2_^−^, PO_4_^3−^). v3, 34 binding sites for Zn^2+^ and followed by Ca^2+^ binding site (two sites): Y79 Y99, Mg^2+^ binding site (three site): N71 S75 T81, Na^2+^ binding site (18 sites): K34 C36 R37 G39 Y40 A64 C67 S75 P87 F90 Y107 D108 C109 S111 Y112 N113 C115 C116. No binding site detected for the following ions:(Cu^2+^, Fe^2+^, Fe^3+^, Mn^2+^, K^+^, CO_3_^2−^, NO_2_^−^, SO_4_^2−^, PO_4_^3−^). The first 18 amino acids of all residue showed similar ligand binding sites in the case of Zn^2+^.

Among the two variants of Mcfp7, showed similar ligand binding positions in the case of Zn^2+^. In v1, Zn^2+^ binding site (17 sites): Y28 R29 R30 Y32 K33 G34 S35 H36 S37 G39 G40 H41 H44 G45 H49 Y51 Y55, Ca^2+^ binding site (four sites): G34 S35 Y57 K58, Na^+^ binding site: S38 S42 Y51. No binding site detected for the following ions:(Cu^2+^, Fe^2+^, Fe^3+^, Mg^2+^, Mn^2+^, K^+^, CO_3_^2−^, NO_2_^−^, SO_4_^2−^, PO_4_^3−^). v2, Zn^2+^ binding site (21 sites): K27 Y28 Y32 Y35 K36 G39 S40 H41 S42 G44 G45 H46 S47 G49 G50 H51 H54 G55 G56 K57 Y61, Mg^2+^ binding site (two sites): S43 G44, Na^+^ binding site: H51, and acid radical ion CO_3_^2−^ binding site: S42 G56. No binding site detected for the following ions:(Cu^2+^, Fe^2+^, Fe^3+^, Ca^2+^, Mn^2+^, K^+^, NO_2_^−^, SO_4_^2−^, PO_4_^3−^).

Mcfp8, Zn^2+^ binding site (18 sites): P21 Y25 Y28 K36 Y37 K39 Y41 Y44 Y48 R51 Y52 H53 G55 K56 Y57 K60 Y61 K64, Ca^2+^ binding site (three sites): G46 K47 G66, Mg^2+^ binding site (two sites): V22 Y25, Na^+^ binding site: Y41, No binding site detected for the following ions:(Cu^2+^, Fe^2+^, Fe^3+^, Mn^2+^, K^+^, CO_3_^2−^, NO_2_^−^, SO_4_^2−^, PO_4_^3−^).

The Zn^2+^, Na^+^, and K^+^ capable to bind the various amino acid residue of the two variants, the Mg^2+^ only present in the v1 and Ca^2+^ is present in v2 only. Mcfp9 v1, 54 binding sites for Zn^2+^ and followed by Mg^2+^ binding site (four sites): G35 H36 H108 H111, Na^+^ binding site (14 sites): D27 G32 K34 Y53 H54 V57 H60 V64 G65 H67 W76 G78 P79 A91 and K^+^ binding site (nine sites): F10 G21 D29 G35 H36 V37 L38 I41 I70. No binding site detected for the following ions:(Cu^2+^, Fe^2+^, Fe^3+^, Ca^2+^, Mn^2+^, CO_3_^2−^, NO_2_^−^, SO_4_^2−^, PO_4_^3−^). In v2, 57 binding sites of Zn^2+^ is predicted, Ca^2+^ binding site (two sites): V45 H47, Na^+^ binding site (12 sites): Y26 D27 G32 K34 G35 H36 L38 H54 G71 P72 S73 G93, K^+^ binding site (eight sites): Y23 G25 V37 L38 I41 V45 V62 I63 and acid radical ion PO_4_^3−^ binding site (three sites): H78 H80 V85, No binding site detected for the following ions:(Cu^2+^, Fe^2+^, Fe^3+^, Mg^2+^, Mn^2+^, CO_3_^2−^, NO_2_^−^, SO_4_^2−^). Zn^2+^ metal ion binding sites of two variants are almost the identical.

Mcfp10–17 doesn't contain any variants, each fps showed the unique spectacular metal ions and acid radical bindings. In v10, Zn^2+^ exhibit the 49 binding sites, Mg^2+^ binding site (eight sites): V45 S46 T169 D210 D220 D221 Y299 D300 and Na^+^ had 33 binding sites. No binding site detected for the following ions:(Cu^2+^, Fe^2+^, Fe^3+^, Ca^2+^, Mn^2+^, K^+^, CO_3_^2−^, NO_2_^−^, SO_4_^2−^, PO_4_^3−^). The v11, 141 binding sites are available for Zn^2+^ metal ion, Cu^2+^ binding site (nine sites): H127 H129 H139 H143 H149 H159 H169 H173 H179. 41 amino acid is available for Na^+^ binding site. No binding site detected for the following ions:(Fe^2+^, Fe^3+^, Ca^2+^, Mg^2+^, Mn^2+^, K^+^, CO_3_^2−^, NO_2_^−^, SO_4_^2−^, PO_4_^3−^). The v12 predicted ligand binding sites, approximately 139 amino acid can bind the Zn^2+^ metal ion. The other metal ion binding sites are, Cu^2+^ binding site (three sites): H59 H81 H93, Ca^2+^ binding site (eight sites): N196 R409 D464 H465 L473 H494 I498 K532, Mg^2+^ binding site (four sites): D28 D32 S305 I479, and in the case of Na^+^ metal ions 55 predicted binding sites are available. Only one acid radical ion can bind this variant protein, SO_4_^2−^ binding site (three sites): S34 V48 R91. No binding site detected for the following ions:(Fe^2+^, Fe^3+^, Mn^2+^, K^+^, CO_3_^2−^, NO_2_^−^, PO_4_^3−^). v13 had 23 amino acid residues showed the Zn^2+^ metal ion binding ability and followed by 18 sites available for Ca^2+^ binding. And other metal ions like Mn^2+^ binding site (six sites): L16 I21 N22 G24 R25 R85 and Na^+^ binding site (threes sites): D9 G71 G94. No binding site detected for the following ions:(Cu^2+^, Fe^2+^, Fe^3+^, Mg^2+^, K^+^, CO_3_^2−^, NO_2_^−^, SO_4_^2−^, PO_4_^3−^). v14 contained three metal ion binding sites (Zn^2+^, Ca^2+^ and Na^+^). In the case of Zn^2+^, it has 41 binding sites. And followed by 10 binding sites for each metal ions like Ca^2+^ and Na^2+^. No binding site detected for the following ions:(Cu^2+^, Fe^2+^, Fe^3+^, Mg^2+^, Mn^2+^, K^+^, CO_3_^2−^, NO_2_^−^, SO_4_^2−^, PO_4_^3−^). v15, 70 amino acid residues for Zn^2+^ binding and Mg^2+^ binding site (four sites): S71 K166 K176 K180, K^+^ binding site (two sites): G15 S16 and Na^+^ showed the 13 amino acid binding sites. No binding site detected for the following ions:(Cu^2+^, Fe^2+^, Fe^3+^, Ca^2+^, Mn^2+^, CO_3_^2−^, NO_2_^−^, SO_4_^2−^, PO_4_^3−^). v16, approximately 39 amino acid sites showed the Zn^2+^ metal ion binding ability and followed by Ca^2+^ binding site (five sites): V11 V13 E21 E31 G32, Mg^2+^ binding site (three sites): V49 R50 S64, Mn^2+^ binding site (two): K27 H29 and Na^+^ binding site (seven sites): D41 C42 C44 H45 N46 C48 D58. No binding site detected for the following ions:(Cu^2+^, Fe^2+^, Fe^3+^, K^+^, CO_3_^2−^, NO_2_^−^, SO_4_^2−^, PO_4_^3−^). v17 showed the 48 sites for Zn^2+^ binding. The other metal ions like Fe^3+^ binding site (nine sites): I61 D62 V63 G65 M66 E96 P97 Q98 W102, Ca^2+^ binding site (seven sites): R58 S60 D62 R69 K71 K72 S106, Mg^2+^ binding site: D62, Na^+^ binding site (five sites): G100 S114 P152 G194 C195 and K^2+^ binding site: C20. The two-acid radicals were identified as bind to this protein i.e., SO_4_^2−^ binding site (four sites): I61 M66 L67 P93 and PO_4_^3−^ binding site (four sites): T64 G65 M66 L67. No binding site detected for the following ions:(Cu^2+^, Fe^2+^, Mn^2+^, CO_3_^2−^, NO_2_^−^).

Foot protein: Mgfp1, fps showed only two metal ions binding ability i.e. Zn^2+^ and Na^+^. Only one amino acid is available for Na^2+^ binding (S22) and in the case of Zn^2+^ had 21 binding sites. No binding site detected for the following ions:(Cu^2+^, Fe^2+^, Fe^3+^, Ca^2+^, Mg^2+^, Mn^2+^, K^+^, CO_3_^2−^, NO_2_^−^, SO_4_^2−^, P_4_O^3−^). The Mgfp3 contained two variants of fps. Only v2 showed the five-metal ion ligand binding site but in the case, v1 had only three metal ion binding been present. In v1, 12 sites for Zn^2+^ binding and followed by 20 sites for Ca^2+^ binding and in Na^+^ binding site (two sites): Y38 Y50. No binding site detected for the following ions:(Cu^2+^, Fe^2+^, Fe^3+^, Mg^2+^, Mn^2+^, K^+^, CO^2−^3, NO^−^2, SO_4_^2−^, PO_4_^3−^). In the case of v2, 18 sites were identified for Zn^2+^ binding and followed by Ca^2+^ binding site (seven sites): S25 D26 G54 Y55 G56 G57 Y58, Mg^2+^ binding site (four sites): N31 G33 G42 R46, Na^+^ binding site (two sites): W38 W61 and K^+^ binding site (two sites): D23 S25. No binding site detected for the following ions:(Cu^2+^, Fe^2+^, Fe^3+^, Mn^2+^, CO_3_^2−^, NO_2_^−^, SO_4_^2−^, PO_4_^3−^).

In Mefps-ligand binding analysis revealed the fp2 has shown the maximum ligand binding amino acid residue is present. Mefp1 had only three kinds of metal ions binding ability, Zn^2+^ binding site (15 sites): C9 C12 T15 D17 H30 Y34 Y94 Y163 Y193 P217 Y275 P279 Y311 P351 Y357, Fe^3+^ binding site (two sites): K324 S494 and Mg^2+^ binding site: G3. No binding site detected for the following ions:(Cu^2+^, Fe^2+^, Ca^2+^, Mn^2+^, Na^+^, K^+^, CO_3_^2−^, NO_2_^−^, SO_4_^2−^, PO_4_^3−^). In Mefp2, ~262 amino acids exhibited the Zn^2+^ metal ion binding ability and followed by 135 binding sites for Na^+^ metal ion. The acid radical ion SO^2−^, binding site (five sites): S296 V302 C305 Y406 G408. No binding site detected for the following ions:(Cu^2+^, Fe^2+^, Fe^3+^, Mg^2+^, Mn^2+^, K^+^, CO_3_^2−^, NO_2_^−^, PO_4_^3−^).

In Mufp2, ~142 amino acids in this protein available for Zn^2+^ metal ion binding. Fe^3+^ binding site (two sites): P89 N94, 26 sites were identified for Ca^2+^ binding and followed by 46 binding sites for Na^+^ metal ion. The acid radical CO_3_^2−^ binding site (two sites): N130 R131. No binding site detected for the following ions:(Cu^2+^, Fe^2+^, Mg^2+^, Mn^2+^, K^+^, NO_2_^−^, SO_4_^2−^, PO_4_^3−^). The Mufp3, 20 amino acid residues showed the ability to bind the Zn^2+^ metal ion and Na^+^ binding site (two sites): Y47 Y50. No binding site detected for the following ions:(Cu^2+^, Fe^2+^, Fe^3+^, Ca^2+^, Mg^2+^, Mn^2+^, K^+^, CO_3_^2−^, NO_2_^−^, SO_4_^2−^, PO_4_^3−^). The variants of fp3, the v1 and v2 had only metal ion binding ability but the v3 showed other than metal ion binding ability, also showed acid radical ion binding. The ligand-binding sites of v1 and v2 are almost similar. The metal ion binding sites of v1: 23 sites were available for Zn^2+^ binding and followed by Ca^2+^ binding site (10 sites): Y29 N39 Y42 Y43 Y45 N46 G47 G50 W57 W61 and two sites available for Na^+^ binding (Y48 Y51). No binding site detected for the following ions:(Cu^2+^, Fe^2+^, Fe^3+^, Mg^2+^, Mn^2+^, K^+^, CO_3_^2−^, NO_2_^−^, SO_4_^2−^, PO_4_^3−^). v2, contained 20 sites for Zn^2+^ binding and followed by 13 sites for Ca^2+^ binding and two binding sites for Na^+^ binding site (Y48 Y51). No binding site detected for the following ions:(Cu^2+^, Fe^2+^, Fe^3+^, Mg^2+^, Mn^2+^, K^+^, CO_3_^2−^, NO_2_^−^, SO_4_^2−^, PO_4_^2−^). v3, Zn^2+^ binding site (12 sites): N3 Q21 D23 Y28 N32 Y38 K39 R47 Y50 W52 W56 W61, Mg^2+^ binding site (three sites): G62 R63 K64, Mn^2+^ binding site (three sites): A11 L14 I15 and acid radical CO_3_^2−^ binding site (two sites): I6 L10. No binding site detected for the following ions:(Cu^2+^, Fe^2+^, Fe^3+^, Ca^2+^, Na^+^, K^+^, NO_2_^−^, SO_4_^2−^, PO_4_^3−^). The ligand-binding sites of other variants of fp3 shared the similar ligand- binding sites. In v4, 25 binding sites are predicted in the case of Zn^2+^ metal ions and followed by the Ca^2+^ binding site (eight sites): G56 W57 N58 G60 W61 N62 Y70 L77, Mn^2+^ binding site (three sites): L9 L12 V13 and Na^+^ binding site (three sites): Y42 Y48 Y51. No binding site detected for the following ions:(Cu^2+^, Fe^2+^, Fe^3+^, Mg^2+^, K^+^, CO_3_^2−^, NO_2_^−^, SO_4_^2−^, PO_4_^3−^). The v5, contained 26 binding sites for Zn^2+^ binding and followed by Ca^2+^ binding site (seven sites): G56 W57 N58 G60 W61 N62 Y70. Na^2+^ binding site: Y42 Y48 Y51. No binding site detected for the following ions:(Cu^2+^, Fe^2+^, Fe^3+^, Mg^2+^, Mn^2+^, K^+^, CO_3_^2−^, NO_2_^−^, SO_4_^2−^, PO_4_^3−^). In v6, 24 binding sites for Zn^2+^ metal ion, and followed by the Ca^2+^ binding site (six sites): G40 G43 Y44 S45 G46 G49, Na^+^ binding site: Y38. No binding site detected for the following ions:(Cu^2+^, Fe^2+^, Fe^3+^, Mg^2+^, Mn^2+^, K^+^, CO_3_^2−^, NO_2_^−^, SO_4_^2−^, PO_4_^3−^). The v7, 23 binding sites are accessible for Zinc binding, and followed by Ca^2+^ binding site (three sites): W57 W61 Y70 L77, Na^2+^ binding site (three sites): Y42 Y48 Y51 and acid radical CO_3_^2−^ binding site (two sites): R54 G56 and SO_4_^2−^ binding site (three sites): N46 K59 G68. No binding site detected for the following ions:(Cu^2+^, Fe^2+^, Fe^3+^, Mg^2+^, Mn^2+^, K^+^, NO_2_^−^, PO_4_^3−^). In v8, 19 predicted sites for Zn^2+^, and followed by Mg^2+^ binding site (two sites): Y51 K62, Na^+^ binding site (three sites): Y42 Y48 Y51. No binding site detected for the following ions:(Cu^2+^, Fe^2+^, Fe^3+^, Ca^2+^, Mn^2+^, K^+^, CO_3_^2−^, NO_2_^−^, SO_4_^2−^, PO_4_^3−^). The v9, showed the five different metal ions has the ability to bind this protein (Zn^2+^, Ca^2+^, Mn^2+^, Na^+^, and K^+^). 20 amino acid residues capable to bind the Zn^2+^ metal ion and followed by Ca^2+^ binding site (three sites): W57 N58 W61, Mn^2+^ binding site (three sites): I6 L9 L10, Na^+^ binding site (two sites): Y48 Y51. K^+^ binding site (six sites): L12 L14 N39 N46 W57 K59. No binding site detected for the following ions:(Cu^2+^, Fe^2+^, Fe^3+^, Mg^2+^, CO_3_^2−^, NO_2_^−^, SO_4_^2−^, PO_4_^3−^). In v10, 17 sites for Zn^2+^ binding and two sites for Na^+^ binding (Y48 Y51). No binding site detected for the following ions:(Cu^2+^, Fe^2+^, Fe^3+^, Ca^2+^, Mg^2+^, Mn^2+^, K^+^, CO_3_^2−^, NO_2_^−^, SO_4_^2−^, PO_4_^3−^). The v11 contained 21 sites for Zn^2+^ binding and followed by five sites for Ca binding (G56 N58 G60 N62 W66), four sites for Na^+^ binding (Y48 Y51 G72 N73). No binding site detected for the following ions:(Cu^2+^, Fe^2+^, Fe^3+^, Mg^2+^, Mn^2+^, K^+^, CO_3_^2−^, NO_2_^−^, SO_4_^2−^, PO_4_^3−^). In v12, showed the 22 sites for Zn^2+^ binding, and followed by other elements like Ca^2+^ binding site (three sites): L17 V20 A24, Mg^2+^ binding site (two sites): Y48 Y51, Na^+^ binding site (two sites): Y48 Y51, K^+^ binding site: L17 V20 A24, and acid radical CO_3_^2−^ binding site (two sites): R54 G56. No binding site detected for the following ions:(Cu^2+^, Fe^2+^, Fe^3+^, Mn^2+^, NO_2_^−^, SO_4_^2−^, PO_4_^3−^). In v13, 16 binding sites for Zn^2+^ binding, In the case of other metal ions, Na^+^ binding site (three sites): Y48 F51 G72 and K^+^ binding site (seven sites): L12 L14 G16 N39 N46 Y55 G57. No binding site detected for the following ions:(Cu^2+^, Fe^2+^, Fe^3+^, Ca^2+^, Mg^2+^, Mn^2+^, CO_3_^2−^, NO_2_^−^, SO_4_^2−^, PO_4_^3−^). The ligand-binding sites of v14, 14 sites for Zn^2+^ binding and followed by Ca^2+^ binding site (six sites): G25 G26 G51 Y52 G53 N55, Na^+^ binding site: Y48 and K^+^ binding site (five sites): R50 K56 G57 W58 N63. No binding site detected for the following ions:(Cu^2+^, Fe^2+^, Fe^3+^, Mg^2+^, Mn^2+^, CO_3_^2−^, NO_2_^−^, SO_4_^2−^, PO_4_^3−^).

Mufp6 contained 34 sites for Zn^2+^ binding, two sites for the Ca^2+^ binding site (Y99 G111), and 16 sites for Na^+^ binding. No binding site detected for the following ions:(Cu^2+^, Fe^2+^, Fe^3+^, Mg^2+^, Mn^2+^, K^+^, CO_3_^2−^, NO_2_^−^, SO_4_^2−^, PO_4_^3−^). The fp6 had nine variants almost all the ligand-binding sites are the same. v1, had 34 binding sites is present for Zn^2+^ metal ions and followed by 16 sites for Na^+^ binding. No binding site detected for the following ions:(Cu^2+^, Fe^2+^, Fe^3+^, Ca^2+^, Mg^2+^, Mn^2+^, K^+^, CO_3_^2−^, NO_2_^−^, SO_4_^2−^, PO_4_^3−^). In v2, ligand binding sites of each element are, 42 sites for Zn^2+^ binding, three sites for Cu^2+^ binding,13 sites for Na^+^ binding. No binding site detected for the following ions:(Fe^2+^, Fe^3+^, Ca^2+^, Mg^2+^, Mn^2+^, K^+^, CO_3_^2−^, NO_2_^−^, SO_4_^2−^, PO_4_^3−^). In v3, 35 binding sites for Zn^2+^, one binding site for Ca^2+^ (D28) and 15 binding sites for Na^+^. No binding site detected for the following ions:(Cu^2+^, Fe^2+^, Fe^3+^, Mg^2+^, Mn^2+^, K^+^, CO_3_^2−^, NO_2_^−^, SO_4_^2−^, PO_4_^3−^). In v4, 35 binding sites for Zn^2+^ and 12 binding sites for Na^+^. No binding site detected for the following ions:(Cu^2+^, Fe^2+^, Fe^3+^, Ca^2+^, Mg^2+^, Mn^2+^, K^+^, CO_3_^2−^, NO_2_^−^, SO_4_^2−^, PO_4_^3−^). The v5 contained 36 binding sites for Zn^2+^ and 13 sites for Na^+^ binding. No binding site detected for the following ions:(Cu^2+^, Fe^2+^, Fe^3+^, Ca^2+^, Mg^2+^, Mn^2+^, K^+^, CO_3_^2−^, NO_2_^−^, SO_4_^2−^, PO_4_^3−^). In v6, 39 sites for Zn^2+^ binding and 13 sites for Na^+^ binding. No binding site detected for the following ions:(Cu^2+^, Fe^2+^, Fe^3+^, Ca^2+^, Mg^2+^, Mn^2+^, K^+^, CO_3_^2−^, NO_2_^−^, SO_4_^2−^, PO_4_^3−^). v7 contained 31 binding sites for Zn^2+^, 10 binding sites for Na^+^ and three sites for K^+^ binding. No binding site detected for the following ions:(Cu^2+^, Fe^2+^, Fe^e+^, Ca^2+^, Mg^2+^, Mn^2+^, CO_3_^2−^, NO_2_^−^, SO_4_^2−^, PO_4_^3−^). In v8, 33 sites are available for Zn^2+^, four sites for Mg^2+^ binding and nine sites for Na^+^ binding. No binding site detected for the following ions:(Cu^2+^, Fe^2+^, Fe^3+^, Ca^2+^, Mn^2+^, K^+^, CO_3_^2−^, NO_2_^−^, SO_4_^2−^, PO_4_^3−^). The last variant v9, contained 32 binding sites for Zn^2+^, four sites for Ca^2+^ binding and nine sites for Na^+^ binding. No binding site detected for the following ions:(Cu^2+^, Fe^2+^, Fe^3+^, Mg^2+^, Mn^2+^, K^+^, CO_3_^2−^, NO_2_^−^, SO_4_^2−^, PO_4_^3−^).

Foot protein (Myfp1) contained two variants. In v1, 34 sites are mainly focused on the Zn^2+^ binding, 11 sites for Ca^2+^ binding and three sites for Na^+^ binding (N195 T197 S198). No binding site detected for the following ions:(Cu^2+^, Fe^2+^, Fe^3+^, Mg^2+^, Mn^2+^, K^+^, CO_3_^2−^, NO_2_^−^, SO_4_^2−^, PO_4_^3−^). The next variant v2, 31 binding sites available for Zn^2+^ binding, six sites for Ca^2+^ binding, two sites for Mn^2+^ binding (T319 Y321), Na^+^ binding site: P317 and K^+^ binding site: L233 Q235. No binding site detected for the following ions:(Cu^2+^ Fe^2+^, Fe^3+^, Mg^2+^, CO_3_^2−^, NO_2_^−^, SO_4_^2−^, PO_4_^3−^). Comparing both variants of fp1, v2 showed the different types of metal ions binding capacities.

Foot protein, (Pcfp1) contained four variants of fp1. For each protein variant, some of the binding sites of metal ions are similar. In v1, 83 sites are available for Zn^2+^ binding, two sites for Fe^3+^ binding (K143 K323), six binding sites for Ca^2+^ (P236 Y237 P330 P350 Y351 H436) and three binding sites for Na^+^ (F10 K289 Y317). No binding site detected for the following ions:(Cu^2+^, Fe^2+^, Mg^2+^, Mn^2+^, K^+^, CO_3_^2−^, NO_2_^−^, SO_4_^2−^, PO_4_^3−^). v2, 95 binding sites available for Zn^2+^, eight sites for Ca^2+^ binding (P338 P350 Y355 K367 P376 Y377 K379 H432), two sites for Na^+^ binding site (Y303 P306), and five sites for K^+^ binding (P174 K177 P178 V180 K181). No binding site detected for the following ions:(Cu^2+^, Fe^2+^, Fe^3+^, Mg^2+^, Mn^2+^, CO_3_^2−^, NO_2_^−^, SO_4_^2−^, PO_4_^3−^). The variant v3 contained 67 binding sites for Zn^2+^, two binding sites for Fe^3+^ (K153 Y179, six binding sites for Ca^2+^ (P228 Y229 P322 P326 Y327 V328) and three sites for Mg^2+^ binding (K22 K23 P24). No binding site detected for the following ions:(Cu^2+^, Fe^2+^, Mn^2+^, Na^+^, K^+^, CO_3_^2−^, NO_2_^−^, SO_4_^2−^, PO_4_^3−^). In v4, 87 binding sites for Zn^2+^, two sites for Fe^3+^ binding (Y217 K271) and four sites for Ca^2+^ binding (P224 Y225 P302 Y303). No binding site detected for the following ions:(Cu^2+^, Fe^2+^, Mg^2+^, Mn^2+^, Na^+^, K^+^, CO_3_^2−^, NO_2_^−^, SO_4_^2−^, PO_4_^3−^). Except for variant v2, other variables have the Fe^3+^ binding sites.

Foot protein (Pvfp), a total of five different types of proteins present under the Pvfps, among the five two proteins are the variants of fp1. The ligand-binding characterization of these proteins showed distinct characteristic features. In fp1 v1, contained 38 sites for Zn^2+^, two sites for Fe^3+^ binding (H547 H549), two sites for Ca^2+^ binding (D517 E528), five sites for Mg^2+^ binding (G525 K526 G548 G550 A559) and four binding sites for Na^+^ (P443 G548 H549 W551). No binding site detected for the following ions:(Cu^2+^, Fe^2+^, Mn^2+^, K^+^, CO_3_^2−^, NO_2_^−^, SO_4_^2−^, PO_4_^3−^). In fp1 v2, 27 binding sites for Zn^2+^, two sites for Fe^3+^ binding (H417 H419), two sites for Ca^2+^ binding (P123 K124) and five binding sites for Na^+^ (M5 P33 G418 H419 W421). No binding site detected for the following ions:(Cu^2+^, Fe^2+^, Mg^2+^, Mn^2+^, K^+^, CO_3_^2−^, NO_2_^−^, SO_4_^2−^, PO_4_^3−^).

Pvfp5, 80 sites are available for Zn^2+^ binding, 31 sites for Ca^2+^ binding, 22 sites for Na^+^ binding and acid radical CO_3_^2−^ binding site: C47 R48. No binding site detected for the following ions:(Cu^2+^, Fe^2+^, Fe^3+^, Mg^2+^, Mn^2+^, K^+^, NO_2_^−^, SO_4_^2−^, PO_4_^3−^). Pvfp6, 38 sites for Zn^2+^ binding (C6 E24 Q28 C29 I35 C38 C40 I41 E43 N44 S45 E46 C47 D50 N52 C53 A56 C59 C60 D61 F62 C64 C66 N67 C70 C80 G84 Y87 F93 D97 C99 C102 C104 N105 D107 C112 K115 C117) two sites for Ca^2+^ binding (C102 C112) and Na^+^ binding site: Q16 D50 S51 C53 C60 Y87 V96 D97 C99 N100. No binding site detected for the following ions:(Cu^2+^, Fe^2+^, Fe^3+^, Mg^2+^, Mn^2+^, K^+^, CO_3_^2−^, NO_2_^−^, SO_4_^2−^, PO_4_^3−^).

The catechol containing polymers and peptides has the ability to bind various metal ions like Zn^2+^, Cu^2+^, Fe^3+^, Ca^2+^, Mg^2+^, Mn^2+^, Na^+^ and K^+^ and acid radical ions, CO_3_^2−^, NO_2_^−^, SO_4_^2−^ and PO_4_^3−^. The overall analysis most of the foot proteins showed the Zn^2+^ and Na^+^ metal ion binding ability and only few Mfps have the ability to bind acid radical ions.

### Unbosoming the emergence of Bivalvia: Perspectives on mitogenome, TimeTree, and Mfps

The Bivalvia evolution is very complex in nature, the evolution of Bivalvia starts from Cambrian periods^3^. The bivalve origins, evolution of their phenotype and functional divergence of Mfps is largely remained unresolved. Evolutionary pattern of byssus thread producing Bivalvia in different perspectives revealed the functional divergence and speciation pattern.

### Mitogenome – Phylogenetic construction analysis of bio-adhesive producing bivalves

The complete mitochondrial genome sequence analysis revealed the mitochondrial genome evolutionary pattern in the byssus thread producing bivalves. Based on the mitochondrial genome evolution, the speciation of all *Mytilus* species is from the same clad but interestingly founded that *Perna perna* is also originated from the speciation node of *Mytilus*. In the genus of *Perna*, currently, three living species only exist, the stem branch of *P. canaliculus* and *P. viridis* is entirely separated from *P. perna*. The monophyletic origin of mitogenome initially separated into two branches. The one branch contained three species of Bivalvia, with entirely different order taxa, the *D. polymorpha* under the order Myoida and another are Mytiloida (*P.canaliculus* and *P.viridis*). And another set of clad, the first separated taxa is Pectinoida and then followed by Ostreoida and Myoida. The Ostreoida is closely resembling the mitogenome of Mytiloida. Among the *Mytilus* species, the *M.galloprovincialis* is the first originated species and it is the ancestor of all other *Mytilus sp*. and *P.perna* also. The *M.unguiculatus* and *M.californianus* is existed in the same clad.

**Figure 4:**
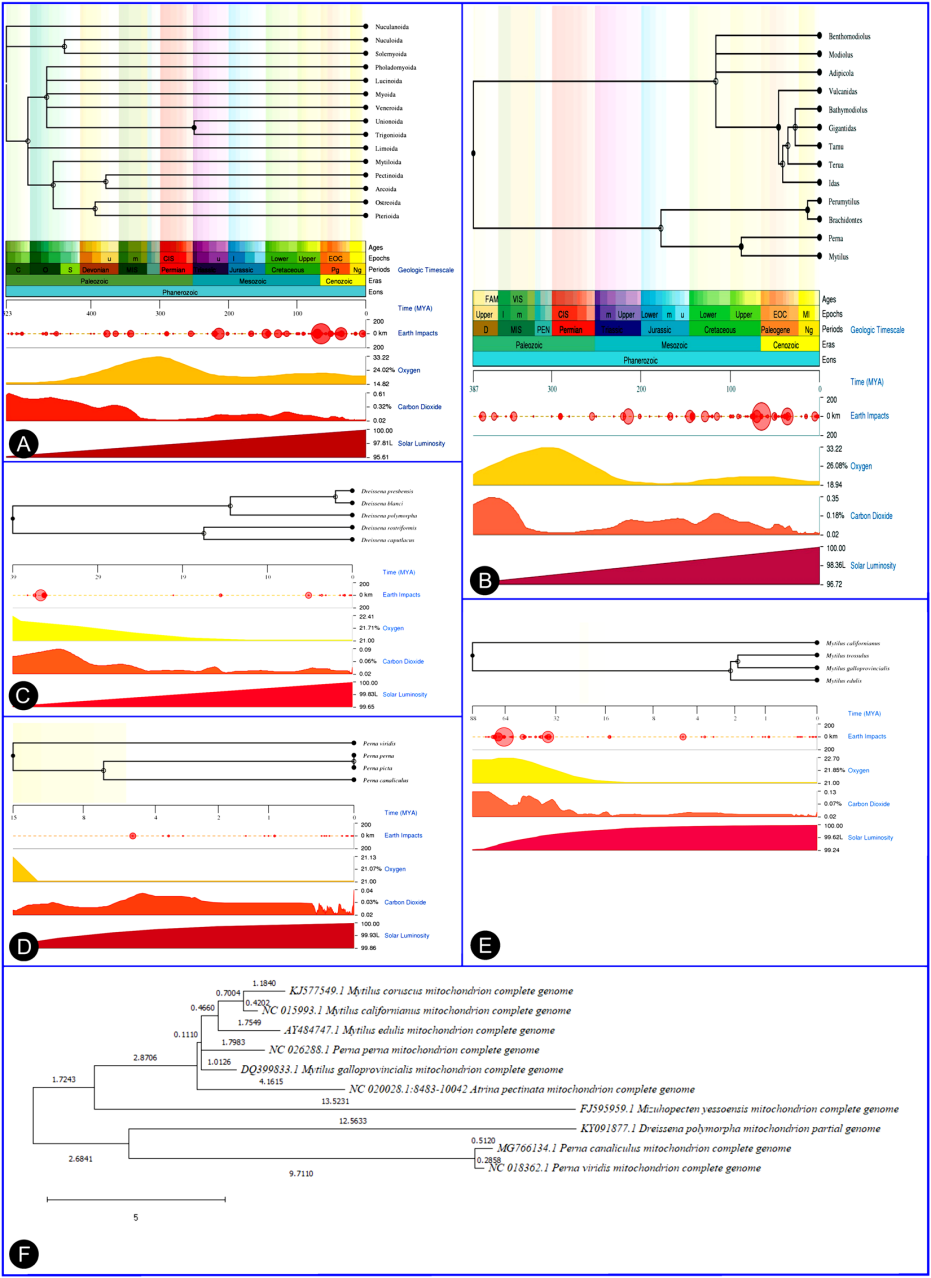
TimeTree analysis of (**A**) Bivalvia (**B**) Mytilidae (**C**) *Dreissena sp*. (**D**) *Perna sp*. (**E**) *Mytilus sp*. generated in TimeTree.org tool. (**F**) Maximum likelihood phylogenetic construction based on mitogenome, MUSCLE alignment and Tamura-Nei model – generated in MEGA X tool.

Phylogenetic speciation based on the mitochondrial genome, primitive to recently evolved byssus thread producing bivalve: *Mytilus galloprovincialis* → *Mytilus californianus* → *Perna perna* → *Mytilus unguiculatus → Mytilus edulis → Atrina pectinata → Perna viridis → Perna canaliculus→ Dreissena polymorpha → Mizuhopecten yessoensis* (Fig. 4F).

### Timetree of bivalvia

The evolutionary TimeTree of life (TTOL) it helps in understanding the origin and diversity of life forms. The clock like changes analysis, tracing out the speciation and diversification process and events. The diversification of Bivalvia order, TimeTree analysis interestingly revealed the ancestors of byssus thread producing bivalves. The evolution of Bivalvia starts from the Cambrian period (523 MYA), the first diversification/speciation (clad separation) occurred in the starting period of Ordovician (488 MYA). The majority of diversification observed from the Ordovician period. Nuculanoida order is the ancestral group, because without any diversification (clad separation) and followed by Limoida. The byssus thread producing bivalves mainly under the four orders ie, Myoida, Mytiloida, Ostreoida, and Pectinoida. The Myoida is the first diversified group (~465 MYA), evolutionarily it diverged from Mytiloida (~453 MYA). The other stem branch of Mytiloida, the diversification occurred in the Devonian periods, Ostreoida (~398 MYA) is the first diversified group comparing to the Pectinoida (~ 378 MYA). The Bivalvia evolutionary diversification analysis fascinatedly founded that only these two orders (Ostreoida and Pectinoida) are diversified during the Devonian period. After the major extinction (251 MYA), during the starting age of the Triassic period, diversification of Trigonioida and Unionoida has occurred, these are the latest evolved groups in Bivalvia (Fig. 4A).

In the case of Mytilidae time tree analysis, about 387 MYA, the initial diversification of Mytilidae started. During the Jurassic period, ~172 MYA, the genus of *Mytilus* and *Perna* is separated from the Perumytilus and Brachidontes. The diversification of clad separation of *Mytilus* and *Perna* in the Cretaceous period, upper Epochs. 88 MYA, speciation of *Mytilus* has occurred, comparing other species with *M.californianus* is present in a separate clade. The latest evolved species in the *Mytilus sp*. is *M.galloprovincialis* and *M.trossulus*. The *M. edulis* speciation occurred in 2 MYA. In about 15 MYA ago, the diversification and speciation of *Perna* are starts. The speciation of *P.perna* and *P.canaliculus* is observed in 6 MYA.

Based on the TimeTree analysis, it divulges the clock-like speciation and diversifications. The speciation event of byssus thread producing bivalve, ancestor to latest evolved order is *Dreissena polymorpha → Mytilus californianus → Mytilus edulis → Mytilus galloprovincialis → Mytilus unguiculatus → Perna viridis → Perna perna → Perna canaliculus → Atrina pectinata → Mizuhopecten yessoensis* (Fig. 4B–E).

The mitochondrial genome revealed the fascinating key of Mfps producing Bivalvia origin, *M.galloprovincialis* is the first evolved Bivalvia then followed by *M.californianus*. But, in the case of TimeTree analysis, the first evolved Bivalvia is *D.polymorpha*, it is an brackish/fresh water forms, then followed by *M.califroninanus*. Both mitogenome and TimeTree revealed the second evolved form is *M.californianus*. The natural selection behavior, molecular evolutionary clock speciation indicated as the fresh/brackish form bivalve is the first evolved form, is evidently supported by the Bivalvia taxa evolution and diversification. Contradiction outcome observed in the mitogenome analysis, under the natural selection pressure is marine bivalve to brackish/fresh aquatic forms of species diversification raised. The mitogenome is an important potential target of natural taxa selection spread across the gradients of the ecosystem^20^. The bivalve spread in the costal belt habitats with dynamic changes such as temperature fluctuation, salinity, dissolved oxygen, desiccation, UV- radiation and exposure to chemical pollutants etc., which can induce oxidative stress to them^21^, may influence the respiration of the mitochondria and cause irreversible damage to mtDNA^22^.

### Intra-phyletic evolutionary relationship of Mfps

**Figure 5:**
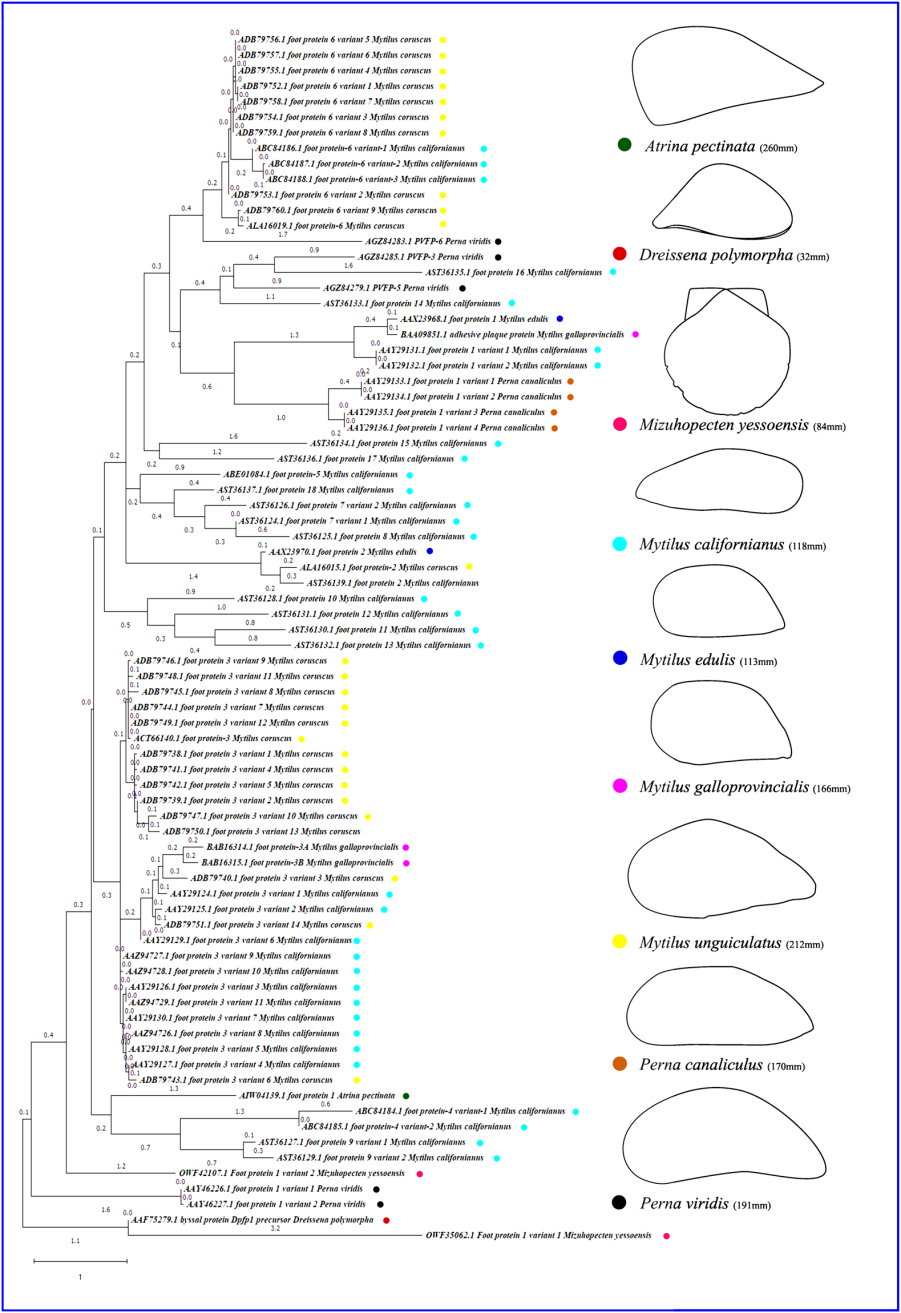
Mussel foot proteins (Mfps) based phylogenetic analysis by using the maximum likelihood and the JTT matrix model. Sequence were aligned by using MUSCLE and tree generated in the MEGA X tool. Each color code indicated as the respective bivalves (Drawing: YSV).

Insight the evolutionary pattern of foot proteins entirely different form the mitochondrial genome evolutionary pattern and TimeTree analysis. The first evolved Mfps is Mcfp3 V9 and followed by Mcfp3 v10, Mufp3, Mufp3 v7, Mufp3 v12, Mufp3 v9, Mufp3 v11, Mufp3 v8, Mcfp3 v7, Mcfp3 v3, Mcfp3 v11, Mcfp3 v8, Mcfp3 v5, Mcfp3 v4, Mufp3 v6, Dpfp1, Mufp3 v1, Mufp3 v4, Mufp3 v5, Mcfp6, Mufp3 v2, Mufp3 v10, Mufp3 v13, Mcfp3 v6, Mufp3 v14, Mcfp3 v2, Mcfp3 v1, Mufp3 v3, Myfp1 v2, Pvfp1 v1, Pvfp1 v2, Mgfp3 v2, Mgfp3 v1, Mufp6 v2, Mufp6 v4, Mufp6 v5, Mufp6 v6, Mufp6 v3, Mufp6 v8, Mufp6 v1, Mufp6 v7, Mufp6 v9, Mufp6, Mcfp6 v1, Mcfp5, Mcfp18, Mcfp6 v2, Mcfp6 v3, Mcfp10, Mcfp7 v1, Mcfp7 v2, Apfp1, Mcfp17, Mcfp9 v1, Mcfp9 v2, Mefp2, Mcfp14, Pvfp5, Mcfp12, Mcfp11, Mcfp13, Mcfp4 v2, Mufp2, Mcfp2, Mcfp8, Pcfp1 v3, Pcfp1 v4, Mcfp4 v1, Pvfp3, Pcfp1 v1, Pcfp1 v2, Mcfp1 v1, Mcfp1 v2, Pvfp6, Mefp1, Mgfp1, Mcfp16 and Myfp1 v1 is the latest evolved Mfps. The first foot protein is appeared in *M.californianus* and then followed by *M.unguiculatus*, *D. polymorpha, M. yessoensis, P.viridis, M.galloprovincialis*, *A. pectinata, M.edulis*, and *P.californianus*. Interestingly founded that natural selection divergence exists in Mfps. Because during the evolutionary patterns, the expression level of each Mfps has occurred at different time intervals based on their dynamic environmental conditions (Fig.5).

**Figure 6:**
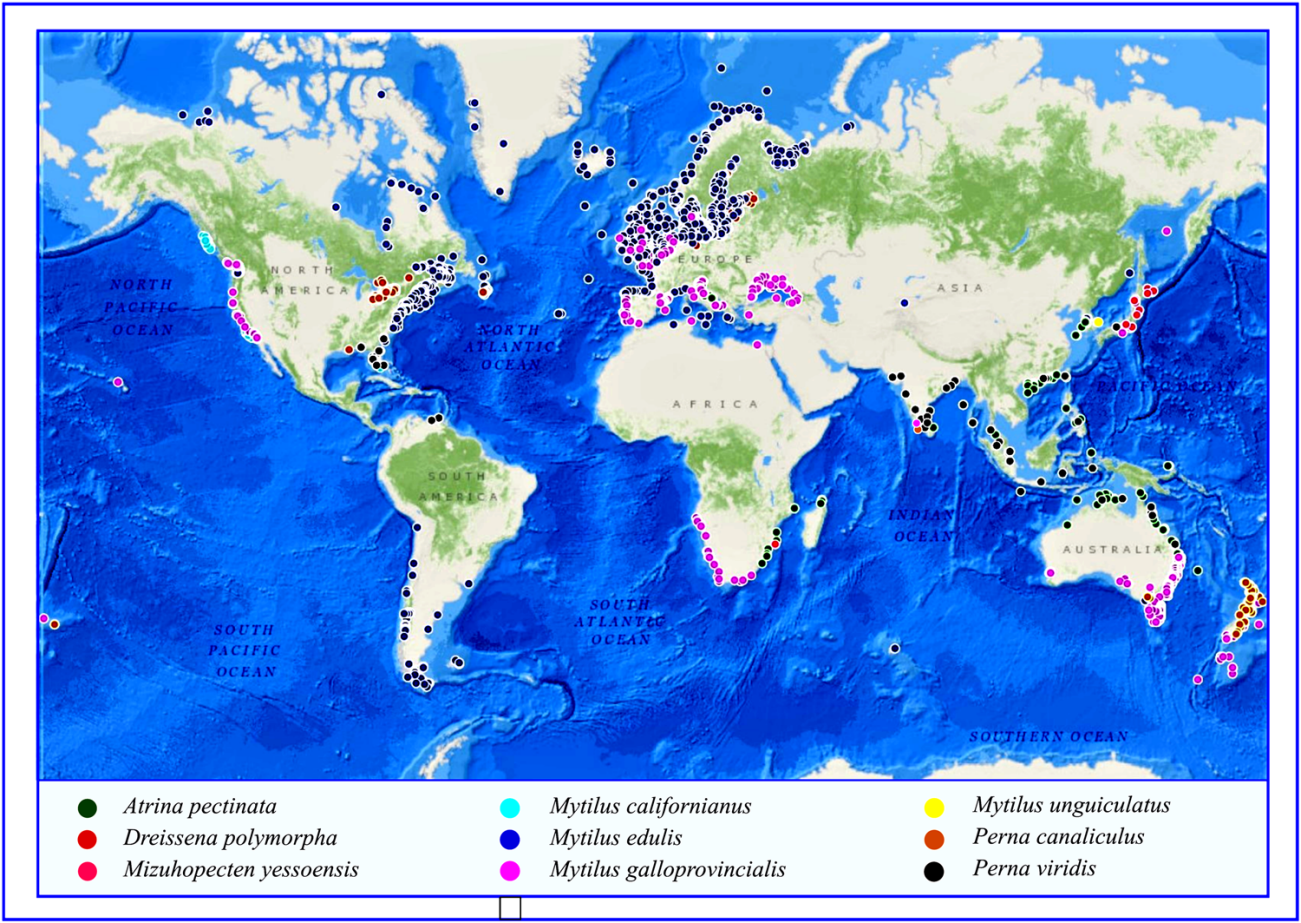
Approximate geographical distribution of selected Bivalvia species. Generated in – OBIS 2.0 server (2019) (https://mapper.obis.org/). [Available: Ocean biogeographical information system (OBIS). Intergovernmental oceanographic commission UNESCO. www.iobis.org. Accessed: 12^th^ September 2019]. (Supplementary S1).

The evolutionary and environmental forces equally blend together to tune the unique constitution, magnitude and function of each proteins in the adhesive secretion. The functional property of each protein in root, stem, thread and plaque of byssus thread are highly predisposed and extremely intricate^2,4,23^. The evolutionary lineage of Mfps revealed the sedentary mode of life style preference of an adult organisms. The Mfps property determined by the geographical habits of the organisms. These byssus threads producing bivalves is randomly distributed all over the world, each geographic zone has the specific dynamic characters are presents, in the case tidal power, salinity, temperature, wave actions etc. Based on this property the evolution and functional divergence of Mfps may evolved. The evolutionary divergence of Mfps producing bivalve, *M.edulis* is showed the highly complex geographical distribution pattern. The geographical distributional pattern of *Mytilus sp*. are widespread that exhibit an anti-tropical distribution with *M.edulis, M.californianus*, and *M.unguiculatus* occurring in the Northern Hemisphere and *M.galloprovincialis* distributed in Northern and Southern Hemispheres^24^. The geographical distribution of *Perna sp*. is mainly occurred in the tropical zone. *P. canaliculus* is randomly distributed in Southern temperate region (Fig. 6 and Supplementary data S1- Table 1).

By analyzing all Mfps, the fp3 and then followed by fp5 and fp6 is the evolutionary lineage of foot proteins in selected bivalves species (all *Mytilus sp*. except *M.edulis* fp3 is not available) except *P.viridis*. Because the fp3,5 and 6 are predominantly found in plaque region of the byssus threads and it contribute to the wet adhesion^2^. It can be easily concluded that in all byssus thread producing bivalves, fp3 is the ancestor of all other existing Mfps and the wet adhesion property is the core phenomena of all Mfps. In *Perna* sp. first evolved Mfps is fp1, they actually provide the hydrophobic nature and act as protective varnish layer^25^. Comparing to the all Mfps, the fp1 and fp2 is the last evolved Mfps, because the fp1 mainly act as protective functions and fp2, provide the structural integrity to the adhesive plaques^14^. After the fp1, fp5 and followed by fp3 and fp6 is the evolutionary lineages of wet adhesive property of Mfps in *Perna sp*. except fp3, the fp5 has the specialized mechanism for ability to bind into the calcareous mineral substrate. Fp5 contained the phosphoserine, the post translational modification of phosphoserine gives the ability to bind calcareous materials. The fp5 is the first produced foot protein by *P. viridis* for surface water replacement and then followed by fp3 and fp6 it gives the stability to wet adhesion^13^. Mfps revealed functional evolutionary origin characterization and speciation of *Perna sp*^26^. The Darwin natural selection pressure is observed in the expression of Mfps diversification because the Mfps is played a vital role in wet adhesion and helps to development of the sedentary mode of lifestyle. The natural selection depends on the environment and requires existing heritable variation in a group. This is the first report of the phylogeny construction of all available Mfps and evolutionary analysis based on the functional divergence.

## Conclusion

This is the first report by using the insilico methods to evaluate the physiochemical structural and functional characterization of all available Mfps revealed the unique characteristic features of each mussel foot proteins (Mfps). Required more than a thousand mussels for each Mfps extraction and characterization from different species of mussels in the aim of creating strong adhesives materials. In this works highlighted the several biochemicals, molecular, structural and functional features of the Mfps, these results help to the future development of bio-adhesives in different perspectives. We are not only revealed the bio-adhesive property of Mfps and also revealing the complex nature of evolutionary lineages and diversification of Mfps and selected Bivalvia species with geographical distributions.

## Materials and Methods

### Datasets

Bivalve Mfps (mussel foot proteins) sequences in FASTA format were retrieved from the NCBI protein database (August 2019) (http: www.ncbi.mlm.gov/protein). Selection criteria are mainly based on Mfps producing bivalves in which the complete sequence of at least one adhesive protein is identified.

### Molecular modeling

The MUSTER algorithm used for protein modeling^10,27^. This server (https://zhanglab.ccmb.med.umich.edu/MUSTER/) analyzes the previous sequence profile-profile alignment (PPA) method and the best template used for the homology modeling of Mfps. The models were evaluated in PROCHECK^27,28^ and PDBsum server^12,27^, and the visualization of the protein model in PyMol^27^ and EzMol 2.1^29^.

### Signal peptide prediction

Phobius (http://phobius.sbc.su.se/)^30^ and SignaIP 5.0 (http://www.cbs. dtu.dk/services/SignalP/)^31^ servers were used to analyze the signal peptide topology prediction^27^ of Mfps.

### Functional characterization of Mfps

FFPred 3 (http://bioinf.cs.ucl.ac.uk/psipred/)^17^ server used for functional characterization of Mfps. The predictions are made by scanning the input sequences against an array of Support Vector Machines (SVM). In this server, large SVM library that extends its coverage to the cellular component sub-ontology for the first time, prompted by the establishment of a dedicated evaluation category within the critical assessment of functional annotation. For further analysis of the functional characterization of Mfps, the probability range set to be above 0.800.

### Chemical structural characterization of Mfps

SAPS^32^ (https://www.ebi.ac.uk/Tools/seqstats/saps/) server evaluates a wide variety of protein sequence properties using statistics. Properties considered include compositional biases, clusters and runs of charge and other amino acid types, different kinds and extents of repetitive structures, locally periodic motifs, and anomalous spacing between identical residue types.

### Physio-chemical characterization of Mfps

Expasy protparam (https://web.expasy.org/protparam/) server^33^ analyze the physicochemical properties of Mfps likes, isoelectric point (pI), molecular weight (Mw), extinction coefficient (EC- quantitative study of protein-protein and protein-ligand interactions), instability index (II- stability of protein), aliphatic index (AI- relative volume of protein occupied by aliphatic side chains), and Grand Average of Hydropathicities (GRAVY – sum of all hydropathicity values of all amino acids divided by number of residues in a sequences).

### Accessible surface area (ASA) analysis

VADAR (http://vadar.wishartlab.com/) server^34^ is a compilation of more than 15 different algorithms and programs for analyzing and assessing peptide and protein structures from their PDB coordinate data.

### Ion ligand-binding site prediction

IonCom (https://zhanglab.ccmb.med.umich.edu/IonCom/)^35^ is a ligand-specific method for small ligand (including metal and acid radical ions) binding site prediction. Starting from given sequences or structures of the query proteins, IonCom performs a composite binding-site prediction that combines *ab intio* training and template-based transferals. The server focuses on binding site prediction of thirteen most important small ligand molecules, including nine metal ions (Zn^2+^, Cu^2+^, Fe^2+^, Fe^3+^, Ca^2+^, Mg^2+^, Mn^2+^, Na^+^, K^+^) and four acid radical ions (CO_3_^2−^, NO_2_^−^,SO_4_^2−^, PO_4_^3−^).

### Phylogeny construction of Mfps

Phylogenetic analysis of 78 Mfps were performed in MEGA X software^36^ and 78 Mfps were aligned by using MUSCLE software. The evolutionary history was inferred by using the Maximum Likelihood method and the JTT matrix-based model^37^. The tree with the highest log likelihood (−3861.65) and Initial tree(s) for the heuristic search was obtained automatically by applying Neighbor-Join and BioNJ algorithms to a matrix of pairwise distances estimated using a JTT model and then selecting the topology with superior log likelihood value. The tree is drawn to scale, with branch lengths measured in the number of substitutions per site.

### Ancestral analysis – mitogenome based

Ancestral states were inferred using the Maximum Likelihood method^38^ and the Tamura-Nei model^39^. The tree shows a set of possible nucleotides (states) at each ancestral node based on their inferred likelihood at site 1. For each node, only the most probable state is shown. The initial tree was inferred using the method. The rates among sites were treated as being uniform among sites (Uniform rates option). This analysis involved ten nucleotide sequences. Codon positions included were 1^st^ + 2^nd^ + 3^rd^ + Noncoding. Evolutionary analyses were conducted in MEGA X^36^ with MUSCLE alignment.

### TimeTree construction

Evolutionarily time scale tree construction of different orders of Bivalvia, Mytilidae genus, *Dreissena*, *Mytilus* and *Perna* species by using TimeTree^40^ (http://www.timetree.org/search/goto_timetree). The TimeTree is a public knowledge-base for information on the evolutionary timescale of life. In the TimeTree server build the time tree of a group of species or custom list^41–51^.

### OBIS map construction

Geographical distribution of selected Bivalvia species map constructed by using OBIS 2.0 server (https://mapper.obis.org/). Ocean biogeographical information system (OBIS) is a global open-access data and information clearing-house on marine biodiversity for science, conservation and sustainable development.

## Supplementary information

Dataset 1.

## Data Availability

The datasets generated during and/or analyzed during the current study are available from the corresponding author on reasonable request.
