# Supplementary material for: Computational modelling of wet adhesive mussel foot proteins (Bivalvia): Insights into the evolutionary convolution in diverse perspectives
Source: Sci Rep. 2020 Feb 13;10:2612. doi: 10.1038/s41598-020-59169-y (PMC7018726; doi:10.1038/s41598-020-59169-y)
Supplement: Supplementary file 1 — Dataset 1. [file 41598_2020_59169_MOESM1_ESM.docx]

Computational modelling of wet adhesive mussel foot proteins (Bivalvia): Insights into the evolutionary convolution in diverse perspectives

P. P. Anand^1^ & Y. Shibu Vardhanan^1^

^1^Toxicology & Biochemistry Division, Department of Zoology, University of Calicut, Kerala,

India 673 635

**Section 1 (S1): Geographical distribution**

Table 1: Geographical distribution frequency of selected bivalve species (Data retried from Catalogue of Life:2019 Annual Checklist (<http://www.catalogueoflife.org/annual-checklist/2019)> and in World Register of Marine Species (WORMS) (http://www.marinespecies. org/index.php) with updated current distribution from southern India.

| **Sl. No** | **Name** | **Systematic position** | **Distributions** | **Environment** |
| --- | --- | --- | --- | --- |
| 1 | Atrina pectinata (Linnaeus, 1767) | Ostreida: Pinnidae | Mediterranean Sea – Eastern Basin, North Atlantic Ocean | Marine |
| 2 | Dreissena polymorpha (Pallas, 1771) | Myida: Dreissenidae | Atlantic Europe, Baltic sea, Belarus, Canadian Exclusive Economic Zone, Celtic Sea, Elbe, English Channel, Germany, Gulf of Finland, Italy, North Atlantic Ocean, North Sea, Spain, Spanish Exclusive Economic Zone, Sweden, Swedish Exclusive Economic Zone, United Kingdom, United Kingdom Exclusive Economic Zone, United States Exclusive Economic Zone | Brackish, Freshwater |
| 3 | Mytilus californianus Conrad,1837 | Mytilida: Mytilidae | North Atlantic Ocean | Marine |
| 4 | Mytilus edulis Linnaeus, 1758 | Mytilida: Mytilidae | Adriatic Sea, Arctic Ocean, Baltic Sea, Bay of Fundy, Belgian Exclusive Economic Zone, Black Sea, Brandy Cove, Canadian Exclusive Economic Zone, De Haan, De Panne, Deadmans Harbour, Dorset, Gulf of Saint Lawrence; Mediterranean Sea, Mediterranean Sea - Eastern Basin, Muuga harbour (Port of Tallinn, Gulf of Finland), North Atlantic Ocean, North Sea, Oostduinkerke, Oostende, Overlapping claim Ukrainian Exclusive Economic Zone, Saguenay Fjord, Swedish Exclusive Economic Zone, Tanzania, West Coast of Norway, Wimereux, Zeebrugge | Marine |
| 5 | Mytilus galloprovincialis Lamarck,1819 | Mytilida: Mytilidae | Adriatic Sea, Alboran Sea, Asilah, Bizerte Lagoon, Black Sea, California; Celtic Sea, Dakhla, De Haan, East London, Elkhorn Slough, Essaouira, Ghar El Melh lagoon, Ksar-Sghir, Langebaan Lagoon, Mediterranean Sea, Mediterranean Sea - Eastern Basin, Mediterranean Sea - Western Basin, Mirhleft, Moroccan Exclusive Economic Zone, Nador, Namibian Exclusive Economic Zone, New Zealand Exclusive Economic Zone, North Atlantic Ocean, North Coast of Tunisia, North Pacific Ocean, North Sea, Plage Guy ville, Russian Exclusive economic Zone, South Africa (country), South Atlantic Ocean, South Coast of Africa, South Coast of England, South Wales, South West Coast of Apulia, Taghazout, Tan-tan, Tunis Lagoon, United States Exclusive Economic Zone, West Coast of England, West Coast of South Africa, Wimereux, Zemmour and South India | Marine |
| 6 | Mytilus unguiculatus Valenciennes, 1858 | Mytilida: Mytilidae | Correct distribution Unknown,  Western Pacific Ocean | Marine |
| 7 | Mizuhopecten yessoensis (Jay, 1857) | Pectinida:Pectinidae | North Pacific Ocean | Marine |
| 8 | Perna canaliculus (Gmelin, 1792) | Mytilida: Mytilidae | New Zealand Exclusive economic zone, South India | Marine |
| 9 | Perna viridis (Linnaeus, 1758) | Mytilida: Mytilidae | Australian Exclusive Economic Zone, Caribbean Sea, Cayman Islands, Colombia, East London, Florida, Indian Ocean, Kenyan Exclusive Economic Zone, Mombasa, North Atlantic Ocean, South Pacific, South Pacific Ocean, Trinidad and Tobago, Venezuela | Marine |

**Section 2 (S2): PDB id of Mfps**

Table 2: selected Mfps and their PDB id, for the access check <http://www.ebi.ac.uk/thornton-srv/databases/cgi-bin/pdbsum/GetPage.pl?pdbcode=index.html>

| **Sl. No** | **Protein** | **Variant** | **PDB ID** | **Password** |
| --- | --- | --- | --- | --- |
| 1 | Atrina pectinata | Fp1 | P183 | 082440 |
| 2 | Dreissena polymorpha | Fp1 | P189 | 104715 |
| 3 | Mytilus californianus | Fp1v1 | P194 | 103554 |
|  |  | Fp1v2 | P204 | 143541 |
|  |  | FP2 | P214 | 150245 |
|  |  | Fp3 v1 | P216 | 161705 |
|  |  | Fp3 v2 | P217 | 162821 |
|  |  | Fp3 v3 | P218 | 173505 |
|  |  | Fp3 v4 | P220 | 171020 |
|  |  | Fp3 v5 | P222 | 173830 |
|  |  | Fp3 v6 | P243 | 053638 |
|  |  | Fp3 v7 | P244 | 053251 |
|  |  | Fp3 v8 | P245 | 061208 |
|  |  | Fp3 v9 | P246 | 063421 |
|  |  | Fp3 v10 | P247 | 062441 |
|  |  | Fp3 v11 | P248 | 063658 |
|  |  | Fp4 v1 | P249 | 072811 |
|  |  | Fp4 v2 | P250 | 073130 |
|  |  | Fp5 | P252 | 070944 |
|  |  | Fp6 v1 | P253 | 074856 |
|  |  | Fp6 v2 | P254 | 085704 |
|  |  | Fp6 v3 | P273 | 082948 |
|  |  | Fp7 v1 | P423 | 102040 |
|  |  | Fp7 v2 | P431 | 111203 |
|  |  | Fp8 | P470 | 193228 |
|  |  | Fp9 v1 | P526 | 104950 |
|  |  | Fp9 v2 | P528 | 111109 |
|  |  | Fp10 | P534 | 113027 |
|  |  | Fp11 | P538 | 113443 |
|  |  | Fp12 | P721 | 075137 |
|  |  | Fp13 | P541 | 115155 |
|  |  | Fp14 | P543 | 120905 |
|  |  | Fp15 | P547 | 125817 |
|  |  | Fp16 | P726 | 083805 |
|  |  | Fp17 | P728 | 082816 |
|  |  | Fp18 | P729 | 085428 |
| 4 | Mytilus edulis | Fp1 | P733 | 093417 |
|  |  | Fp2 | P734 | 090826 |
| 5 | Mytilus galloprovincialis | Fp1 | P740 | 090057 |
|  |  | Fp3 v1 | P741 | 100819 |
|  |  | Fp3 v2 | P742 | 105126 |
| 6 | Mytilus unguiculatus | Fp2 | P743 | 101951 |
|  |  | Fp3 | P744 | 114806 |
|  |  | Fp3 v1 | P745 | 115912 |
|  |  | Fp3 v2 | P746 | 113222 |
|  |  | Fp3 v3 | P747 | 112033 |
|  |  | Fp3 v4 | P748 | 110945 |
|  |  | Fp3 v5 | P750 | 124901 |
|  |  | Fp3 v6 | P751 | 124412 |
|  |  | Fp3 v7 | P754 | 122448 |
|  |  | Fp3 v8 | P755 | 124355 |
|  |  | Fp3 v9 | P756 | 132404 |
|  |  | Fp3 v10 | P757 | 134717 |
|  |  | Fp3 v11 | P758 | 134524 |
|  |  | Fp3 v12 | P766 | 144014 |
|  |  | Fp3 v13 | P767 | 144024 |
|  |  | Fp3 v14 | P769 | 141333 |
|  |  | Fp6 | P770 | 141742 |
|  |  | Fp6 v1 | P772 | 140955 |
|  |  | Fp6 v2 | P775 | 150806 |
|  |  | Fp6 v3 | P777 | 154719 |
|  |  | Fp6 v4 | P778 | 151823 |
|  |  | Fp6 v5 | P802 | 164438 |
|  |  | Fp6 v6 | P804 | 165948 |
|  |  | Fp6 v7 | P805 | 164853 |
|  |  | Fp6 v8 | P817 | 173010 |
|  |  | Fp6 v9 | P820 | 170327 |
| 7 | Mizuhopecten yessoensis | Fp1 v1 | P822 | 172938 |
|  |  | Fp1 v2 | P826 | 174448 |
| 8 | Perna canaliculus | Fp1 v1 | P831 | 182009 |
|  |  | Fp1 v2 | P837 | 184226 |
|  |  | Fp1 v3 | P843 | 193133 |
|  |  | Fp1 v4 | P860 | 034113 |
| 9 | Perna viridis | Fp1 v1 | J824 | 114853 |
|  |  | Fp1 v2 | J825 | 113454 |
|  |  | Fp3 | J826 | 110355 |
|  |  | Fp5 | J827 | 112955 |
|  |  | Fp6 | J828 | 115355 |

**Section 3 (S3): Functional characterization of Mfps**

**Table 3: Biological process prediction of Mussel foot proteins (Mfps)**

| **Sl. No** | **Bivalve** | **Variant** | **Biological Process Prediction** | | |
| --- | --- | --- | --- | --- | --- |
|  |  |  | **GO term** | **Name** | **Probability** |
| 1 | *Mytilus californianus* | Mcfp1 v1 | GO:0007166 | Cell surface receptor signaling pathway | 0.866 |
|  |  |  | GO: 0019222 | Regulation of metabolic process | 0.843 |
| 2 |  | Mcfp1 v2 | GO:0007166 | Cell surface receptor signaling pathway | 0.886 |
|  |  |  | GO: 0019222 | Regulation of metabolic process | 0.882 |
| 3 |  | Mcfp2 | GO:0007186 | G-protein coupled receptor signaling pathway | 0.923 |
|  |  |  | GO:0072376 | Protein activation cascade | 0.922 |
|  |  |  | GO:0007166 | Cell surface receptor signaling pathway | 0.916 |
|  |  |  | GO:0019222 | Regulation of metabolic process | 0.906 |
|  |  |  | GO:0006810 | Transport | 0.840 |
| 4 |  | Mcfp3 v1 | GO:0007186 | G-protein coupled receptor signaling pathway | 0.984 |
|  |  |  | GO:0007166 | Cell surface receptor signaling pathway | 0.945 |
|  |  |  | GO:0006873 | Cellular ion homeostasis | 0.831 |
| 5 |  | Mcfp3 v2 | GO:0007186 | G-protein coupled receptor signaling pathway | 0.993 |
|  |  |  | GO:0007166 | Cell surface receptor signaling pathway | 0.960 |
|  |  |  | GO:0070613 | Regulation of protein processing | 0.897 |
|  |  |  | GO:0019222 | Regulation of metabolic process | 0.877 |
|  |  |  | GO:0010951 | Negative regulation of endopeptidase activity | 0.850 |
| 6 |  | Mcfp3 v3 | GO:0019222 | Regulation of metabolic process | 0.964 |
|  |  |  | GO:0007186 | G-protein coupled receptor signaling pathway | 0.891 |
|  |  |  | GO:0007166 | Cell surface receptor signaling pathway | 0.873 |
|  |  |  | GO:0006810 | Transport | 0.832 |
| 7 |  | Mcfp3 v4 | GO:0019222 | Regulation of metabolic process | 0.964 |
|  |  |  | GO:0007186 | G-protein coupled receptor signaling pathway | 0.889 |
|  |  |  | GO:0007166 | Cell surface receptor signaling pathway | 0.876 |
|  |  |  | GO:0006810 | Transport | 0.832 |
| 8 |  | Mcfp3 v5 | GO:0019222 | Regulation of metabolic process | 0.965 |
|  |  |  | GO:0007186 | G-protein coupled receptor signaling pathway | 0.889 |
|  |  |  | GO:0007166 | Cell surface receptor signaling pathway | 0.876 |
|  |  |  | GO:0006810 | Transport | 0.832 |
| 9 |  | Mcfp3 v6 | GO:0007186 | G-protein coupled receptor signaling pathway | 0.994 |
|  |  |  | GO:0007166 | Cell surface receptor signaling pathway | 0.953 |
|  |  |  | GO:0070613 | Regulation of protein processing | 0.903 |
|  |  |  | GO:0019222 | Regulation of metabolic process | 0.882 |
|  |  |  | GO:0006952 | Defense response | 0.808 |
| 10 |  | Mcfp3 v7 | GO:0019222 | Regulation of metabolic process | 0.951 |
|  |  |  | GO:0007166 | Cell surface receptor signaling pathway | 0.836 |
|  |  |  | GO:0006810 | Transport | 0.825 |
|  |  |  | GO:0007186 | G-protein coupled receptor signaling pathway | 0.821 |
| 11 |  | Mcfp3 v8 | GO:0019222 | Regulation of metabolic process | 0.971 |
|  |  |  | GO:0007166 | Cell surface receptor signaling pathway | 0.859 |
|  |  |  | GO:0007186 | G-protein coupled receptor signaling pathway | 0.850 |
|  |  |  | GO:0006810 | Transport | 0.825 |
|  |  |  | GO:0006811 | Ion transport | 0.800 |
| 12 |  | Mcfp3 v9 | GO:0019222 | Regulation of metabolic process | 0.954 |
|  |  |  | GO:0006810 | Transport | 0.828 |
|  |  |  | GO:0007166 | Cell surface receptor signaling pathway | 0.826 |
| 13 |  | Mcfp3 v10 | GO:0019222 | Regulation of metabolic process | 0.956 |
|  |  |  | GO:0007166 | Cell surface receptor signaling pathway | 0.829 |
|  |  |  | GO:0006810 | Transport | 0.828 |
|  |  |  | GO:0007186 | G-protein coupled receptor signaling pathway | 0.802 |
| 14 |  | Mcfp3 v11 | GO:0019222 | Regulation of metabolic process | 0.950 |
|  |  |  | GO:0006810 | Transport | 0.828 |
|  |  |  | GO:0007166 | Cell surface receptor signaling pathway | 0.825 |
| 15 |  | Mcfp4 v1 | GO:0019222 | Regulation of metabolic process | 0.901 |
|  |  |  | GO:0034645 | Cellular macromolecule biosynthesis process | 0.818 |
|  |  |  | GO:0006810 | Transport | 0.809 |
| 16 |  | Mcfp4 v2 | GO:0034645 | Cellular macromolecule biosynthesis process | 0.789 |
| 17 |  | Mcfp5 | GO:0019222 | Regulation of metabolic process | 0.932 |
|  |  |  | GO:0006810 | Transport | 0.812 |
| 18 |  | Mcfp6 v1 | GO:0007166 | Cell surface receptor signaling pathway | 0.884 |
|  |  |  | GO:0006810 | Transport | 0.825 |
| 19 |  | Mcfp6 v2 | GO:0007166 | Cell surface receptor signaling pathway | 0.873 |
|  |  |  | GO:0006810 | Transport | 0.848 |
| 20 |  | Mcfp6 v3 | GO:0007166 | Cell surface receptor signaling pathway | 0.876 |
|  |  |  | GO:0006810 | Transport | 0.837 |
| 21 |  | Mcfp7 v1 | GO:0019222 | Regulation of metabolic process | 0.978 |
|  |  |  | GO:0007166 | Cell surface receptor signaling pathway | 0.844 |
|  |  |  | GO:0006810 | Transport | 0.815 |
| 22 |  | Mcfp7 v2 | GO:0019222 | Regulation of metabolic process | 0.924 |
|  |  |  | GO:0007166 | Cell surface receptor signaling pathway | 0.851 |
|  |  |  | GO:0006810 | Transport | 0.800 |
| 23 |  | Mcfp8 | GO:0019222 | Regulation of metabolic process | 0.938 |
|  |  |  | GO:0034645 | Cellular macromolecule biosynthesis process | 0.835 |
|  |  |  | GO:0006810 | Transport | 0.823 |
| 24 |  | Mcfp9 v1 | GO:0006811 | Ion transport | 0.859 |
| 25 |  | Mcfp9 v2 | GO:0019222 | Regulation of metabolic process | 0.888 |
|  |  |  | GO:0006811 | Ion transport | 0.819 |
| 26 |  | Mcfp10 | GO:0006810 | Transport | 0.711 |
| 27 |  | Mcfp11 | GO:0034645 | Cellular macromolecule biosynthesis process | 0.771 |
| 28 |  | Mcfp12 | GO:0034645 | Cellular macromolecule biosynthesis process | 0.761 |
| 29 |  | Mcfp13 | GO:0034645 | Cellular macromolecule biosynthesis process | 0.796 |
| 30 |  | Mcfp14 | GO:0007166 | Cell surface receptor signaling pathway | 0.955 |
|  |  |  | GO:0007186 | G-protein coupled receptor signaling pathway | 0.908 |
|  |  |  | GO:0019222 | Regulation of metabolic process | 0.887 |
| 31 |  | Mcfp15 | GO:0019222 | Regulation of metabolic process | 0.798 |
| 32 |  | Mcfp16 | GO:0007166 | Cell surface receptor signaling pathway | 0.932 |
|  |  |  | GO:0019222 | Regulation of metabolic process | 0.919 |
| 33 |  | Mcfp17 | GO:0019222 | Regulation of metabolic process | 0.945 |
|  |  |  | GO:0006810 | Transport | 0.806 |
|  |  |  | GO:0007166 | Cell surface receptor signaling pathway | 0.801 |
| 34 |  | Mcfp18 | GO:0007186 | G-protein coupled receptor signaling pathway | 0.942 |
|  |  |  | GO:0019222 | Regulation of metabolic process | 0.939 |
|  |  |  | GO:0007166 | Cell surface receptor signaling pathway | 0.877 |
| 35 | *Atrina pectinate* | Apfp1 | GO:0007166 | Cell surface receptor signaling pathway | 0.848 |
|  |  |  | GO:0019222 | Regulation of metabolic process | 0.836 |
|  |  |  | GO:0010468 | Regulation of gene expression | 0.810 |
| 36 | *Dreissena polymorpha* | Dpfp1 | GO:0019222 | Regulation of metabolic process | 0.925 |
|  |  |  | GO:0007166 | Cell surface receptor signaling pathway | 0.917 |
|  |  |  | GO:0010468 | Regulation of gene expression | 0.830 |
| 37 | *Mytilus edulis* | Mefp1 | GO:0007166 | Cell surface receptor signaling pathway | 0.844 |
| 38 |  | Mefp2 | GO:0007166 | Cell surface receptor signaling pathway | 0.868 |
|  |  |  | GO:0019222 | Regulation of metabolic process | 0.860 |
|  |  |  | GO:0006810 | Transport | 0.856 |
| 39 | *Mytilus galloprovincialis* | Mgfp1 | GO:0007166 | Cell surface receptor signaling pathway | 0.797 |
| 40 |  | Mgfp3 v1 | GO:0007186 | G-protein coupled receptor signaling pathway | 0.989 |
|  |  |  | GO:0007166 | Cell surface receptor signaling pathway | 0.954 |
|  |  |  | GO:0019222 | Regulation of metabolic process | 0.885 |
|  |  |  | GO:0070613 | Regulation of protein processing | 0.822 |
| 41 |  | Mgfp3 v2 | GO:0007186 | G-protein coupled receptor signaling pathway | 0.992 |
|  |  |  | GO:0007166 | Cell surface receptor signaling pathway | 0.958 |
|  |  |  | GO:0070613 | Regulation of protein processing | 0.924 |
|  |  |  | GO:0019222 | Regulation of metabolic process | 0.873 |
|  |  |  | GO:0010951 | Negative regulation of endopeptidase activity | 0.841 |
|  |  |  | GO:0045861 | Negative regulation of proteolysis | 0.832 |
|  |  |  | GO:0006952 | Defense response | 0.815 |
| 42 | *Mytilus unguiculatus* | Mufp2 | GO:0007186 | G-protein coupled receptor signaling pathway | 0.912 |
|  |  |  | GO:0007166 | Cell surface receptor signaling pathway | 0.899 |
|  |  |  | GO:0019222 | Regulation of metabolic process | 0.896 |
|  |  |  | GO:0070613 | Regulation of protein processing | 0.810 |
|  |  |  | GO:0006810 | Transport | 0.808 |
| 43 |  | Mufp3 | GO:0019222 | Regulation of metabolic process | 0.983 |
|  |  |  | GO:0007186 | G-protein coupled receptor signaling pathway | 0.931 |
|  |  |  | GO:0007166 | Cell surface receptor signaling pathway | 0.894 |
|  |  |  | GO:0006810 | Transport | 0.836 |
| 44 |  | Mufp3 v1 | GO:0019222 | Regulation of metabolic process | 0.984 |
|  |  |  | GO:0007186 | G-protein coupled receptor signaling pathway | 0.920 |
|  |  |  | GO:0007166 | Cell surface receptor signaling pathway | 0.890 |
|  |  |  | GO:0006810 | Transport | 0.834 |
| 45 |  | Mufp3 v2 | GO:0019222 | Regulation of metabolic process | 0.986 |
|  |  |  | GO:0007186 | G-protein coupled receptor signaling pathway | 0.919 |
|  |  |  | GO:0007166 | Cell surface receptor signaling pathway | 0.891 |
|  |  |  | GO:0006810 | Transport | 0.834 |
| 46 |  | Mufp3 v3 | GO:0007186 | G-protein coupled receptor signaling pathway | 0.980 |
|  |  |  | GO:0007166 | Cell surface receptor signaling pathway | 0.949 |
|  |  |  | GO:0019222 | Regulation of metabolic process | 0.835 |
|  |  |  | GO:0006873 | Cellular ion homeostasis | 0.810 |
| 47 |  | Mufp3 v4 | GO:0019222 | Regulation of metabolic process | 0.973 |
|  |  |  | GO:0007186 | G-protein coupled receptor signaling pathway | 0.885 |
|  |  |  | GO:0007166 | Cell surface receptor signaling pathway | 0.879 |
|  |  |  | GO:0006810 | Transport | 0.834 |
| 48 |  | Mufp3 v5 | GO:0007186 | G-protein coupled receptor signaling pathway | 0.993 |
|  |  |  | GO:0007166 | Cell surface receptor signaling pathway | 0.955 |
|  |  |  | GO:0019222 | Regulation of metabolic process | 0.932 |
|  |  |  | GO:0070613 | Regulation of protein processing | 0.904 |
|  |  |  | GO:0045861 | Negative regulation of proteolysis | 0.812 |
|  |  |  | GO:0006952 | Defense response | 0.800 |
| 49 |  | Mufp3 v6 | GO:0007186 | G-protein coupled receptor signaling pathway | 0.993 |
|  |  |  | GO:0007166 | Cell surface receptor signaling pathway | 0.957 |
|  |  |  | GO:0019222 | Regulation of metabolic process | 0.933 |
|  |  |  | GO:0070613 | Regulation of protein processing | 0.897 |
|  |  |  | GO:0006952 | Defense response | 0.812 |
|  |  |  | GO:0045861 | Negative regulation of proteolysis | 0.800 |
| 50 |  | Mufp3 v7 | GO:0019222 | Regulation of metabolic process | 0.972 |
|  |  |  | GO:0007166 | Cell surface receptor signaling pathway | 0.875 |
|  |  |  | GO:0007186 | G-protein coupled receptor signaling pathway | 0.873 |
|  |  |  | GO:0006810 | Transport | 0.832 |
| 51 |  | Mufp3 v8 | GO:0019222 | Regulation of metabolic process | 0.983 |
|  |  |  | GO:0007186 | G-protein coupled receptor signaling pathway | 0.920 |
|  |  |  | GO:0007166 | Cell surface receptor signaling pathway | 0.894 |
|  |  |  | GO:0006810 | Transport | 0.834 |
| 52 |  | Mufp3 v9 | GO:0019222 | Regulation of metabolic process | 0.969 |
|  |  |  | GO:0007166 | Cell surface receptor signaling pathway | 0.872 |
|  |  |  | GO:0007186 | G-protein coupled receptor signaling pathway | 0.864 |
|  |  |  | GO:0006810 | Transport | 0.832 |
| 53 |  | Mufp3 v10 | GO:0007186 | G-protein coupled receptor signaling pathway | 0.993 |
|  |  |  | GO:0007166 | Cell surface receptor signaling pathway | 0.955 |
|  |  |  | GO:0019222 | Regulation of metabolic process | 0.919 |
|  |  |  | GO:0070613 | Regulation of protein processing | 0.905 |
|  |  |  | GO:0045861 | Negative regulation of proteolysis | 0.811 |
|  |  |  | GO:0010951 | Negative regulation of endopeptidase activity | 0.808 |
| 54 |  | Mufp3 v11 | GO:0007186 | G-protein coupled receptor signaling pathway | 0.966 |
|  |  |  | GO:0007166 | Cell surface receptor signaling pathway | 0.931 |
|  |  |  | GO:0019222 | Regulation of metabolic process | 0.863 |
|  |  |  | GO:0070613 | Regulation of protein processing | 0.857 |
| 55 |  | Mufp3 v12 | GO:0019222 | Regulation of metabolic process | 0.971 |
|  |  |  | GO:0007166 | Cell surface receptor signaling pathway | 0.875 |
|  |  |  | GO:0007186 | G-protein coupled receptor signaling pathway | 0.873 |
|  |  |  | GO:0006810 | Transport | 0.832 |
| 56 |  | Mufp3 v13 | GO:0007186 | G-protein coupled receptor signaling pathway | 0.983 |
|  |  |  | GO:0007166 | Cell surface receptor signaling pathway | 0.931 |
|  |  |  | GO:0070613 | Regulation of protein processing | 0.912 |
|  |  |  | GO:0019222 | Regulation of metabolic process | 0.887 |
|  |  |  | GO:0010951 | Negative regulation of endopeptidase activity | 0.858 |
|  |  |  | GO:0045861 | Negative regulation of proteolysis | 0.807 |
|  |  |  | GO:0044281 | Small molecule metabolic process | 0.806 |
| 57 |  | Mufp3 v14 | GO:0007186 | G-protein coupled receptor signaling pathway | 0.984 |
|  |  |  | GO:0007166 | Cell surface receptor signaling pathway | 0.947 |
|  |  |  | GO:0070613 | Regulation of protein processing | 0.849 |
|  |  |  | GO:0006873 | Cellular ion homeostasis | 0.827 |
|  |  |  | GO:0019222 | Regulation of metabolic process | 0.800 |
| 58 |  | Mufp6 | GO:0019222 | Regulation of metabolic process | 0.871 |
|  |  |  | GO:0006810 | Transport | 0.868 |
|  |  |  | GO:0007166 | Cell surface receptor signaling pathway | 0.863 |
| 59 |  | Mufp6 v1 | GO:0007166 | Cell surface receptor signaling pathway | 0.891 |
|  |  |  | GO:0006810 | Transport | 0.876 |
|  |  |  | GO:0019222 | Regulation of metabolic process | 0.865 |
|  |  |  | GO:0006955 | Immune response | 0.817 |
| 60 |  | Mufp6 v2 | GO:0007166 | Cell surface receptor signaling pathway | 0.896 |
|  |  |  | GO:0019222 | Regulation of metabolic process | 0.871 |
|  |  |  | GO:0006810 | Transport | 0.869 |
|  |  |  | GO:0006955 | Immune response | 0.836 |
| 61 |  | Mufp6 v3 | GO:0007166 | Cell surface receptor signaling pathway | 0.891 |
|  |  |  | GO:0006810 | Transport | 0.883 |
|  |  |  | GO:0019222 | Regulation of metabolic process | 0.860 |
|  |  |  | GO:0006955 | Immune response | 0.819 |
| 62 |  | Mufp6 v4 | GO:0007166 | Cell surface receptor signaling pathway | 0.889 |
|  |  |  | GO:0006810 | Transport | 0.876 |
|  |  |  | GO:0019222 | Regulation of metabolic process | 0.862 |
|  |  |  | GO:0006955 | Immune response | 0.817 |
| 63 |  | Mufp6 v5 | GO:0007166 | Cell surface receptor signaling pathway | 0.879 |
|  |  |  | GO:0006810 | Transport | 0.869 |
|  |  |  | GO:0019222 | Regulation of metabolic process | 0.828 |
|  |  |  | GO:0006959 | Humoral immune response | 0.815 |
|  |  |  | GO:0006955 | Immune response | 0.811 |
| 64 |  | Mufp6 v6 | GO:0007166 | Cell surface receptor signaling pathway | 0.876 |
|  |  |  | GO:0006810 | Transport | 0.842 |
| 65 |  | Mufp6 v7 | GO:0019222 | Regulation of metabolic process | 0.923 |
|  |  |  | GO:0007166 | Cell surface receptor signaling pathway | 0.906 |
|  |  |  | GO:0006955 | Immune response | 0.835 |
|  |  |  | GO:0006810 | Transport | 0.822 |
| 66 |  | Mufp6 v8 | GO:0019222 | Regulation of metabolic process | 0.920 |
|  |  |  | GO:0007166 | Cell surface receptor signaling pathway | 0.909 |
|  |  |  | GO:0072376 | Protein activation cascade | 0.868 |
|  |  |  | GO:0006810 | Transport | 0.854 |
|  |  |  | GO:0006955 | Immune response | 0.852 |
|  |  |  | GO:0038094 | Fc-gamma receptor signaling pathway | 0.805 |
| 67 |  | Mufp6 v9 | GO:0006810 | Transport | 0.960 |
|  |  |  | GO:0019222 | Regulation of metabolic process | 0.950 |
|  |  |  | GO:0006520 | Cellular amino acid metabolic process | 0.948 |
|  |  |  | GO:0044281 | Small molecule metabolic process | 0927 |
|  |  |  | GO:0034645 | Cellular macromolecule biosynthesis process | 0.916 |
|  |  |  | GO:0055085 | Transmembrane transport | 0.915 |
|  |  |  | GO:0006082 | Organic acid metabolic process | 0.881 |
|  |  |  | GO:0055144 | Oxidation-reduction process | 0.879 |
|  |  |  | GO:0034220 | Ion transmembrane transport | 0.877 |
|  |  |  | GO:0009059 | Macromolecule biosynthetic process | 0.867 |
|  |  |  | GO:0006796 | Phosphate-containing compound metabolic process | 0.867 |
|  |  |  | GO:0010468 | Regulation of gene expression | 0.865 |
|  |  |  | GO:0007166 | Cell surface receptor signaling pathway | 0.856 |
|  |  |  | GO:0051252 | Regulation of RNA metabolic process | 0.849 |
|  |  |  | GO:0051171 | Regulation of nitrogen compound metabolic process | 0.846 |
|  |  |  | GO:1903506 | Regulation of nucleic acid -template transcription | 0.830 |
|  |  |  | GO:2001141 | Regulation of RNA biosynthesis process | 0.817 |
|  |  |  | GO:0006412 | Translation | 0.802 |
|  |  |  | GO:0006955 | Immune response | 0.800 |
| 68 | *Mizuhopecten yessoensis* | Myfp1 v1 | GO:0019222 | Regulation of metabolic process | 0.954 |
|  |  |  | GO:0010468 | Regulation of gene expression | 0.879 |
|  |  |  | GO:0051171 | Regulation of nitrogen compound metabolic process | 0.822 |
|  |  |  | GO:2001141 | Regulation of RNA biosynthesis process | 0.819 |
| 69 |  | Myfp1 v2 | GO:0010468 | Regulation of gene expression | 0.895 |
|  |  |  | GO:2001141 | Regulation of RNA biosynthesis process | 0.890 |
|  |  |  | GO:0051252 | Regulation of RNA metabolic process | 0.887 |
|  |  |  | GO:0019222 | Regulation of metabolic process | 0.878 |
|  |  |  | GO:1903506 | Regulation of nucleic acid-templated transcription | 0.873 |
|  |  |  | GO:0051171 | Regulation of nitrogen compound metabolic process | 0.868 |
|  |  |  | GO:0006355 | Regulation of transcription, DNA-templated | 0.858 |
|  |  |  | GO:0009059 | Macromolecule biosynthetic process | 0.811 |
| 70 | *Perna canaliculus* | Pcfp1 v1 | GO:0010468 | Regulation of gene expression | 0.895 |
|  |  |  | GO:2001141 | Regulation of RNA biosynthesis process | 0.890 |
|  |  |  | GO:0051252 | Regulation of RNA metabolic process | 0.887 |
|  |  |  | GO:0019222 | Regulation of metabolic process | 0.878 |
|  |  |  | GO:1903506 | Regulation of nucleic acid-templated transcription | 0.873 |
|  |  |  | GO:0051171 | Regulation of nitrogen compound metabolic process | 0.868 |
|  |  |  | GO:0006355 | Regulation of transcription, DNA-templated | 0.858 |
|  |  |  | GO:0009059 | Macromolecule biosynthetic process | 0.811 |
| 71 |  | Pcfp1 v2 | GO:0019222 | Regulation of metabolic process | 0.924 |
|  |  |  | GO:0043241 | Protein complex disassembly | 0.878 |
|  |  |  | GO:0006810 | Transport | 0.835 |
| 72 |  | Pcfp1 v3 | GO:0019222 | Regulation of metabolic process | 0.966 |
|  |  |  | GO:0043241 | Protein complex disassembly | 0.872 |
|  |  |  | GO:0007166 | Cell surface receptor signaling pathway | 0.863 |
|  |  |  | GO:0006810 | Transport | 0.855 |
|  |  |  | GO:0034645 | Cellular macromolecule biosynthetic pathway | 0.828 |
|  |  |  | GO:0072376 | Protein activation cascade | 0.826 |
| 73 |  | Pcfp1 v4 | GO:0019222 | Regulation of metabolic process | 0.930 |
|  |  |  | GO:0043241 | Protein complex disassembly | 0.860 |
|  |  |  | GO:0006810 | Transport | 0.842 |
|  |  |  | GO:0034645 | Cellular macromolecule biosynthetic pathway | 0.834 |
|  |  |  | GO:0072376 | Protein activation cascade | 0.826 |
| 74 | *Perna viridis* | Pvfp1 v1 | GO:0043241 | Protein complex disassembly | 0.879 |
|  |  |  | GO:0007166 | Cell surface receptor signaling pathway | 0.817 |
| 75 |  | Pvfp1 v2 | GO:0043241 | Protein complex disassembly | 0.874 |
| 76 |  | Pvfp3 | GO:0019222 | Regulation of metabolic process | 0.923 |
|  |  |  | GO:0010951 | Negative regulation of endopeptidase activity | 0.888 |
|  |  |  | GO:0045861 | Negative regulation of proteolysis | 0.848 |
|  |  |  | GO:0007166 | Cell surface receptor signaling pathway | 0.802 |
| 77 |  | Pvfp5 | GO:0072376 | Protein activation cascade | 0.974 |
|  |  |  | GO:0007186 | G-protein coupled receptor signaling pathway | 0.945 |
|  |  |  | GO:0007166 | Cell surface receptor signaling pathway | 0.937 |
|  |  |  | GO:0019222 | Regulation of metabolic process | 0.882 |
|  |  |  | GO:0006810 | Transport | 0.861 |
| 78 |  | Pvfp6 | GO:0019222 | Regulation of metabolic process | 0.934 |
|  |  |  | GO:0007186 | G-protein coupled receptor signaling pathway | 0.934 |
|  |  |  | GO:0007166 | Cell surface receptor signaling pathway | 0.917 |

**Table 4: Cellular component prediction of Mussel foot proteins (Mfps)**

| **Sl. No** | **Bivalve** | **Variant** | **Cellular component Prediction** | | |
| --- | --- | --- | --- | --- | --- |
|  |  |  | **GO term** | **Name** | **Probability** |
| 1 | *Mytilus californianus* | Mcfp1 v1 | GO:0005576 | Extracellular region | 0.841 |
| 2 |  | Mcfp1 v2 | GO:0005576 | Extracellular region | 0.858 |
| 3 |  | Mcfp2 | GO:0005576 | Extracellular region | 0.880 |
|  |  |  | GO:0070062 | Extracellular vesicular exosome | 0.876 |
|  |  |  | GO:0031012 | Extracellular matrix | 0.854 |
| 4 |  | Mcfp3 v1 | GO:0070062 | Extracellular vesicular exosome | 0.963 |
|  |  |  | GO:0006676 | Extracellular region | 0.944 |
|  |  |  | GO:0031224 | Intrinsic component of membrane | 0.852 |
|  |  |  | GO:0005578 | Proteinaceous extracellular matrix | 0.838 |
|  |  |  | GO:0031982 | Vesicle | 0.815 |
| 5 |  | Mcfp3 v2 | GO:0005576 | Extracellular region | 0.963 |
|  |  |  | GO:0005578 | Proteinaceous extracellular matrix | 0.937 |
|  |  |  | GO:0070062 | Extracellular vesicular exosome | 0.919 |
|  |  |  | GO:0031988 | Membrane-bounded vesicle | 0.919 |
|  |  |  | GO:0031224 | Intrinsic component of membrane | 0.807 |
|  |  |  | GO:0031982 | Vesicle | 0.802 |
|  |  |  | GO:0005615 | Extracellular space | 0.801 |
| 6 |  | Mcfp3 v3 | GO:0005576 | Extracellular region | 0.950 |
|  |  |  | GO:0070062 | Extracellular vesicular exosome | 0.948 |
|  |  |  | GO:0031224 | Intrinsic component of membrane | 0.923 |
|  |  |  | GO:0031988 | Membrane-bounded vesicle | 0.838 |
|  |  |  | GO:0031982 | Vesicle | 0.826 |
| 7 |  | Mcfp3 v4 | GO:0005576 | Extracellular region | 0.948 |
|  |  |  | GO:0070062 | Extracellular vesicular exosome | 0.947 |
|  |  |  | GO:0031224 | Intrinsic component of membrane | 0.912 |
|  |  |  | GO:0031988 | Membrane-bounded vesicle | 0.833 |
|  |  |  | GO:0031982 | Vesicle | 0.821 |
| 8 |  | Mcfp3 v5 | GO:0070062 | Extracellular vesicular exosome | 0.950 |
|  |  |  | GO:0005576 | Extracellular region | 0.947 |
|  |  |  | GO:0031224 | Intrinsic component of membrane | 0.898 |
|  |  |  | GO:0031988 | Membrane-bounded vesicle | 0.832 |
|  |  |  | GO:0031982 | Vesicle | 0.821 |
| 9 |  | Mcfp3 v6 | GO:0005576 | Extracellular region | 0.955 |
|  |  |  | GO:0005578 | Proteinaceous extracellular matrix | 0.932 |
|  |  |  | GO:0070062 | Extracellular vesicular exosome | 0.929 |
|  |  |  | GO:0031988 | Membrane-bounded vesicle | 0.858 |
|  |  |  | GO:0031012 | Extracellular matrix | 0.822 |
|  |  |  | GO:0031982 | Vesicle | 0.806 |
| 10 |  | Mcfp3 v7 | GO:0070062 | Extracellular vesicular exosome | 0.948 |
|  |  |  | GO:0005576 | Extracellular region | 0.944 |
|  |  |  | GO:0031224 | Intrinsic component of membrane | 0.911 |
|  |  |  | GO:0031982 | Vesicle | 0.823 |
|  |  |  | GO:0031988 | Membrane-bounded vesicle | 0.817 |
| 11 |  | Mcfp3 v8 | GO:0005576 | Extracellular region | 0.960 |
|  |  |  | GO:0070062 | Extracellular vesicular exosome | 0.911 |
|  |  |  | GO:0031224 | Intrinsic component of membrane | 0.854 |
|  |  |  | GO:0031988 | Membrane-bounded vesicle | 0.840 |
|  |  |  | GO:0031982 | Vesicle | 0.812 |
| 12 |  | Mcfp3 v9 | GO:0070062 | Extracellular vesicular exosome | 0.956 |
|  |  |  | GO:0031224 | Intrinsic component of membrane | 0.945 |
|  |  |  | GO:0005576 | Extracellular region | 0.937 |
|  |  |  | GO:0016021 | Integral component of membrane | 0.832 |
|  |  |  | GO:0031982 | Vesicle | 0.819 |
|  |  |  | GO:0016020 | Membrane | 0.809 |
| 13 |  | Mcfp3 v10 | GO:0031224 | Intrinsic component of membrane | 0.960 |
|  |  |  | GO:0070062 | Extracellular vesicular exosome | 0.950 |
|  |  |  | GO:0005576 | Extracellular region | 0.936 |
|  |  |  | GO:0016021 | Integral component of membrane | 0.860 |
|  |  |  | GO:0031982 | Vesicle | 0.818 |
|  |  |  | GO:0016020 | Membrane | 0.809 |
| 14 |  | Mcfp3 v11 | GO:0070062 | Extracellular vesicular exosome | 0.958 |
|  |  |  | GO:0031224 | Intrinsic component of membrane | 0.948 |
|  |  |  | GO:0005576 | Extracellular region | 0.938 |
|  |  |  | GO:0016021 | Integral component of membrane | 0.847 |
|  |  |  | GO:0031982 | Vesicle | 0.820 |
|  |  |  | GO:0016020 | Membrane | 0.805 |
| 15 |  | Mcfp4 v1 | GO:0016020 | Membrane | 0.903 |
|  |  |  | GO:0070062 | Extracellular vesicular exosome | 0.828 |
| 16 |  | Mcfp4 v2 | GO:0005576 | Extracellular region | 0.850 |
| 17 |  | Mcfp5 | GO:0005576 | Extracellular region | 0.959 |
|  |  |  | GO:0070062 | Extracellular vesicular exosome | 0.927 |
|  |  |  | GO:0031982 | Vesicle | 0.821 |
| 18 |  | Mcfp6 v1 | GO:0005576 | Extracellular region | 0.948 |
|  |  |  | GO:0005615 | Extracellular space | 0.887 |
|  |  |  | GO:0070062 | Extracellular vesicular exosome | 0.883 |
|  |  |  | GO:0005578 | Proteinaceous extracellular matrix | 0.850 |
|  |  |  | GO:0031982 | Vesicle | 0.816 |
| 19 |  | Mcfp6 v2 | GO:0005576 | Extracellular region | 0.952 |
|  |  |  | GO:0070062 | Extracellular vesicular exosome | 0.910 |
|  |  |  | GO:0005615 | Extracellular space | 0.885 |
|  |  |  | GO:0031982 | Vesicle | 0.830 |
|  |  |  | GO:0005578 | Proteinaceous extracellular matrix | 0.814 |
|  |  |  | GO:0016020 | Membrane | 0.800 |
| 20 |  | Mcfp6 v3 | GO:0005576 | Extracellular region | 0.943 |
|  |  |  | GO:0070062 | Extracellular vesicular exosome | 0.892 |
|  |  |  | GO:0005615 | Extracellular space | 0.867 |
|  |  |  | GO:0005578 | Proteinaceous extracellular matrix | 0.822 |
|  |  |  | GO:0031982 | Vesicle | 0.814 |
| 21 |  | Mcfp7 v1 | GO:0070062 | Extracellular vesicular exosome | 0.860 |
|  |  |  | GO:0005576 | Extracellular region | 0.850 |
| 22 |  | Mcfp7 v2 | GO:0070062 | Extracellular vesicular exosome | 0.903 |
|  |  |  | GO:0005576 | Extracellular region | 0.882 |
| 23 |  | Mcfp8 | GO:0070062 | Extracellular vesicular exosome | 0.974 |
|  |  |  | GO:0016020 | Membrane | 0.923 |
|  |  |  | GO:0005576 | Extracellular region | 0.841 |
|  |  |  | GO:0005783 | Endoplasmic reticulum | 0.840 |
|  |  |  | GO:0005886 | Plasma membrane | 0.821 |
| 24 |  | Mcfp9 v1 | GO:0005576 | Extracellular region | 0.923 |
| 25 |  | Mcfp9 v2 | GO:0005576 | Extracellular region | 0.936 |
| 26 |  | Mcfp10 | GO:0005576 | Extracellular region | 0.923 |
|  |  |  | GO:0031988 | Membrane-bounded vesicle | 0.815 |
|  |  |  | GO:0070062 | Extracellular vesicular exosome | 0.809 |
| 27 |  | Mcfp11 | GO:0005576 | Extracellular region | 0.914 |
|  |  |  | GO:0031982 | Vesicle | 0.843 |
| 28 |  | Mcfp12 | GO:0005576 | Extracellular region | 0.867 |
|  |  |  | GO:0031982 | Vesicle | 0.808 |
| 29 |  | Mcfp13 | GO:0070062 | Extracellular vesicular exosome | 0.958 |
|  |  |  | GO:0005576 | Extracellular region | 0.929 |
|  |  |  | GO:0016020 | Membrane | 0.897 |
|  |  |  | GO:0031982 | Vesicle | 0.849 |
| 30 |  | Mcfp14 | GO:0005576 | Extracellular region | 0.932 |
|  |  |  | GO:0005615 | Extracellular space | 0.896 |
|  |  |  | GO:0070062 | Extracellular vesicular exosome | 0.893 |
| 31 |  | Mcfp15 | GO:0005576 | Extracellular region | 0.883 |
|  |  |  | GO:0016020 | Membrane | 0.822 |
| 32 |  | Mcfp16 | GO:0016020 | Membrane | 0.893 |
|  |  |  | GO:0005615 | Extracellular space | 0.843 |
|  |  |  | GO:0005576 | Extracellular region | 0.838 |
| 33 |  | Mcfp17 | GO:0005576 | Extracellular region | 0.915 |
|  |  |  | GO:0005615 | Extracellular space | 0.834 |
| 34 |  | Mcfp18 | GO:0005576 | Extracellular region | 0.940 |
|  |  |  | GO:0031982 | Vesicle | 0.842 |
| 35 | *Atrina pectinate* | Apfp1 | GO:0005615 | Extracellular space | 0.893 |
|  |  |  | GO:0005576 | Extracellular region | 0.881 |
|  |  |  | GO:0070062 | Extracellular vesicular exosome | 0.842 |
| 36 | *Dreissena polymorpha* | Dpfp1 | GO:0005576 | Extracellular region | 0.892 |
| 37 | *Mytilus edulis* | Mefp1 | GO:0005576 | Extracellular region | 0.824 |
|  |  |  | GO:0070062 | Extracellular vesicular exosome | 0.804 |
| 38 |  | Mefp2 | GO:0031012 | Extracellular matrix | 0.881 |
|  |  |  | GO:0005576 | Extracellular region | 0.860 |
|  |  |  | GO:0005578 | Proteinaceous extracellular matrix | 0.845 |
| 39 | *Mytilus galloprovincialis* | Mgfp1 | GO:0005788 | Endoplasmic reticulum lumen | 0.815 |
|  |  |  | GO:0070062 | Extracellular vesicular exosome | 0.803 |
| 40 |  | Mgfp3 v1 | GO:0005576 | Extracellular region | 0.957 |
|  |  |  | GO:0005578 | Proteinaceous extracellular matrix | 0.940 |
|  |  |  | GO:0070062 | Extracellular vesicular exosome | 0.934 |
|  |  |  | GO:0031988 | Membrane-bounded vesicle | 0.843 |
| 41 |  | Mgfp3 v2 | GO:0005576 | Extracellular region | 0.955 |
|  |  |  | GO:0070062 | Extracellular vesicular exosome | 0.941 |
|  |  |  | GO:0005578 | Proteinaceous extracellular matrix | 0.926 |
|  |  |  | GO:0031988 | Membrane-bounded vesicle | 0.852 |
|  |  |  | GO:0031224 | Intrinsic component of membrane | 0.820 |
|  |  |  | GO:0005615 | Extracellular space | 0.810 |
|  |  |  | GO:0031982 | Vesicle | 0.804 |
| 42 | *Mytilus unguiculatus* | Mufp2 | GO:0005576 | Extracellular region | 0.925 |
|  |  |  | GO:0070062 | Extracellular vesicular exosome | 0.870 |
|  |  |  | GO:0031012 | Extracellular matrix | 0.862 |
|  |  |  | GO:0005578 | Proteinaceous extracellular matrix | 0.856 |
|  |  |  | GO:0031988 | Membrane-bounded vesicle | 0.815 |
| 43 |  | Mufp3 | GO:0005576 | Extracellular region | 0.953 |
|  |  |  | GO:0070062 | Extracellular vesicular exosome | 0.922 |
|  |  |  | GO:0031224 | Intrinsic component membrane | 0.895 |
|  |  |  | GO:0031988 | Membrane-bounded vesicle | 0.857 |
|  |  |  | GO:0031012 | Extracellular matrix | 0.825 |
|  |  |  | GO:0031982 | Vesicle | 0.811 |
| 44 |  | Mufp3 v1 | GO:0005576 | Extracellular region | 0.954 |
|  |  |  | GO:0031224 | Intrinsic component membrane | 0.920 |
|  |  |  | GO:0070062 | Extracellular vesicular exosome | 0.914 |
|  |  |  | GO:0031988 | Membrane-bounded vesicle | 0.851 |
|  |  |  | GO:0031982 | Vesicle | 0.805 |
| 45 |  | Mufp3 v2 | GO:0005576 | Extracellular region | 0.952 |
|  |  |  | GO:0031224 | Intrinsic component membrane | 0.940 |
|  |  |  | GO:0070062 | Extracellular vesicular exosome | 0.909 |
|  |  |  | GO:0031988 | Membrane-bounded vesicle | 0.844 |
|  |  |  | GO:0031982 | Vesicle | 0.802 |
| 46 |  | Mufp3 v3 | GO:0070062 | Extracellular vesicular exosome | 0.959 |
|  |  |  | GO:0005576 | Extracellular region | 0.940 |
|  |  |  | GO:0005578 | Proteinaceous extracellular matrix | 0.844 |
|  |  |  | GO:0031224 | Intrinsic component of membrane | 0.820 |
|  |  |  | GO:0031982 | Vesicle | 0.808 |
| 47 |  | Mufp3 v4 | GO:0031224 | Intrinsic component of membrane | 0.954 |
|  |  |  | GO:0005576 | Extracellular region | 0.950 |
|  |  |  | GO:0070062 | Extracellular vesicular exosome | 0.946 |
|  |  |  | GO:0016021 | Integral component of membrane | 0.840 |
|  |  |  | GO:0031988 | Membrane-bounded vesicle | 0.839 |
|  |  |  | GO:0031982 | Vesicle | 0.821 |
| 48 |  | Mufp3 v5 | GO:0005576 | Extracellular region | 0.953 |
|  |  |  | GO:0031224 | Intrinsic component of membrane | 0.933 |
|  |  |  | GO:0005578 | Proteinaceous extracellular matrix | 0.924 |
|  |  |  | GO:0070062 | Extracellular vesicular exosome | 0.908 |
|  |  |  | GO:0031988 | Membrane-bounded vesicle | 0.852 |
|  |  |  | GO:0031982 | Vesicle | 0.805 |
| 49 |  | Mufp3 v6 | GO:0005576 | Extracellular region | 0.955 |
|  |  |  | GO:0005578 | Proteinaceous extracellular matrix | 0.934 |
|  |  |  | GO:0031224 | Intrinsic component of membrane | 0.907 |
|  |  |  | GO:0070062 | Extracellular vesicular exosome | 0.901 |
|  |  |  | GO:0031988 | Membrane-bounded vesicle | 0.856 |
|  |  |  | GO:0031982 | Vesicle | 0.807 |
| 50 |  | Mufp3 v7 | GO:0005576 | Extracellular region | 0.950 |
|  |  |  | GO:0070062 | Extracellular vesicular exosome | 0.945 |
|  |  |  | GO:0031224 | Intrinsic component of membrane | 0.938 |
|  |  |  | GO:0031988 | Membrane-bounded vesicle | 0.837 |
|  |  |  | GO:0031982 | Vesicle | 0.825 |
|  |  |  | GO:0016021 | Integral component of membrane | 0.816 |
| 51 |  | Mufp3 v8 | GO:0005576 | Extracellular region | 0.948 |
|  |  |  | GO:0070062 | Extracellular vesicular exosome | 0.933 |
|  |  |  | GO:0031988 | Membrane-bounded vesicle | 0.851 |
|  |  |  | GO:0031982 | Vesicle | 0.809 |
|  |  |  | GO:0031224 | Intrinsic component of membrane | 0.805 |
| 52 |  | Mufp3 v9 | GO:0070062 | Extracellular vesicular exosome | 0.950 |
|  |  |  | GO:0005576 | Extracellular region | 0.948 |
|  |  |  | GO:0031224 | Intrinsic component of membrane | 0.932 |
|  |  |  | GO:0031988 | Membrane-bounded vesicle | 0.829 |
|  |  |  | GO:0031982 | Vesicle | 0.825 |
|  |  |  | GO:0016021 | Integral component of membrane | 0.809 |
| 53 |  | Mufp3 v10 | GO:0005576 | Extracellular region | 0.952 |
|  |  |  | GO:0031224 | Intrinsic component of membrane | 0.919 |
|  |  |  | GO:0005578 | Proteinaceous extracellular matrix | 0.917 |
|  |  |  | GO:0070062 | Extracellular vesicular exosome | 0.903 |
|  |  |  | GO:0031988 | Membrane-bounded vesicle | 0.848 |
|  |  |  | GO:0031982 | Vesicle | 0.801 |
| 54 |  | Mufp3 v11 | GO:0005576 | Extracellular region | 0.950 |
|  |  |  | GO:0070062 | Extracellular vesicular exosome | 0.947 |
|  |  |  | GO:0031224 | Intrinsic component of membrane | 0.925 |
|  |  |  | GO:0005578 | Proteinaceous extracellular matrix | 0.884 |
|  |  |  | GO:0031988 | Membrane-bounded vesicle | 0.849 |
|  |  |  | GO:0005615 | Extracellular space | 0.819 |
|  |  |  | GO:0031982 | Vesicle | 0.816 |
| 55 |  | Mufp3 v12 | GO:0005576 | Extracellular region | 0.949 |
|  |  |  | GO:0070062 | Extracellular vesicular exosome | 0.948 |
|  |  |  | GO:0031224 | Intrinsic component of membrane | 0.943 |
|  |  |  | GO:0031988 | Membrane-bounded vesicle | 0.836 |
|  |  |  | GO:0031982 | Vesicle | 0.826 |
|  |  |  | GO:0016021 | Integral component of membrane | 0.822 |
| 56 |  | Mufp3 v13 | GO:0005576 | Extracellular region | 0.954 |
|  |  |  | GO:0070062 | Extracellular vesicular exosome | 0.941 |
|  |  |  | GO:0005578 | Proteinaceous extracellular matrix | 0.915 |
|  |  |  | GO:0031988 | Membrane-bounded vesicle | 0.849 |
|  |  |  | GO:0005615 | Extracellular space | 0.844 |
|  |  |  | GO:0031012 | Extracellular matrix | 0.838 |
|  |  |  | GO:0031224 | Intrinsic component of membrane | 0.813 |
|  |  |  | GO:0031982 | Vesicle | 0.800 |
| 57 |  | Mufp3 v14 | GO:0070062 | Extracellular vesicular exosome | 0.969 |
|  |  |  | GO:0005576 | Extracellular region | 0.950 |
|  |  |  | GO:0005578 | Proteinaceous extracellular matrix | 0.872 |
|  |  |  | GO:0031224 | Intrinsic component of membrane | 0.861 |
|  |  |  | GO:0031988 | Membrane-bounded vesicle | 0.831 |
|  |  |  | GO:0031982 | Vesicle | 0.816 |
| 58 |  | Mufp6 | GO:0005576 | Extracellular region | 0.939 |
|  |  |  | GO:0070062 | Extracellular vesicular exosome | 0.934 |
|  |  |  | GO:0005615 | Extracellular space | 0.892 |
|  |  |  | GO:0016020 | Membrane | 0.818 |
|  |  |  | GO:0031982 | Vesicle | 0.813 |
| 59 |  | Mufp6 v1 | GO:0005576 | Extracellular region | 0.949 |
|  |  |  | GO:0005615 | Extracellular space | 0.941 |
|  |  |  | GO:0070062 | Extracellular vesicular exosome | 0.928 |
|  |  |  | GO:0016020 | Membrane | 0.858 |
|  |  |  | GO:0031982 | Vesicle | 0.826 |
| 60 |  | Mufp6 v2 | GO:0005576 | Extracellular region | 0.954 |
|  |  |  | GO:0005615 | Extracellular space | 0.942 |
|  |  |  | GO:0070062 | Extracellular vesicular exosome | 0.931 |
|  |  |  | GO:0031982 | Vesicle | 0.831 |
|  |  |  | GO:0016020 | Membrane | 0.802 |
| 61 |  | Mufp6 v3 | GO:0005576 | Extracellular region | 0.949 |
|  |  |  | GO:0005615 | Extracellular space | 0.937 |
|  |  |  | GO:0070062 | Extracellular vesicular exosome | 0.933 |
|  |  |  | GO:0016020 | Membrane | 0.846 |
|  |  |  | GO:0031982 | Vesicle | 0.830 |
| 62 |  | Mufp6 v4 | GO:0005576 | Extracellular region | 0.950 |
|  |  |  | GO:0005615 | Extracellular space | 0.942 |
|  |  |  | GO:0070062 | Extracellular vesicular exosome | 0.928 |
|  |  |  | GO:0016020 | Membrane | 0.858 |
|  |  |  | GO:0031982 | Vesicle | 0.828 |
| 63 |  | Mufp6 v5 | GO:0005576 | Extracellular region | 0.949 |
|  |  |  | GO:0005615 | Extracellular space | 0.936 |
|  |  |  | GO:0070062 | Extracellular vesicular exosome | 0.912 |
|  |  |  | GO:0016020 | Membrane | 0.867 |
|  |  |  | GO:0031982 | Vesicle | 0.820 |
| 64 |  | Mufp6 v6 | GO:0005576 | Extracellular region | 0.951 |
|  |  |  | GO:0005615 | Extracellular space | 0.939 |
|  |  |  | GO:0070062 | Extracellular vesicular exosome | 0.891 |
|  |  |  | GO:0031982 | Vesicle | 0.821 |
| 65 |  | Mufp6 v7 | GO:0005576 | Extracellular region | 0.952 |
|  |  |  | GO:0005615 | Extracellular space | 0.941 |
|  |  |  | GO:0070062 | Extracellular vesicular exosome | 0.925 |
|  |  |  | GO:0005578 | Proteinaceous extracellular matrix | 0.818 |
|  |  |  | GO:0031982 | Vesicle | 0.818 |
|  |  |  | GO:0016020 | Membrane | 0.812 |
| 66 |  | Mufp6 v8 | GO:0005576 | Extracellular region | 0.949 |
|  |  |  | GO:0070062 | Extracellular vesicular exosome | 0.935 |
|  |  |  | GO:0005615 | Extracellular space | 0.926 |
|  |  |  | GO:0016020 | Membrane | 0.875 |
| 67 |  | Mufp6 v9 | GO:0005840 | Ribosome | 0.893 |
|  |  |  | GO:0070062 | Extracellular vesicular exosome | 0.890 |
|  |  |  | GO:0016020 | Membrane | 0.887 |
|  |  |  | GO:0005576 | Extracellular region | 0.884 |
| 68 | *Mizuhopecten yessoensis* | Myfp1 v1 | GO:0071944 | Cell periphery | 0.744 |
| 69 |  | Myfp1 v2 | GO:0031224 | Intrinsic component membrane | 0.741 |
| 70 | *Perna canaliculus* | Pcfp1 v1 | GO:0031224 | Intrinsic component membrane | 0.741 |
| 71 |  | Pcfp1 v2 | GO:0070062 | Extracellular vesicular exosome | 0.986 |
|  |  |  | GO:0016020 | Membrane | 0.895 |
|  |  |  | GO:0071944 | Cell periphery | 0.863 |
| 72 |  | Pcfp1 v3 | GO:0070062 | Extracellular vesicular exosome | 0.935 |
|  |  |  | GO:0016020 | Membrane | 0.921 |
|  |  |  | GO:0005886 | Plasma membrane | 0.820 |
| 73 |  | Pcfp1 v4 | GO:0016020 | Membrane | 0.915 |
|  |  |  | GO:0070062 | Extracellular vesicular exosome | 0.903 |
|  |  |  | GO:0005886 | Plasma membrane | 0.819 |
| 74 | *Perna viridis* | Pvfp1 v1 | GO:0005576 | Extracellular region | 0.875 |
|  |  |  | GO:0031224 | Intrinsic component membrane | 0.856 |
|  |  |  | GO:0070062 | Extracellular vesicular exosome | 0.852 |
| 75 |  | Pvfp1 v2 | GO:0005576 | Extracellular region | 0.884 |
|  |  |  | GO:0031224 | Intrinsic component membrane | 0.869 |
|  |  |  | GO:0070062 | Extracellular vesicular exosome | 0.849 |
| 76 |  | Pvfp3 | GO:0005576 | Extracellular region | 0.873 |
|  |  |  | GO:0016020 | Membrane | 0.836 |
| 77 |  | Pvfp5 | GO:0005576 | Extracellular region | 0.901 |
|  |  |  | GO:0031012 | Extracellular matrix | 0.870 |
|  |  |  | GO:0070062 | Extracellular vesicular exosome | 0.865 |
|  |  |  | GO:0005578 | Proteinaceous extracellular matrix | 0.863 |
|  |  |  | GO:0005615 | Extracellular space | 0.812 |
| 78 |  | Pvfp6 | GO:0005576 | Extracellular region | 0.956 |
|  |  |  | GO:0005578 | Proteinaceous extracellular matrix | 0.920 |
|  |  |  | GO:0070062 | Extracellular vesicular exosome | 0.884 |
|  |  |  | GO:0005615 | Extracellular space | 0.881 |
|  |  |  | GO:0031012 | Extracellular matrix | 0.875 |
|  |  |  | GO:0031982 | Membrane-bounded vesicle | 0.806 |
|  |  |  | GO:0031982 | Vesicle | 0.804 |

**Table 5: Molecular function prediction of Mussel foot proteins (Mfps)**

| **Sl. No** | **Bivalve** | **Variant** | **Molecular function Prediction** | | |
| --- | --- | --- | --- | --- | --- |
|  |  |  | **GO term** | **Name** | **Probability** |
| 1 | *Mytilus californianus* | Mcfp1 v1 | GO:0008083 | Growth factor activity | 0.870 |
| 2 |  | Mcfp1 v2 | GO:0008083 | Growth factor activity | 0.913 |
|  |  |  | GO:0044822 | Poly(A) RNA binding | 0.814 |
|  |  |  | GO:0003676 | Nucleic acid binding | 0.811 |
|  |  |  | GO:0004930 | G-protein coupled receptor activity | 0.801 |
| 3 |  | Mcfp2 | GO:0004872 | Receptor activity | 0.991 |
|  |  |  | GO:0004888 | Transmembrane signaling receptor activity | 0.986 |
|  |  |  | GO:0004930 | G-protein coupled receptor activity | 0.981 |
|  |  |  | GO:0005539 | Glycosaminoglycan binding | 0.961 |
|  |  |  | GO:0038023 | Signaling transducer activity | 0.951 |
|  |  |  | GO:0008083 | Growth factor activity | 0.932 |
|  |  |  | GO:0004175 | Endopeptidase activity | 0.880 |
|  |  |  | GO:0008270 | Zinc ion binding | 0.850 |
|  |  |  | GO:0044822 | Poly(A) RNA binding | 0.832 |
| 4 |  | Mcfp3 v1 | GO:0004930 | G-protein coupled receptor activity | 0.940 |
|  |  |  | GO:0008236 | Serine-type peptidase activity | 0.938 |
|  |  |  | GO:0004872 | Receptor activity | 0.916 |
|  |  |  | GO:0003735 | Structural constituent of ribosome | 0.914 |
|  |  |  | GO:0001664 | G-protein coupled receptor binding | 0.912 |
|  |  |  | GO:003014 | Peptidase inhibitor activity | 0.888 |
|  |  |  | GO:0038023 | Signaling receptor activity | 0.870 |
|  |  |  | GO:0008083 | Growth factor activity | 0.867 |
|  |  |  | GO:0004888 | Transmembrane signaling receptor activity | 0.857 |
|  |  |  | GO:0005126 | Cytokine receptor binding | 0.856 |
|  |  |  | GO:0005125 | Cytokine activity | 0.837 |
|  |  |  | GO:0005198 | Structural molecule activity | 0.813 |
| 5 |  | Mcfp3 v2 | GO:0030414 | Peptidase inhibitor activity | 0.972 |
|  |  |  | GO:0001664 | G-protein coupled receptor binding | 0.969 |
|  |  |  | GO:0004857 | Enzyme inhibitor activity | 0.963 |
|  |  |  | GO:0005125 | Cytokine activity | 0.962 |
|  |  |  | GO:0008083 | Growth factor activity | 0.953 |
|  |  |  | GO:0005126 | Cytokine receptor binding | 0.950 |
|  |  |  | GO:0004930 | G-protein coupled receptor activity | 0.913 |
|  |  |  | GO:0008236 | Serine-type peptidase activity | 0.887 |
|  |  |  | GO:0030234 | Enzyme regulatory activity | 0.820 |
|  |  |  | GO:0004872 | Receptor activity | 0.802 |
| 6 |  | Mcfp3 v3 | GO:0004872 | Receptor activity | 0.948 |
|  |  |  | GO:0030414 | Peptidase inhibitor activity | 0.943 |
|  |  |  | GO:0004930 | G-protein coupled receptor activity | 0.941 |
|  |  |  | GO:0004857 | Enzyme inhibitor activity | 0.862 |
|  |  |  | GO:0005198 | Structural molecule activity | 0.818 |
|  |  |  | GO:0003676 | Nucleic acid binding | 0.809 |
| 7 |  | Mcfp3 v4 | GO:0030414 | Peptidase inhibitor activity | 0.948 |
|  |  |  | GO:0004872 | Receptor activity | 0.943 |
|  |  |  | GO:0004930 | G-protein coupled receptor activity | 0.941 |
|  |  |  | GO:0004857 | Enzyme inhibitor activity | 0.869 |
|  |  |  | GO:0003676 | Nucleic acid binding | 0.811 |
| 8 |  | Mcfp3 v5 | GO:0030414 | Peptidase inhibitor activity | 0.948 |
|  |  |  | GO:0004872 | Receptor activity | 0.942 |
|  |  |  | GO:0004930 | G-protein coupled receptor activity | 0.941 |
|  |  |  | GO:0004857 | Enzyme inhibitor activity | 0.869 |
|  |  |  | GO:0003676 | Nucleic acid binding | 0.811 |
| 9 |  | Mcfp3 v6 | GO:0030414 | Peptidase inhibitor activity | 0.981 |
|  |  |  | GO:0001664 | G-protein coupled receptor binding | 0.965 |
|  |  |  | GO:0004857 | Enzyme inhibitor activity | 0.958 |
|  |  |  | GO:0005125 | Cytokine activity | 0.952 |
|  |  |  | GO:0005126 | Cytokine receptor binding | 0.951 |
|  |  |  | GO:0008083 | Growth factor activity | 0.938 |
|  |  |  | GO:0004930 | G-protein coupled receptor activity | 0.927 |
|  |  |  | GO:0004872 | Receptor activity | 0.895 |
|  |  |  | GO:0038023 | Signaling receptor activity | 0.865 |
|  |  |  | GO:0005198 | Structural molecule activity | 0.835 |
|  |  |  | GO:0030234 | Enzyme regulatory activity | 0.832 |
| 10 |  | Mcfp3 v7 | GO:0030414 | Peptidase inhibitor activity | 0.948 |
|  |  |  | GO:0004872 | Receptor activity | 0.931 |
|  |  |  | GO:0004930 | G-protein coupled receptor activity | 0.915 |
|  |  |  | GO:0004857 | Enzyme inhibitor activity | 0.878 |
|  |  |  | GO:0003735 | Structural constituent of ribosome | 0.856 |
|  |  |  | GO:0005198 | Structural molecule activity | 0.800 |
| 11 |  | Mcfp3 v8 | GO:0030414 | Peptidase inhibitor activity | 0.981 |
|  |  |  | GO:0004857 | Enzyme inhibitor activity | 0.970 |
|  |  |  | GO:0004930 | G-protein coupled receptor activity | 0.902 |
|  |  |  | GO:0004872 | Receptor activity | 0.829 |
|  |  |  | GO:0003676 | Nucleic acid binding | 0.825 |
| 12 |  | Mcfp3 v9 | GO:0004872 | Receptor activity | 0.938 |
|  |  |  | GO:0004930 | G-protein coupled receptor activity | 0.933 |
|  |  |  | GO:0030414 | Peptidase inhibitor activity | 0.931 |
|  |  |  | GO:0003735 | Structural constituent of ribosome | 0.878 |
|  |  |  | GO:0004857 | Enzyme inhibitor activity | 0.812 |
|  |  |  | GO:0003676 | Nucleic acid binding | 0.806 |
| 13 |  | Mcfp3 v10 | GO:0004872 | Receptor activity | 0.942 |
|  |  |  | GO:0030414 | Peptidase inhibitor activity | 0.936 |
|  |  |  | GO:0004930 | G-protein coupled receptor activity | 0.935 |
|  |  |  | GO:0003735 | Structural constituent of ribosome | 0.880 |
|  |  |  | GO:0004857 | Enzyme inhibitor activity | 0.824 |
|  |  |  | GO:0003676 | Nucleic acid binding | 0.807 |
| 14 |  | Mcfp3 v11 | GO:0004872 | Receptor activity | 0.937 |
|  |  |  | GO:0030414 | Peptidase inhibitor activity | 0.933 |
|  |  |  | GO:0004930 | G-protein coupled receptor activity | 0.932 |
|  |  |  | GO:0003735 | Structural constituent of ribosome | 0.874 |
|  |  |  | GO:0004857 | Enzyme inhibitor activity | 0.813 |
|  |  |  | GO:0003676 | Nucleic acid binding | 0.805 |
|  |  |  | GO:0005198 | Structural molecule activity | 0.801 |
| 15 |  | Mcfp4 v1 | GO:0001883 | Purine nucleoside binding | 0.764 |
| 16 |  | Mcfp4 v2 | GO:0003824 | Catalytic cavity | 0.938 |
| 17 |  | Mcfp5 | GO:0003824 | Catalytic cavity | 0.799 |
| 18 |  | Mcfp6 v1 | GO:0005125 | Cytokine activity |  |
|  |  |  | GO:0004175 | Endopeptidase activity | 0.874 |
| 19 |  | Mcfp6 v2 | GO:0005125 | Cytokine activity | 0.906 |
|  |  |  | GO:0004175 | Endopeptidase activity | 0.903 |
|  |  |  | GO:0008233 | Peptidase activity | 0.829 |
|  |  |  | GO:0008270 | Zinc ion binding | 0.821 |
| 20 |  | Mcfp6 v3 | GO:0005125 | Cytokine activity | 0.886 |
|  |  |  | GO:0004175 | Endopeptidase activity | 0.885 |
|  |  |  | GO:0003824 | Catalytic cavity | 0.825 |
| 21 |  | Mcfp7 v1 | GO:0003676 | Nucleic acid binding | 0.709 |
| 22 |  | Mcfp7 v2 | GO:0004930 | G-protein coupled receptor activity | 0.940 |
| 23 |  | Mcfp8 | GO:0004857 | Enzyme inhibitor activity | 0.977 |
|  |  |  | GO:0003676 | Nucleic acid binding | 0.883 |
| 24 |  | Mcfp9 v1 | GO:0048037 | Cofactor binding | 0.943 |
| 25 |  | Mcfp9 v2 | GO:0048037 | Cofactor binding | 0.910 |
| 26 |  | Mcfp10 | GO:0003824 | Catalytic cavity | 0.732 |
| 27 |  | Mcfp11 | GO:0003824 | Catalytic cavity | 0.882 |
|  |  |  | GO:0008270 | Zinc ion binding | 0.820 |
| 28 |  | Mcfp12 | GO:0016757 | Transferase activity, transferring glycosyl groups | 0.933 |
|  |  |  | GO:0016758 | Transferase activity, transferring hexosyl groups | 0.932 |
|  |  |  | GO:0016740 | Transferase activity | 0.805 |
| 29 |  | Mcfp13 | GO:0003824 | Catalytic cavity | 0.932 |
|  |  |  | GO:0016758 | Transferase activity, transferring hexosyl groups | 0.874 |
|  |  |  | GO:0005125 | Cytokine activity | 0.847 |
|  |  |  | GO:0005126 | Cytokine receptor binding | 0.828 |
|  |  |  | GO:0016757 | Transferase activity, transferring glycosyl groups | 0.824 |
| 30 |  | Mcfp14 | GO:0005125 | Cytokine activity | 0.976 |
|  |  |  | GO:0008083 | Growth factor activity | 0.928 |
|  |  |  | GO:0004930 | G-protein coupled receptor activity | 0.903 |
| 31 |  | Mcfp15 | GO:0008083 | Growth factor activity | 0.889 |
|  |  |  | GO:0003824 | Catalytic cavity | 0.863 |
|  |  |  | GO:0008270 | Zinc ion binding | 0.828 |
| 32 |  | Mcfp16 | GO:0008083 | Growth factor activity | 0.964 |
|  |  |  | GO:0008270 | Zinc ion binding | 0.962 |
|  |  |  | GO:0005125 | Cytokine activity | 0.932 |
|  |  |  | GO:0005539 | Glycosaminoglycan binding | 0.864 |
| 33 |  | Mcfp17 | GO:0003676 | Nucleic acid binding | 0.863 |
|  |  |  | GO:0008083 | Growth factor activity | 0.835 |
|  |  |  | GO:0004866 | Endopeptidase inhibitor activity | 0.817 |
|  |  |  | GO:0005125 | Cytokine activity | 0.814 |
|  |  |  | GO:0005539 | Glycosaminoglycan binding | 0.802 |
| 34 |  | Mcfp18 | GO:0008270 | Zinc ion binding | 0.999 |
|  |  |  | GO:0003824 | Catalytic cavity | 0.959 |
|  |  |  | GO:0030414 | Peptidase inhibitor activity | 0.951 |
|  |  |  | GO:0005126 | Cytokine receptor binding | 0.949 |
|  |  |  | GO:0005125 | Cytokine activity | 0.890 |
|  |  |  | GO:0004857 | Enzyme inhibitor activity | 0.857 |
|  |  |  | GO:0001664 | G-protein coupled receptor binding | 0.842 |
|  |  |  | GO:0016757 | Transferase activity, transferring glycosyl groups | 0.841 |
|  |  |  | GO:0008083 | Growth factor activity | 0.834 |
|  |  |  | GO:0030234 | Enzyme regulatory activity | 0.819 |
| 35 | *Atrina pectinata* | Apfp1 | GO:0008083 | Growth factor activity | 0.938 |
|  |  |  | GO:0005125 | Cytokine activity | 0.875 |
| 36 | *Dreissena polymorpha* | Dpfp1 | GO:0008083 | Growth factor activity | 0.980 |
|  |  |  | GO:0004930 | G-protein coupled receptor activity | 0.941 |
| 37 | *Mytilus edulis* | Mefp1 | GO:0003676 | Nucleic acid binding | 0.841 |
|  |  |  | GO:0008083 | Growth factor activity | 0.824 |
| 38 |  | Mefp2 | GO:0004872 | Receptor activity | 0.991 |
|  |  |  | GO:0004888 | Transmembrane signaling receptor activity | 0.983 |
|  |  |  | GO:0004930 | G-protein coupled receptor activity | 0.969 |
|  |  |  | GO:0038023 | Signaling receptor activity | 0.942 |
|  |  |  | GO:0005539 | Glycosaminoglycan binding | 0.894 |
|  |  |  | GO:0004175 | Endopeptidase activity | 0.891 |
|  |  |  | GO:0004871 | Signal transducer activity | 0.864 |
|  |  |  | GO:0008083 | Growth factor activity | 0.857 |
|  |  |  | GO:0044822 | Poly(A) RNA binding | 0.843 |
|  |  |  | GO:0008270 | Zinc ion binding | 0.842 |
| 39 | *Mytilus galloprovincialis* | Mgfp1 | GO:0004930 | G-protein coupled receptor activity | 0.912 |
|  |  |  | GO:0003676 | Nucleic acid binding | 0.904 |
|  |  |  | GO:0044822 | Poly(A) RNA binding | 0.842 |
|  |  |  | GO:0008083 | Growth factor activity | 0.803 |
| 40 |  | Mgfp3 v1 | GO:0001664 | G-protein coupled receptor binding | 0.976 |
|  |  |  | GO:0005125 | Cytokine activity | 0.973 |
|  |  |  | GO:0030414 | Peptidase inhibitor activity | 0.961 |
|  |  |  | GO:0008083 | Growth factor activity | 0.949 |
|  |  |  | GO:0004857 | Enzyme inhibitor activity | 0.940 |
|  |  |  | GO:0005126 | Cytokine receptor binding | 0.937 |
|  |  |  | GO:0004930 | G-protein coupled receptor activity | 0.873 |
|  |  |  | GO:0030324 | Enzyme regulatory activity | 0.836 |
| 41 |  | Mgfp3 v2 | GO:0030414 | Peptidase inhibitor activity | 0.990 |
|  |  |  | GO:0004857 | Enzyme inhibitor activity | 0.974 |
|  |  |  | GO:0005126 | Cytokine receptor binding | 0.965 |
|  |  |  | GO:0001664 | G-protein coupled receptor binding | 0.961 |
|  |  |  | GO:0005125 | Cytokine activity | 0.959 |
|  |  |  | GO:0008083 | Growth factor activity | 0.945 |
|  |  |  | GO:0004872 | Receptor activity | 0.912 |
|  |  |  | GO:0004930 | G-protein coupled receptor activity | 0.910 |
|  |  |  | GO:0030234 | Enzyme regulatory activity | 0.834 |
|  |  |  | GO:0004888 | Transmembrane signaling receptor activity | 0.801 |
| 42 | *Mytilus unguiculatus* | Mufp2 | GO:0004872 | Receptor activity | 0.993 |
|  |  |  | GO:0005539 | Glycosaminoglycan binding | 0.964 |
|  |  |  | GO:0004888 | Transmembrane signaling receptor activity | 0.963 |
|  |  |  | GO:0004930 | G-protein coupled receptor activity | 0.951 |
|  |  |  | GO:0004175 | Endopeptidase activity | 0.931 |
|  |  |  | GO:0008083 | Growth factor activity | 0.930 |
|  |  |  | GO:0004871 | Signal transducer activity | 0.904 |
|  |  |  | GO:0038023 | Signaling receptor activity | 0.901 |
|  |  |  | GO:0004252 | Serine-type endopeptidase activity | 0.897 |
|  |  |  | GO:0008270 | Zinc ion binding | 0.889 |
|  |  |  | GO:0005125 | Cytokine activity | 0.874 |
|  |  |  | GO:0017171 | Serine hydrolase activity | 0.844 |
| 43 |  | Mufp3 | GO:0030414 | Peptidase inhibitor activity | 0.985 |
|  |  |  | GO:0004857 | Enzyme inhibitor activity | 0.963 |
|  |  |  | GO:0004930 | G-protein coupled receptor activity | 0.929 |
|  |  |  | GO:0004872 | Receptor activity | 0.909 |
|  |  |  | GO:0005126 | Cytokine receptor binding | 0.834 |
|  |  |  | GO:0005125 | Cytokine activity | 0.833 |
|  |  |  | GO:0003676 | Nucleic acid binding | 0.830 |
|  |  |  | GO:0008083 | Growth factor activity | 0.811 |
|  |  |  | GO:0005198 | Structural molecule activity | 0.803 |
| 44 |  | Mufp3 v1 | GO:0030414 | Peptidase inhibitor activity | 0.988 |
|  |  |  | GO:0004857 | Enzyme inhibitor activity | 0.962 |
|  |  |  | GO:0004930 | G-protein coupled receptor activity | 0.926 |
|  |  |  | GO:0004872 | Receptor activity | 0.924 |
|  |  |  | GO:0003676 | Nucleic acid binding | 0.830 |
|  |  |  | GO:0005198 | Structural molecule activity | 0.826 |
|  |  |  | GO:0005126 | Cytokine receptor binding | 0.816 |
|  |  |  | GO:0008083 | Growth factor activity | 0.803 |
| 45 |  | Mufp3 v2 | GO:0030414 | Peptidase inhibitor activity | 0.989 |
|  |  |  | GO:0004857 | Enzyme inhibitor activity | 0.965 |
|  |  |  | GO:0004872 | Receptor activity | 0.924 |
|  |  |  | GO:0004930 | G-protein coupled receptor activity | 0.920 |
|  |  |  | GO:0003676 | Nucleic acid binding | 0.833 |
|  |  |  | GO:0005198 | Structural molecule activity | 0.832 |
|  |  |  | GO:0005126 | Cytokine receptor binding | 0.803 |
|  |  |  | GO:0008083 | Growth factor activity | 0.801 |
| 46 |  | Mufp3 v3 | GO:0004930 | G-protein coupled receptor activity | 0.933 |
|  |  |  | GO:0004872 | Receptor activity | 0.926 |
|  |  |  | GO:0030414 | Peptidase inhibitor activity | 0.917 |
|  |  |  | GO:0001664 | G-protein coupled receptor binding | 0.911 |
|  |  |  | GO:0005125 | Cytokine activity | 0.882 |
|  |  |  | GO:0008083 | Growth factor activity | 0.882 |
|  |  |  | GO:0005126 | Cytokine receptor binding | 0.879 |
|  |  |  | GO:0038023 | Signaling receptor activity | 0.874 |
|  |  |  | GO:0004888 | Transmembrane signaling receptor activity | 0.873 |
|  |  |  | GO:0005539 | Glycosaminoglycan binding | 0.831 |
|  |  |  | GO:0003735 | Structural constituent of ribosome | 0.816 |
|  |  |  | GO:0008236 | Serine type peptidase activity | 0.805 |
| 47 |  | Mufp3 v4 | GO:0004872 | Receptor activity | 0.971 |
|  |  |  | GO:0004930 | G-protein coupled receptor activity | 0.961 |
|  |  |  | GO:0030414 | Peptidase inhibitor activity | 0.958 |
|  |  |  | GO:0004857 | Enzyme inhibitor activity | 0.863 |
|  |  |  | GO:0005198 | Structural molecule activity | 0.833 |
|  |  |  | GO:0004871 | Signal transducer activity | 0.828 |
|  |  |  | GO:0003676 | Nucleic acid binding | 0.823 |
|  |  |  | GO:0000166 | Nucleotide binding | 0.805 |
| 48 |  | Mufp3 v5 | GO:0030414 | Peptidase inhibitor activity | 0.989 |
|  |  |  | GO:0004857 | Enzyme inhibitor activity | 0.964 |
|  |  |  | GO:0001664 | G-protein coupled receptor binding | 0.959 |
|  |  |  | GO:0005125 | Cytokine activity | 0.958 |
|  |  |  | GO:0005126 | Cytokine receptor binding | 0.957 |
|  |  |  | GO:0008083 | Growth factor activity | 0.941 |
|  |  |  | GO:0004930 | G-protein coupled receptor activity | 0.926 |
|  |  |  | GO:0004872 | Receptor activity | 0.925 |
|  |  |  | GO:0038023 | Signaling receptor activity | 0.894 |
|  |  |  | GO:0004888 | Transmembrane signaling receptor activity | 0.845 |
|  |  |  | GO:0030324 | Enzyme regulatory activity | 0.830 |
|  |  |  | GO:0005198 | Structural molecule activity | 0.815 |
| 49 |  | Mufp3 v6 | GO:0030414 | Peptidase inhibitor activity | 0.988 |
|  |  |  | GO:0005125 | Cytokine activity | 0.968 |
|  |  |  | GO:0001664 | G-protein coupled receptor binding | 0.964 |
|  |  |  | GO:0004857 | Enzyme inhibitor activity | 0.962 |
|  |  |  | GO:0008083 | Growth factor activity | 0.944 |
|  |  |  | GO:0004930 | G-protein coupled receptor activity | 0.921 |
|  |  |  | GO:0004872 | Receptor activity | 0.914 |
|  |  |  | GO:0038023 | Signaling receptor activity | 0.892 |
|  |  |  | GO:0030324 | Enzyme regulatory activity | 0.842 |
|  |  |  | GO:0004888 | Transmembrane signaling receptor activity | 0.820 |
| 50 |  | Mufp3 v7 | GO:0004872 | Receptor activity | 0.959 |
|  |  |  | GO:0030414 | Peptidase inhibitor activity | 0.951 |
|  |  |  | GO:0004930 | G-protein coupled receptor activity | 0.933 |
|  |  |  | GO:0004857 | Enzyme inhibitor activity | 0.855 |
|  |  |  | GO:0000166 | Nucleotide binding | 0.806 |
|  |  |  | GO:0003676 | Nucleic acid binding | 0.803 |
| 51 |  | Mufp3 v8 | GO:0030414 | Peptidase inhibitor activity | 0.986 |
|  |  |  | GO:0004857 | Enzyme inhibitor activity | 0.955 |
|  |  |  | GO:0004872 | Receptor activity | 0.933 |
|  |  |  | GO:0004930 | G-protein coupled receptor activity | 0.922 |
|  |  |  | GO:0005125 | Cytokine activity | 0.844 |
|  |  |  | GO:0005126 | Cytokine receptor binding | 0.841 |
|  |  |  | GO:0003676 | Nucleic acid binding | 0.822 |
|  |  |  | GO:0008083 | Growth factor activity | 0.821 |
|  |  |  | GO:0004871 | Signal transducer activity | 0.803 |
| 52 |  | Mufp3 v9 | GO:0004872 | Receptor activity | 0.955 |
|  |  |  | GO:0030414 | Peptidase inhibitor activity | 0.944 |
|  |  |  | GO:0004930 | G-protein coupled receptor activity | 0.928 |
|  |  |  | GO:0004857 | Enzyme inhibitor activity | 0.838 |
|  |  |  | GO:0000166 | Nucleotide binding | 0.831 |
| 53 |  | Mufp3 v10 | GO:0030414 | Peptidase inhibitor activity | 0.988 |
|  |  |  | GO:0004857 | Enzyme inhibitor activity | 0.966 |
|  |  |  | GO:0005125 | Cytokine activity | 0.960 |
|  |  |  | GO:0001664 | G-protein coupled receptor binding | 0.958 |
|  |  |  | GO:0005126 | Cytokine receptor binding | 0.953 |
|  |  |  | GO:0008083 | Growth factor activity | 0.943 |
|  |  |  | GO:0004930 | G-protein coupled receptor activity | 0.934 |
|  |  |  | GO:0004872 | Receptor activity | 0.922 |
|  |  |  | GO:0038023 | Signaling receptor activity | 0.893 |
|  |  |  | GO:0004888 | Transmembrane signaling receptor activity | 0.852 |
|  |  |  | GO:0005198 | Structural molecule activity | 0.821 |
|  |  |  | GO:0030324 | Enzyme regulatory activity | 0.821 |
|  |  |  | GO:0008236 | Serine-type peptidase activity | 0.816 |
| 54 |  | Mufp3 v11 | GO:0004872 | Receptor activity | 0.961 |
|  |  |  | GO:0030414 | Peptidase inhibitor activity | 0.949 |
|  |  |  | GO:0001664 | G-protein coupled receptor binding | 0.947 |
|  |  |  | GO:0004930 | G-protein coupled receptor activity | 0.912 |
|  |  |  | GO:0038023 | Signaling receptor activity | 0.907 |
|  |  |  | GO:0005126 | Cytokine receptor binding | 0.897 |
|  |  |  | GO:0008083 | Growth factor activity | 0.895 |
|  |  |  | GO:0004857 | Enzyme inhibitor activity | 0.887 |
|  |  |  | GO:0005125 | Cytokine activity | 0.857 |
|  |  |  | GO:0004888 | Transmembrane signaling receptor activity | 0.857 |
|  |  |  | GO:0005198 | Structural molecule activity | 0.833 |
|  |  |  | GO:0004871 | Signal transducer activity | 0.805 |
| 55 |  | Mufp3 v12 | GO:0004872 | Receptor activity | 0.958 |
|  |  |  | GO:0030414 | Peptidase inhibitor activity | 0.952 |
|  |  |  | GO:0004930 | G-protein coupled receptor activity | 0.933 |
|  |  |  | GO:0004857 | Enzyme inhibitor activity | 0.855 |
|  |  |  | GO:0000166 | Nucleotide binding | 0.807 |
|  |  |  | GO:0003676 | Nucleic acid binding | 0.803 |
| 56 |  | Mufp3 v13 | GO:0030414 | Peptidase inhibitor activity | 0.984 |
|  |  |  | GO:0004857 | Enzyme inhibitor activity | 0.965 |
|  |  |  | GO:0001664 | G-protein coupled receptor binding | 0.964 |
|  |  |  | GO:0004872 | Receptor activity | 0.955 |
|  |  |  | GO:0005126 | Cytokine receptor binding | 0.943 |
|  |  |  | GO:0005125 | Cytokine activity | 0.938 |
|  |  |  | GO:0008083 | Growth factor activity | 0.938 |
|  |  |  | GO:0004930 | G-protein coupled receptor activity | 0.913 |
|  |  |  | GO:0038023 | Signaling receptor activity | 0.903 |
|  |  |  | GO:0005198 | Structural molecule activity | 0.863 |
|  |  |  | GO:0004888 | Transmembrane signaling receptor activity | 0.827 |
|  |  |  | GO:0030324 | Enzyme regulatory activity | 0.818 |
| 57 |  | Mufp3 v14 | GO:0004872 | Receptor activity | 0.955 |
|  |  |  | GO:0004930 | G-protein coupled receptor activity | 0.952 |
|  |  |  | GO:0030414 | Peptidase inhibitor activity | 0.945 |
|  |  |  | GO:0001664 | G-protein coupled receptor binding | 0.934 |
|  |  |  | GO:0008083 | Growth factor activity | 0.904 |
|  |  |  | GO:0008236 | Serine-type peptidase activity | 0.898 |
|  |  |  | GO:0004888 | Transmembrane signaling receptor activity | 0.891 |
|  |  |  | GO:0038023 | Signaling receptor activity | 0.890 |
|  |  |  | GO:0005125 | Cytokine activity | 0.887 |
|  |  |  | GO:0005126 | Cytokine receptor binding | 0.887 |
|  |  |  | GO:0004857 | Enzyme inhibitor activity | 0.850 |
|  |  |  | GO:0005198 | Structural molecule activity | 0.822 |
|  |  |  | GO:0005539 | Glycosaminoglycan binding | 0.800 |
| 58 |  | Mufp6 | GO:0005125 | Cytokine activity | 0.941 |
|  |  |  | GO:0004175 | Endopeptidase activity | 0.856 |
|  |  |  | GO:0008270 | Zinc ion binding | 0.846 |
|  |  |  | GO:0005126 | Cytokine receptor binding | 0.834 |
| 59 |  | Mufp6 v1 | GO:0005125 | Cytokine activity | 0.958 |
|  |  |  | GO:0005126 | Cytokine receptor binding | 0.903 |
|  |  |  | GO:0008083 | Growth factor activity | 0.864 |
| 60 |  | Mufp6 v2 | GO:0005125 | Cytokine activity | 0.949 |
|  |  |  | GO:0005126 | Cytokine receptor binding | 0.858 |
|  |  |  | GO:0008083 | Growth factor activity | 0.831 |
| 61 |  | Mufp6 v3 | GO:0005125 | Cytokine activity | 0.945 |
|  |  |  | GO:0005126 | Cytokine receptor binding | 0.861 |
|  |  |  | GO:0008083 | Growth factor activity | 0.816 |
|  |  |  | GO:0008270 | Zinc ion binding | 0.815 |
| 62 |  | Mufp6 v4 | GO:0005125 | Cytokine activity | 0.953 |
|  |  |  | GO:0005126 | Cytokine receptor binding | 0.899 |
|  |  |  | GO:0008083 | Growth factor activity | 0.857 |
| 63 |  | Mufp6 v5 | GO:0005125 | Cytokine activity | 0.961 |
|  |  |  | GO:0005126 | Cytokine receptor binding | 0.899 |
|  |  |  | GO:0008083 | Growth factor activity | 0.831 |
| 64 |  | Mufp6 v6 | GO:0005125 | Cytokine activity | 0.940 |
|  |  |  | GO:0005126 | Cytokine receptor binding | 0.876 |
|  |  |  | GO:0008083 | Growth factor activity | 0.821 |
| 65 |  | Mufp6 v7 | GO:0005125 | Cytokine activity | 0.968 |
|  |  |  | GO:0005126 | Cytokine receptor binding | 0.925 |
|  |  |  | GO:0008083 | Growth factor activity | 0.899 |
|  |  |  | GO:0001664 | G-protein coupled receptor binding | 0.877 |
| 66 |  | Mufp6 v8 | GO:0005125 | Cytokine activity | 0.964 |
|  |  |  | GO:0005126 | Cytokine receptor binding | 0.909 |
|  |  |  | GO:0008083 | Growth factor activity | 0.900 |
|  |  |  | GO:0038023 | Signaling receptor activity | 0.884 |
|  |  |  | GO:0001664 | G-protein coupled receptor binding | 0.819 |
| 67 |  | Mufp6 v9 | GO:0003824 | Catalytic activity | 0.989 |
|  |  |  | GO:0005198 | Structural molecule activity | 0.985 |
|  |  |  | GO:0008270 | Zinc ion binding | 0.961 |
|  |  |  | GO:0003676 | Nucleic acid binding | 0.938 |
|  |  |  | GO:0016491 | Oxidoreductase activity | 0.932 |
|  |  |  | GO:0015267 | Channel activity | 0.918 |
|  |  |  | GO:0003779 | Actin binding | 0.916 |
|  |  |  | GO:0044822 | Poly(A) RNA binding | 0.869 |
|  |  |  | GO:0038023 | Signaling receptor activity | 0.868 |
|  |  |  | GO:0016740 | Transferase activity | 0.863 |
|  |  |  | GO:0046914 | Transition metal ion binding | 0.857 |
|  |  |  | GO:0004871 | Signal transducer activity | 0.851 |
|  |  |  | GO:0022891 | Substrate-specific transmembrane transported activity | 0.820 |
|  |  |  | GO:0004175 | Endopeptidase activity | 0.804 |
| 68 | *Mizuhopecten yessoensis* | Myfp1 v1 | GO:0044822 | Poly(A) RNA binding | 0.980 |
|  |  |  | GO:0003676 | Nucleic acid binding | 0.960 |
|  |  |  | GO:0003677 | DNA binding | 0.922 |
|  |  |  | GO:0008092 | Cytoskeleton protein binding | 0.900 |
| 69 |  | Myfp1 v2 | GO:0003676 | Nucleic acid binding | 0.987 |
|  |  |  | GO:0003677 | DNA binding | 0.960 |
|  |  |  | GO:0008092 | Cytoskeleton protein binding | 0.880 |
|  |  |  | GO:0001071 | Nucleic acid binding transcriptome factor activity | 0.845 |
|  |  |  | GO:0003700 | Sequence-specific DNA binding transcription factor activity | 0.833 |
| 70 | *Perna canaliculus* | Pcfp1 v1 | GO:0003676 | Nucleic acid binding | 0.987 |
|  |  |  | GO:0003677 | DNA binding | 0.960 |
|  |  |  | GO:0008092 | Cytoskeleton protein binding | 0.880 |
|  |  |  | GO:0001071 | Nucleic acid binding transcriptome factor activity | 0.845 |
|  |  |  | GO:0003700 | Sequence-specific DNA binding transcription factor activity | 0.833 |
| 71 |  | Pcfp1 v2 | GO:0044822 | Poly(A) RNA binding | 0.979 |
| 72 |  | Pcfp1 v3 | GO:0044822 | Poly(A) RNA binding | 0.958 |
|  |  |  | GO:0004888 | Transmembrane signaling receptor activity | 0.924 |
|  |  |  | GO:0038023 | Signaling receptor activity | 0.899 |
|  |  |  | GO:0004871 | Signal transducer activity | 0.849 |
| 73 |  | Pcfp1 v4 | GO:0044822 | Poly(A) RNA binding | 0.947 |
|  |  |  | GO:0038023 | Signaling receptor activity | 0.904 |
|  |  |  | GO:0004871 | Signal transducer activity | 0.855 |
|  |  |  | GO:0004888 | Transmembrane signaling receptor activity | 0.835 |
| 74 | *Perna viridis* | Pvfp1 v1 | GO:0001664 | G-protein coupled receptor binding | 0.843 |
| 75 |  | Pvfp1 v2 | GO:0005539 | Glycosaminoglycan binding | 0.846 |
| 76 |  | Pvfp3 | GO:0008270 | Zinc ion binding | 0.980 |
|  |  |  | GO:0038023 | Signaling receptor activity | 0.943 |
|  |  |  | GO:0030414 | Peptidase inhibitory activity | 0.939 |
|  |  |  | GO:0004857 | Enzyme inhibitor activity | 0.937 |
|  |  |  | GO:0003824 | Catalytic activity | 0.906 |
|  |  |  | GO:0000166 | Nucleotide binding | 0.897 |
|  |  |  | GO:0005126 | Cytokine receptor binding | 0.843 |
|  |  |  | GO:0005125 | Cytokine activity | 0.832 |
|  |  |  | GO:0004871 | Signal transducer activity | 0.818 |
|  |  |  | GO:0004930 | G-protein coupled receptor activity | 0.817 |
|  |  |  | GO:0005198 | Structural molecule activity | 0.807 |
| 77 |  | Pvfp5 | GO:0004888 | Transmembrane signaling receptor activity | 0.986 |
|  |  |  | GO:0038023 | Signaling receptor activity | 0.979 |
|  |  |  | GO:0004872 | Receptor activity | 0.975 |
|  |  |  | GO:0004871 | Signal transducer activity | 0.944 |
|  |  |  | GO:0004930 | G-protein coupled receptor activity | 0.921 |
|  |  |  | GO:0005125 | Cytokine activity | 0.914 |
|  |  |  | GO:0008083 | Growth factor activity | 0.910 |
|  |  |  | GO:0008270 | Zinc ion binding | 0.881 |
|  |  |  | GO:0004175 | Endopeptidase activity | 0.849 |
|  |  |  | GO:0005539 | Glycosaminoglycan binding | 0.837 |
|  |  |  | GO:0005126 | Cytokine receptor binding | 0.818 |
|  |  |  | GO:0005216 | Ion channel activity | 0.802 |
| 78 |  | Pvfp6 | GO:0005125 | Cytokine activity | 0.964 |
|  |  |  | GO:0001664 | G-protein coupled receptor binding | 0.958 |
|  |  |  | GO:0005126 | Cytokine receptor binding | 0.947 |
|  |  |  | GO:0030414 | Peptidase inhibitory activity | 0.935 |
|  |  |  | GO:0008083 | Growth factor activity | 0.934 |
|  |  |  | GO:0038023 | Signaling receptor activity | 0.921 |
|  |  |  | GO:0004857 | Enzyme inhibitor activity | 0.895 |
|  |  |  | GO:0004872 | Receptor activity | 0.861 |
|  |  |  | GO:0030234 | Enzyme regulator activity | 0.811 |

**Section 4 (S4): Amino acid composition of each Mussel foot proteins**

***Atrina pectinata***

COMPOSITIONAL ANALYSIS

A : 16( 4.5%); C : 10( 2.8%); D : 18( 5.1%); E : 9( 2.6%); F : 7( 2.0%)

G : 28( 8.0%); H : 5( 1.4%); I- : 6( 1.7%); K++: 54(15.3%); L- : 16( 4.5%)

M : 7( 2.0%); N : 6( 1.7%); P++: 53(15.1%); Q- : 5( 1.4%); R- : 4( 1.1%)

S : 15( 4.3%); T--: 4( 1.1%); V+ : 35( 9.9%); W : 10( 2.8%); Y++: 44(12.5%)

***Dreissena polymorpha***

COMPOSITIONAL ANALYSIS

A--: 3( 0.7%); C : 1( 0.2%); D++: 45(10.5%); E : 11( 2.6%); F : 8( 1.9%)

G : 30( 7.0%); H- : 2( 0.5%); I- : 5( 1.2%); K+ : 44(10.2%); L--: 11( 2.6%)

M--: 1( 0.2%); N : 11( 2.6%); P++: 96(22.3%); Q : 11( 2.6%); R- : 6( 1.4%)

S : 18( 4.2%); T : 34( 7.9%); V : 24( 5.6%); W : 5( 1.2%); Y++: 64(14.9%)

***Mytilus californianus***

Mcfp1 v1

COMPOSITIONAL ANALYSIS

A- : 25( 3.4%); C : 3( 0.4%); D--: 1( 0.1%); E--: 1( 0.1%); F--: 3( 0.4%)

G--: 9( 1.2%); H- : 4( 0.5%); I : 28( 3.8%); K++:149(20.4%); L--: 11( 1.5%)

M--: 2( 0.3%); N- : 7( 1.0%); P++:173(23.6%); Q--: 4( 0.5%); R--: 7( 1.0%)

S : 81(11.1%); T+ : 81(11.1%); V--: 6( 0.8%); W : 0( 0.0%); Y++:137(18.7%)

Mcfp1 v2

COMPOSITIONAL ANALYSIS

A- : 23( 3.4%); C : 3( 0.4%); D--: 1( 0.1%); E--: 1( 0.1%); F--: 3( 0.4%)

G--: 9( 1.3%); H- : 4( 0.6%); I : 27( 4.0%); K++:135(20.1%); L--: 11( 1.6%)

M--: 2( 0.3%); N- : 7( 1.0%); P++:156(23.2%); Q--: 4( 0.6%); R- : 7( 1.0%)

S : 74(11.0%); T+ : 74(11.0%); V--: 6( 0.9%); W : 0( 0.0%); Y++:125(18.6%)

Mcfp2

COMPOSITIONAL ANALYSIS

A- : 23( 3.4%); C : 3( 0.4%); D--: 1( 0.1%); E--: 1( 0.1%); F--: 3( 0.4%)

G--: 9( 1.3%); H- : 4( 0.6%); I : 27( 4.0%); K++:135(20.1%); L--: 11( 1.6%)

M--: 2( 0.3%); N- : 7( 1.0%); P++:156(23.2%); Q--: 4( 0.6%); R- : 7( 1.0%)

S : 74(11.0%); T+ : 74(11.0%); V--: 6( 0.9%); W : 0( 0.0%); Y++:125(18.6%)

Mcfp3 v1

COMPOSITIONAL ANALYSIS

A : 3( 4.5%); C : 0( 0.0%); D : 1( 1.5%); E : 0( 0.0%); F : 1( 1.5%)

G : 12(18.2%); H : 0( 0.0%); I : 2( 3.0%); K : 8(12.1%); L : 4( 6.1%)

M : 1( 1.5%); N : 4( 6.1%); P : 3( 4.5%); Q : 1( 1.5%); R : 4( 6.1%)

S : 4( 6.1%); T : 0( 0.0%); V : 5( 7.6%); W : 3( 4.5%); Y : 10(15.2%)

Mcfp3 v2

COMPOSITIONAL ANALYSIS

A : 4( 5.9%); C : 0( 0.0%); D : 1( 1.5%); E : 0( 0.0%); F : 1( 1.5%)

G : 12(17.6%); H : 0( 0.0%); I : 1( 1.5%); K : 7(10.3%); L : 5( 7.4%)

M : 1( 1.5%); N : 5( 7.4%); P : 3( 4.4%); Q : 1( 1.5%); R : 4( 5.9%)

S : 4( 5.9%); T : 0( 0.0%); V : 5( 7.4%); W : 4( 5.9%); Y : 10(14.7%)

Mcfp3 v3

COMPOSITIONAL ANALYSIS

A : 4( 5.1%); C : 0( 0.0%); D : 2( 2.6%); E : 0( 0.0%); F : 3( 3.8%)

G : 16(20.5%); H : 1( 1.3%); I : 1( 1.3%); K : 3( 3.8%); L : 6( 7.7%)

M : 1( 1.3%); N : 10(12.8%); P : 3( 3.8%); Q : 1( 1.3%); R : 1( 1.3%)

S : 3( 3.8%); T : 1( 1.3%); V : 4( 5.1%); W : 4( 5.1%); Y : 14(17.9%)

Mcfp3 v4

COMPOSITIONAL ANALYSIS

A : 3( 3.8%); C : 0( 0.0%); D : 2( 2.6%); E : 0( 0.0%); F : 3( 3.8%)

G : 15(19.2%); H : 1( 1.3%); I : 1( 1.3%); K : 4( 5.1%); L : 5( 6.4%)

M : 1( 1.3%); N : 10(12.8%); P : 3( 3.8%); Q : 1( 1.3%); R : 1( 1.3%)

S : 4( 5.1%); T : 1( 1.3%); V : 4( 5.1%); W : 4( 5.1%); Y : 15(19.2%)

Mcfp3 v5

COMPOSITIONAL ANALYSIS

A : 3( 3.8%); C : 0( 0.0%); D : 2( 2.6%); E : 0( 0.0%); F : 3( 3.8%)

G : 15(19.2%); H : 1( 1.3%); I : 1( 1.3%); K : 4( 5.1%); L : 5( 6.4%)

M : 1( 1.3%); N : 11(14.1%); P : 3( 3.8%); Q : 1( 1.3%); R : 1( 1.3%)

S : 3( 3.8%); T : 1( 1.3%); V : 4( 5.1%); W : 4( 5.1%); Y : 15(19.2%)

Mcfp3 v6

COMPOSITIONAL ANALYSIS

A : 3( 3.8%); C : 0( 0.0%); D : 3( 3.8%); E : 0( 0.0%); F : 3( 3.8%)

G : 15(19.2%); H : 0( 0.0%); I : 1( 1.3%); K : 8(10.3%); L : 5( 6.4%)

M : 1( 1.3%); N : 7( 9.0%); P : 3( 3.8%); Q : 1( 1.3%); R : 3( 3.8%)

S : 2( 2.6%); T : 1( 1.3%); V : 4( 5.1%); W : 5( 6.4%); Y : 13(16.7%)

MCfp3 v7

COMPOSITIONAL ANALYSIS

A : 3( 4.0%); C : 0( 0.0%); D : 3( 4.0%); E : 0( 0.0%); F : 3( 4.0%)

G : 15(20.0%); H : 1( 1.3%); I : 1( 1.3%); K : 3( 4.0%); L : 5( 6.7%)

M : 1( 1.3%); N : 10(13.3%); P : 3( 4.0%); Q : 1( 1.3%); R : 1( 1.3%)

S : 2( 2.7%); T : 1( 1.3%); V : 4( 5.3%); W : 4( 5.3%); Y : 14(18.7%)

Mcfp3 v8

COMPOSITIONAL ANALYSIS

A : 3( 4.3%); C : 0( 0.0%); D : 2( 2.9%); E : 0( 0.0%); F : 2( 2.9%)

G : 13(18.8%); H : 1( 1.4%); I : 1( 1.4%); K : 3( 4.3%); L : 5( 7.2%)

M : 1( 1.4%); N : 10(14.5%); P : 3( 4.3%); Q : 1( 1.4%); R : 1( 1.4%)

S : 2( 2.9%); T : 1( 1.4%); V : 4( 5.8%); W : 4( 5.8%); Y : 12(17.4%)

Mcfp3 v9

COMPOSITIONAL ANALYSIS

A : 3( 4.3%); C : 0( 0.0%); D : 2( 2.9%); E : 0( 0.0%); F : 2( 2.9%)

G : 14(20.3%); H : 1( 1.4%); I : 1( 1.4%); K : 2( 2.9%); L : 6( 8.7%)

M : 1( 1.4%); N : 10(14.5%); P : 3( 4.3%); Q : 1( 1.4%); R : 1( 1.4%)

S : 2( 2.9%); T : 1( 1.4%); V : 4( 5.8%); W : 4( 5.8%); Y : 11(15.9%)

Mcfp3 v10

COMPOSITIONAL ANALYSIS

A : 3( 4.3%); C : 0( 0.0%); D : 2( 2.9%); E : 0( 0.0%); F : 2( 2.9%)

G : 14(20.3%); H : 1( 1.4%); I : 1( 1.4%); K : 2( 2.9%); L : 6( 8.7%)

M : 1( 1.4%); N : 9(13.0%); P : 3( 4.3%); Q : 1( 1.4%); R : 1( 1.4%)

S : 2( 2.9%); T : 2( 2.9%); V : 4( 5.8%); W : 4( 5.8%); Y : 11(15.9%)

Mcfp3 v11

COMPOSITIONAL ANALYSIS

A : 4( 5.8%); C : 0( 0.0%); D : 2( 2.9%); E : 0( 0.0%); F : 3( 4.3%)

G : 14(20.3%); H : 1( 1.4%); I : 1( 1.4%); K : 2( 2.9%); L : 5( 7.2%)

M : 1( 1.4%); N : 9(13.0%); P : 3( 4.3%); Q : 1( 1.4%); R : 1( 1.4%)

S : 2( 2.9%); T : 1( 1.4%); V : 4( 5.8%); W : 4( 5.8%); Y : 11(15.9%)

Mcfp4 v1

COMPOSITIONAL ANALYSIS

A : 36( 4.7%); C : 2( 0.3%); D : 38( 4.9%); E- : 18( 2.3%); F- : 11( 1.4%)

G : 40( 5.2%); H++:177(23.0%); I : 34( 4.4%); K : 42( 5.5%); L : 55( 7.1%)

M- : 6( 0.8%); N : 44( 5.7%); P--: 5( 0.6%); Q : 34( 4.4%); R : 54( 7.0%)

S : 34( 4.4%); T- : 23( 3.0%); V++: 99(12.9%); W : 1( 0.1%); Y : 17( 2.2%)

Mcfp4 v2

COMPOSITIONAL ANALYSIS

A : 36( 4.4%); C : 2( 0.2%); D : 39( 4.8%); E- : 18( 2.2%); F- : 11( 1.4%)

G : 42( 5.2%); H++:193(23.8%); I : 34( 4.2%); K : 42( 5.2%); L : 60( 7.4%)

M- : 6( 0.7%); N : 43( 5.3%); P--: 5( 0.6%); Q : 34( 4.2%); R : 64( 7.9%)

S : 34( 4.2%); T- : 23( 2.8%); V++:106(13.1%); W : 1( 0.1%); Y : 17( 2.1%)

Mcfp5

COMPOSITIONAL ANALYSIS

A : 4( 4.2%); C : 2( 2.1%); D : 3( 3.1%); E : 0( 0.0%); F : 1( 1.0%)

G : 17(17.7%); H : 4( 4.2%); I : 1( 1.0%); K : 16(16.7%); L : 6( 6.2%)

M : 1( 1.0%); N : 1( 1.0%); P : 2( 2.1%); Q : 0( 0.0%); R : 3( 3.1%)

S : 8( 8.3%); T : 2( 2.1%); V : 5( 5.2%); W : 0( 0.0%); Y : 20(20.8%)

Mcfp6 v1

COMPOSITIONAL ANALYSIS

A : 3( 2.5%); C : 12( 9.9%); D : 6( 5.0%); E : 1( 0.8%); F : 5( 4.1%)

G : 16(13.2%); H : 0( 0.0%); I : 5( 4.1%); K : 9( 7.4%); L : 4( 3.3%)

M : 2( 1.7%); N : 10( 8.3%); P : 3( 2.5%); Q : 1( 0.8%); R : 6( 5.0%)

S : 10( 8.3%); T : 2( 1.7%); V : 4( 3.3%); W : 0( 0.0%); Y : 22(18.2%)

Mcfp6 v2

COMPOSITIONAL ANALYSIS

A : 5( 4.1%); C : 12( 9.9%); D : 4( 3.3%); E : 1( 0.8%); F : 5( 4.1%)

G : 15(12.4%); H : 0( 0.0%); I : 5( 4.1%); K : 10( 8.3%); L : 4( 3.3%)

M : 2( 1.7%); N : 10( 8.3%); P : 3( 2.5%); Q : 1( 0.8%); R : 8( 6.6%)

S : 9( 7.4%); T : 3( 2.5%); V : 4( 3.3%); W : 0( 0.0%); Y : 20(16.5%)

Mcfp6 v3

COMPOSITIONAL ANALYSIS

A : 5( 4.1%); C : 12( 9.9%); D : 4( 3.3%); E : 1( 0.8%); F : 4( 3.3%)

G : 15(12.4%); H : 0( 0.0%); I : 5( 4.1%); K : 10( 8.3%); L : 4( 3.3%)

M : 2( 1.7%); N : 9( 7.4%); P : 3( 2.5%); Q : 1( 0.8%); R : 8( 6.6%)

S : 11( 9.1%); T : 3( 2.5%); V : 4( 3.3%); W : 0( 0.0%); Y : 20(16.5%)

Mcfp7 v1

COMPOSITIONAL ANALYSIS

A : 4( 6.8%); C : 0( 0.0%); D : 0( 0.0%); E : 0( 0.0%); F : 1( 1.7%)

G : 11(18.6%); H : 4( 6.8%); I : 3( 5.1%); K : 5( 8.5%); L : 3( 5.1%)

M : 3( 5.1%); N : 0( 0.0%); P : 1( 1.7%); Q : 0( 0.0%); R : 4( 6.8%)

S : 8(13.6%); T : 1( 1.7%); V : 5( 8.5%); W : 0( 0.0%); Y : 6(10.2%)

Mcfp7 v2

COMPOSITIONAL ANALYSIS

A : 4( 6.2%); C : 0( 0.0%); D : 0( 0.0%); E : 0( 0.0%); F : 1( 1.5%)

G : 15(23.1%); H : 4( 6.2%); I : 1( 1.5%); K : 9(13.8%); L : 4( 6.2%)

M : 3( 4.6%); N : 0( 0.0%); P : 2( 3.1%); Q : 0( 0.0%); R : 1( 1.5%)

S : 8(12.3%); T : 1( 1.5%); V : 6( 9.2%); W : 0( 0.0%); Y : 6( 9.2%)

Mcfp8

COMPOSITIONAL ANALYSIS

A : 4( 5.8%); C : 0( 0.0%); D : 0( 0.0%); E : 0( 0.0%); F : 1( 1.4%)

G : 19(27.5%); H : 1( 1.4%); I : 3( 4.3%); K : 11(15.9%); L : 4( 5.8%)

M : 3( 4.3%); N : 0( 0.0%); P : 2( 2.9%); Q : 0( 0.0%); R : 1( 1.4%)

S : 2( 2.9%); T : 1( 1.4%); V : 6( 8.7%); W : 0( 0.0%); Y : 11(15.9%)

Mcfp9 v1

COMPOSITIONAL ANALYSIS

A : 7( 5.5%); C : 0( 0.0%); D : 2( 1.6%); E : 1( 0.8%); F : 2( 1.6%)

G : 27(21.1%); H : 25(19.5%); I : 4( 3.1%); K : 6( 4.7%); L : 3( 2.3%)

M : 1( 0.8%); N : 2( 1.6%); P : 2( 1.6%); Q : 0( 0.0%); R : 3( 2.3%)

S : 13(10.2%); T : 2( 1.6%); V : 19(14.8%); W : 1( 0.8%); Y : 8( 6.2%)

Mcfp9 v2

COMPOSITIONAL ANALYSIS

A : 6( 4.8%); C : 0( 0.0%); D : 2( 1.6%); E : 1( 0.8%); F : 2( 1.6%)

G : 25(20.0%); H : 26(20.8%); I : 4( 3.2%); K : 6( 4.8%); L : 3( 2.4%)

M : 1( 0.8%); N : 3( 2.4%); P : 2( 1.6%); Q : 0( 0.0%); R : 3( 2.4%)

S : 12( 9.6%); T : 3( 2.4%); V : 17(13.6%); W : 1( 0.8%); Y : 8( 6.4%)

Mcfp10

COMPOSITIONAL ANALYSIS

A : 16( 5.0%); C : 11( 3.4%); D+ : 26( 8.2%); E--: 1( 0.3%); F : 21( 6.6%)

G : 33(10.3%); H : 7( 2.2%); I : 13( 4.1%); K : 18( 5.6%); L : 19( 6.0%)

M : 3( 0.9%); N : 23( 7.2%); P- : 4( 1.3%); Q : 7( 2.2%); R : 15( 4.7%)

S : 30( 9.4%); T--: 6( 1.9%); V : 27( 8.5%); W : 6( 1.9%); Y++: 33(10.3%)

Mcfp11

COMPOSITIONAL ANALYSIS

A : 26( 5.2%); C : 10( 2.0%); D- : 9( 1.8%); E- : 11( 2.2%); F : 20( 4.0%)

G : 55(11.1%); H++: 86(17.3%); I : 20( 4.0%); K : 37( 7.5%); L : 29( 5.8%)

M- : 3( 0.6%); N : 18( 3.6%); P--: 3( 0.6%); Q : 9( 1.8%); R : 33( 6.7%)

S : 36( 7.3%); T- : 12( 2.4%); V : 20( 4.0%); W : 5( 1.0%); Y++: 54(10.9%)

Mcfp12

COMPOSITIONAL ANALYSIS

A : 30( 4.3%); C : 19( 2.7%); D : 24( 3.5%); E- : 12( 1.7%); F : 35( 5.0%)

G : 74(10.7%); H++: 74(10.7%); I : 27( 3.9%); K : 47( 6.8%); L : 42( 6.1%)

M- : 5( 0.7%); N : 28( 4.0%); P- : 9( 1.3%); Q : 15( 2.2%); R+ : 64( 9.2%)

S : 60( 8.6%); T- : 16( 2.3%); V : 30( 4.3%); W : 5( 0.7%); Y++: 78(11.2%)

Mcfp13

OMPOSITIONAL ANALYSIS

A : 8( 6.3%); C : 3( 2.4%); D : 0( 0.0%); E : 0( 0.0%); F : 6( 4.7%)

G : 10( 7.9%); H : 1( 0.8%); I : 8( 6.3%); K : 13(10.2%); L : 9( 7.1%)

M : 1( 0.8%); N : 7( 5.5%); P : 5( 3.9%); Q : 2( 1.6%); R : 14(11.0%)

S : 8( 6.3%); T : 5( 3.9%); V : 6( 4.7%); W : 1( 0.8%); Y : 20(15.7%)

Mcfp14

COMPOSITIONAL ANALYSIS

A : 5( 4.2%); C : 12(10.0%); D : 4( 3.3%); E : 4( 3.3%); F : 4( 3.3%)

G : 12(10.0%); H : 2( 1.7%); I : 10( 8.3%); K : 5( 4.2%); L : 6( 5.0%)

M : 1( 0.8%); N : 8( 6.7%); P : 7( 5.8%); Q : 3( 2.5%); R : 8( 6.7%)

S : 9( 7.5%); T : 8( 6.7%); V : 8( 6.7%); W : 1( 0.8%); Y : 3( 2.5%)

Mcfp15

COMPOSITIONAL ANALYSIS

A : 12( 4.8%); C+ : 14( 5.6%); D : 8( 3.2%); E- : 5( 2.0%); F : 13( 5.2%)

G : 22( 8.8%); H+ : 14( 5.6%); I : 8( 3.2%); K : 21( 8.4%); L- : 12( 4.8%)

M- : 1( 0.4%); N+ : 21( 8.4%); P : 9( 3.6%); Q : 10( 4.0%); R : 15( 6.0%)

S : 20( 8.0%); T- : 6( 2.4%); V : 12( 4.8%); W : 2( 0.8%); Y++: 24( 9.6%)

MCfp16

COMPOSITIONAL ANALYSIS

A : 2( 2.1%); C : 12(12.8%); D : 6( 6.4%); E : 4( 4.3%); F : 5( 5.3%)

G : 11(11.7%); H : 2( 2.1%); I : 6( 6.4%); K : 14(14.9%); L : 2( 2.1%)

M : 1( 1.1%); N : 3( 3.2%); P : 2( 2.1%); Q : 3( 3.2%); R : 8( 8.5%)

S : 3( 3.2%); T : 3( 3.2%); V : 5( 5.3%); W : 0( 0.0%); Y : 2( 2.1%)

Mcfp17

COMPOSITIONAL ANALYSIS

A- : 5( 2.5%); C++: 15( 7.4%); D : 10( 4.9%); E- : 3( 1.5%); F : 5( 2.5%)

G : 13( 6.4%); H- : 1( 0.5%); I : 5( 2.5%); K : 17( 8.4%); L : 18( 8.9%)

M : 3( 1.5%); N : 8( 3.9%); P : 11( 5.4%); Q : 10( 4.9%); R : 11( 5.4%)

S : 17( 8.4%); T : 13( 6.4%); V : 13( 6.4%); W+ : 8( 3.9%); Y++: 17( 8.4%)

Mcfp18

COMPOSITIONAL ANALYSIS

A : 8(11.1%); C : 11(15.3%); D : 1( 1.4%); E : 1( 1.4%); F : 1( 1.4%)

G : 5( 6.9%); H : 5( 6.9%); I : 1( 1.4%); K : 6( 8.3%); L : 5( 6.9%)

M : 1( 1.4%); N : 4( 5.6%); P : 1( 1.4%); Q : 2( 2.8%); R : 2( 2.8%)

S : 3( 4.2%); T : 4( 5.6%); V : 5( 6.9%); W : 0( 0.0%); Y : 6( 8.3%)

***Mytilus edulis***

Mefp1

COMPOSITIONAL ANALYSIS

A : 37( 6.5%); C : 3( 0.5%); D--: 2( 0.4%); E--: 2( 0.4%); F--: 3( 0.5%)

G--: 7( 1.2%); H--: 1( 0.2%); I- : 11( 1.9%); K++:112(19.8%); L--: 12( 2.1%)

M- : 2( 0.4%); N- : 9( 1.6%); P++:130(23.0%); Q--: 3( 0.5%); R--: 1( 0.2%)

S : 52( 9.2%); T++: 69(12.2%); V--: 7( 1.2%); W : 0( 0.0%); Y++:102(18.1%)

Mefp2

COMPOSITIONAL ANALYSIS

A- : 13( 2.6%); C++: 73(14.5%); D : 19( 3.8%); E- : 11( 2.2%); F- : 8( 1.6%)

G+ : 67(13.3%); H- : 3( 0.6%); I--: 4( 0.8%); K+ : 59(11.7%); L--: 12( 2.4%)

M--: 1( 0.2%); N+ : 44( 8.7%); P+ : 49( 9.7%); Q- : 6( 1.2%); R : 26( 5.2%)

S : 30( 6.0%); T : 22( 4.4%); V : 20( 4.0%); W : 0( 0.0%); Y++: 37( 7.3%)

***Mytilus galloprovincialis***

Mgfp1

COMPOSITIONAL ANALYSIS

A : 51( 6.8%); C : 3( 0.4%); D--: 1( 0.1%); E--: 1( 0.1%); F--: 2( 0.3%)

G--: 9( 1.2%); H- : 3( 0.4%); I- : 11( 1.5%); K++:144(19.2%); L--: 13( 1.7%)

M--: 2( 0.3%); N- : 9( 1.2%); P++:181(24.1%); Q--: 2( 0.3%); R--: 4( 0.5%)

S+ : 90(12.0%); T+ : 78(10.4%); V--: 4( 0.5%); W : 0( 0.0%); Y++:143(19.0%)

Mgfp3 v1

COMPOSITIONAL ANALYSIS

A : 5( 7.1%); C : 0( 0.0%); D : 2( 2.9%); E : 0( 0.0%); F : 1( 1.4%)

G : 13(18.6%); H : 0( 0.0%); I : 2( 2.9%); K : 4( 5.7%); L : 3( 4.3%)

M : 1( 1.4%); N : 6( 8.6%); P : 3( 4.3%); Q : 1( 1.4%); R : 8(11.4%)

S : 3( 4.3%); T : 0( 0.0%); V : 5( 7.1%); W : 3( 4.3%); Y : 10(14.3%)

Mgfp3 v2

COMPOSITIONAL ANALYSIS

A : 4( 5.2%); C : 0( 0.0%); D : 2( 2.6%); E : 0( 0.0%); F : 1( 1.3%)

G : 15(19.5%); H : 0( 0.0%); I : 2( 2.6%); K : 2( 2.6%); L : 3( 3.9%)

M : 1( 1.3%); N : 8(10.4%); P : 2( 2.6%); Q : 1( 1.3%); R : 10(13.0%)

S : 6( 7.8%); T : 0( 0.0%); V : 5( 6.5%); W : 4( 5.2%); Y : 11(14.3%)

***Mytilus unguiculatus***

Mufp2

COMPOSITIONAL ANALYSIS

A- : 6( 2.1%); C++: 38(13.4%); D : 11( 3.9%); E- : 6( 2.1%); F : 5( 1.8%)

G+ : 34(12.0%); H : 2( 0.7%); I- : 3( 1.1%); K++: 40(14.1%); L- : 13( 4.6%)

M- : 1( 0.4%); N : 21( 7.4%); P+ : 28( 9.9%); Q- : 3( 1.1%); R : 11( 3.9%)

S : 14( 4.9%); T : 14( 4.9%); V : 10( 3.5%); W : 1( 0.4%); Y++: 23( 8.1%)

Mufp3

COMPOSITIONAL ANALYSIS

A : 4( 5.2%); C : 0( 0.0%); D : 1( 1.3%); E : 0( 0.0%); F : 2( 2.6%)

G : 16(20.8%); H : 1( 1.3%); I : 1( 1.3%); K : 3( 3.9%); L : 7( 9.1%)

M : 1( 1.3%); N : 9(11.7%); P : 3( 3.9%); Q : 1( 1.3%); R : 1( 1.3%)

S : 4( 5.2%); T : 0( 0.0%); V : 5( 6.5%); W : 4( 5.2%); Y : 14(18.2%)

Mufp3 v1

COMPOSITIONAL ANALYSIS

A : 5( 6.4%); C : 0( 0.0%); D : 1( 1.3%); E : 0( 0.0%); F : 1( 1.3%)

G : 16(20.5%); H : 1( 1.3%); I : 1( 1.3%); K : 2( 2.6%); L : 8(10.3%)

M : 1( 1.3%); N : 10(12.8%); P : 3( 3.8%); Q : 1( 1.3%); R : 2( 2.6%)

S : 4( 5.1%); T : 0( 0.0%); V : 4( 5.1%); W : 3( 3.8%); Y : 15(19.2%)

Mufp3 v2

COMPOSITIONAL ANALYSIS

A : 5( 6.4%); C : 1( 1.3%); D : 1( 1.3%); E : 0( 0.0%); F : 0( 0.0%)

G : 16(20.5%); H : 1( 1.3%); I : 1( 1.3%); K : 2( 2.6%); L : 8(10.3%)

M : 1( 1.3%); N : 10(12.8%); P : 3( 3.8%); Q : 1( 1.3%); R : 1( 1.3%)

S : 4( 5.1%); T : 0( 0.0%); V : 4( 5.1%); W : 4( 5.1%); Y : 15(19.2%)

Mufp3 v3

COMPOSITIONAL ANALYSIS (extremes relative to: swp23s.q)

A : 4( 6.1%); C : 0( 0.0%); D : 1( 1.5%); E : 0( 0.0%); F : 1( 1.5%)

G : 10(15.2%); H : 0( 0.0%); I : 2( 3.0%); K : 4( 6.1%); L : 5( 7.6%)

M : 1( 1.5%); N : 9(13.6%); P : 3( 4.5%); Q : 1( 1.5%); R : 7(10.6%)

S : 3( 4.5%); T : 0( 0.0%); V : 3( 4.5%); W : 3( 4.5%); Y : 9(13.6%)

Mufp3 v4

COMPOSITIONAL ANALYSIS

A : 5( 6.4%); C : 0( 0.0%); D : 1( 1.3%); E : 0( 0.0%); F : 1( 1.3%)

G : 16(20.5%); H : 1( 1.3%); I : 1( 1.3%); K : 2( 2.6%); L : 7( 9.0%)

M : 1( 1.3%); N : 10(12.8%); P : 4( 5.1%); Q : 1( 1.3%); R : 1( 1.3%)

S : 4( 5.1%); T : 0( 0.0%); V : 4( 5.1%); W : 4( 5.1%); Y : 15(19.2%)

Mufp3 v5

COMPOSITIONAL ANALYSIS

A : 5( 6.4%); C : 1( 1.3%); D : 1( 1.3%); E : 0( 0.0%); F : 1( 1.3%)

G : 16(20.5%); H : 1( 1.3%); I : 1( 1.3%); K : 2( 2.6%); L : 8(10.3%)

M : 1( 1.3%); N : 10(12.8%); P : 3( 3.8%); Q : 1( 1.3%); R : 1( 1.3%)

S : 4( 5.1%); T : 0( 0.0%); V : 4( 5.1%); W : 4( 5.1%); Y : 14(17.9%)

Mufp3 v6

COMPOSITIONAL ANALYSIS

A : 4( 5.2%); C : 0( 0.0%); D : 2( 2.6%); E : 0( 0.0%); F : 1( 1.3%)

G : 15(19.5%); H : 1( 1.3%); I : 2( 2.6%); K : 1( 1.3%); L : 8(10.4%)

M : 1( 1.3%); N : 9(11.7%); P : 3( 3.9%); Q : 2( 2.6%); R : 2( 2.6%)

S : 5( 6.5%); T : 0( 0.0%); V : 3( 3.9%); W : 3( 3.9%); Y : 15(19.5%)

Mufp3 v7

COMPOSITIONAL ANALYSIS

A : 5( 6.4%); C : 0( 0.0%); D : 1( 1.3%); E : 1( 1.3%); F : 1( 1.3%)

G : 16(20.5%); H : 2( 2.6%); I : 2( 2.6%); K : 2( 2.6%); L : 8(10.3%)

M : 1( 1.3%); N : 10(12.8%); P : 3( 3.8%); Q : 1( 1.3%); R : 1( 1.3%)

S : 4( 5.1%); T : 0( 0.0%); V : 3( 3.8%); W : 4( 5.1%); Y : 13(16.7%)

Mufp3 v8

OMPOSITIONAL ANALYSIS

A : 3( 3.8%); C : 0( 0.0%); D : 1( 1.3%); E : 1( 1.3%); F : 4( 5.1%)

G : 17(21.8%); H : 1( 1.3%); I : 2( 2.6%); K : 4( 5.1%); L : 7( 9.0%)

M : 1( 1.3%); N : 8(10.3%); P : 6( 7.7%); Q : 1( 1.3%); R : 1( 1.3%)

S : 4( 5.1%); T : 0( 0.0%); V : 3( 3.8%); W : 3( 3.8%); Y : 11(14.1%)

Mufp3 v9

COMPOSITIONAL ANALYSIS

A : 4( 5.1%); C : 0( 0.0%); D : 1( 1.3%); E : 1( 1.3%); F : 1( 1.3%)

G : 16(20.5%); H : 1( 1.3%); I : 2( 2.6%); K : 2( 2.6%); L : 8(10.3%)

M : 1( 1.3%); N : 10(12.8%); P : 3( 3.8%); Q : 1( 1.3%); R : 1( 1.3%)

S : 4( 5.1%); T : 0( 0.0%); V : 4( 5.1%); W : 4( 5.1%); Y : 14(17.9%)

Mufp3 v10

COMPOSITIONAL ANALYSIS

A : 5( 6.4%); C : 1( 1.3%); D : 1( 1.3%); E : 0( 0.0%); F : 5( 6.4%)

G : 16(20.5%); H : 0( 0.0%); I : 1( 1.3%); K : 4( 5.1%); L : 8(10.3%)

M : 1( 1.3%); N : 8(10.3%); P : 4( 5.1%); Q : 1( 1.3%); R : 1( 1.3%)

S : 4( 5.1%); T : 0( 0.0%); V : 4( 5.1%); W : 4( 5.1%); Y : 10(12.8%)

Mufp3 v11

COMPOSITIONAL ANALYSIS

A : 4( 5.0%); C : 0( 0.0%); D : 1( 1.2%); E : 1( 1.2%); F : 4( 5.0%)

G : 16(20.0%); H : 1( 1.2%); I : 2( 2.5%); K : 3( 3.8%); L : 8(10.0%)

M : 1( 1.2%); N : 9(11.2%); P : 3( 3.8%); Q : 1( 1.2%); R : 1( 1.2%)

S : 5( 6.2%); T : 0( 0.0%); V : 3( 3.8%); W : 4( 5.0%); Y : 13(16.2%)

Mufp3 v12

COMPOSITIONAL ANALYSIS

A : 5( 6.4%); C : 0( 0.0%); D : 1( 1.3%); E : 1( 1.3%); F : 1( 1.3%)

G : 16(20.5%); H : 1( 1.3%); I : 2( 2.6%); K : 2( 2.6%); L : 8(10.3%)

M : 1( 1.3%); N : 10(12.8%); P : 3( 3.8%); Q : 1( 1.3%); R : 1( 1.3%)

S : 4( 5.1%); T : 0( 0.0%); V : 3( 3.8%); W : 4( 5.1%); Y : 14(17.9%)

Mufp3 v13

COMPOSITIONAL ANALYSIS

A : 5( 6.2%); C : 0( 0.0%); D : 1( 1.2%); E : 0( 0.0%); F : 12(15.0%)

G : 18(22.5%); H : 0( 0.0%); I : 1( 1.2%); K : 4( 5.0%); L : 8(10.0%)

M : 1( 1.2%); N : 8(10.0%); P : 4( 5.0%); Q : 1( 1.2%); R : 1( 1.2%)

S : 4( 5.0%); T : 0( 0.0%); V : 4( 5.0%); W : 2( 2.5%); Y : 6( 7.5%)

Mufp3 v14

COMPOSITIONAL ANALYSIS

A : 4( 5.6%); C : 0( 0.0%); D : 1( 1.4%); E : 0( 0.0%); F : 1( 1.4%)

G : 15(20.8%); H : 0( 0.0%); I : 2( 2.8%); K : 6( 8.3%); L : 4( 5.6%)

M : 1( 1.4%); N : 7( 9.7%); P : 3( 4.2%); Q : 1( 1.4%); R : 5( 6.9%)

S : 3( 4.2%); T : 0( 0.0%); V : 4( 5.6%); W : 4( 5.6%); Y : 11(15.3%)

Mufp6

COMPOSITIONAL ANALYSIS

A : 4( 3.3%); C : 12( 9.8%); D : 4( 3.3%); E : 1( 0.8%); F : 4( 3.3%)

G : 15(12.3%); H : 0( 0.0%); I : 4( 3.3%); K : 12( 9.8%); L : 5( 4.1%)

M : 2( 1.6%); N : 13(10.7%); P : 3( 2.5%); Q : 2( 1.6%); R : 4( 3.3%)

S : 7( 5.7%); T : 3( 2.5%); V : 3( 2.5%); W : 0( 0.0%); Y : 24(19.7%)

Mufp6 v1

COMPOSITIONAL ANALYSIS

A : 3( 2.4%); C : 12( 9.8%); D : 6( 4.9%); E : 0( 0.0%); F : 5( 4.1%)

G : 15(12.2%); H : 0( 0.0%); I : 3( 2.4%); K : 11( 8.9%); L : 4( 3.3%)

M : 3( 2.4%); N : 10( 8.1%); P : 4( 3.3%); Q : 1( 0.8%); R : 7( 5.7%)

S : 8( 6.5%); T : 3( 2.4%); V : 4( 3.3%); W : 0( 0.0%); Y : 24(19.5%)

Mufp6 v2

COMPOSITIONAL ANALYSIS

A : 3( 2.4%); C : 12( 9.8%); D : 5( 4.1%); E : 0( 0.0%); F : 4( 3.3%)

G : 16(13.0%); H : 0( 0.0%); I : 4( 3.3%); K : 9( 7.3%); L : 4( 3.3%)

M : 2( 1.6%); N : 12( 9.8%); P : 4( 3.3%); Q : 1( 0.8%); R : 8( 6.5%)

S : 7( 5.7%); T : 3( 2.4%); V : 3( 2.4%); W : 0( 0.0%); Y : 26(21.1%)

Mufp6 v3

COMPOSITIONAL ANALYSIS

A : 3( 2.4%); C : 12( 9.8%); D : 6( 4.9%); E : 0( 0.0%); F : 5( 4.1%)

G : 15(12.2%); H : 0( 0.0%); I : 4( 3.3%); K : 10( 8.1%); L : 4( 3.3%)

M : 2( 1.6%); N : 11( 8.9%); P : 4( 3.3%); Q : 1( 0.8%); R : 7( 5.7%)

S : 7( 5.7%); T : 4( 3.3%); V : 4( 3.3%); W : 0( 0.0%); Y : 24(19.5%)

Mufp6 v4

COMPOSITIONAL ANALYSIS

A : 3( 2.4%); C : 12( 9.8%); D : 6( 4.9%); E : 0( 0.0%); F : 4( 3.3%)

G : 15(12.2%); H : 0( 0.0%); I : 3( 2.4%); K : 11( 8.9%); L : 4( 3.3%)

M : 3( 2.4%); N : 10( 8.1%); P : 3( 2.4%); Q : 1( 0.8%); R : 8( 6.5%)

S : 7( 5.7%); T : 4( 3.3%); V : 4( 3.3%); W : 0( 0.0%); Y : 25(20.3%)

Mufp6 v5

COMPOSITIONAL ANALYSIS

A : 3( 2.4%); C : 12( 9.8%); D : 5( 4.1%); E : 1( 0.8%); F : 4( 3.3%)

G : 15(12.2%); H : 0( 0.0%); I : 3( 2.4%); K : 11( 8.9%); L : 4( 3.3%)

M : 3( 2.4%); N : 10( 8.1%); P : 4( 3.3%); Q : 1( 0.8%); R : 7( 5.7%)

S : 7( 5.7%); T : 4( 3.3%); V : 4( 3.3%); W : 0( 0.0%); Y : 25(20.3%)

Mufp6 v6

COMPOSITIONAL ANALYSIS

A : 3( 2.4%); C : 12( 9.8%); D : 6( 4.9%); E : 0( 0.0%); F : 8( 6.5%)

G : 15(12.2%); H : 0( 0.0%); I : 3( 2.4%); K : 10( 8.1%); L : 2( 1.6%)

M : 3( 2.4%); N : 11( 8.9%); P : 4( 3.3%); Q : 1( 0.8%); R : 7( 5.7%)

S : 6( 4.9%); T : 4( 3.3%); V : 4( 3.3%); W : 0( 0.0%); Y : 24(19.5%)

Mufp6 v7

COMPOSITIONAL ANALYSIS

A : 3( 2.9%); C : 9( 8.8%); D : 5( 4.9%); E : 0( 0.0%); F : 4( 3.9%)

G : 12(11.8%); H : 0( 0.0%); I : 3( 2.9%); K : 9( 8.8%); L : 3( 2.9%)

M : 3( 2.9%); N : 7( 6.9%); P : 4( 3.9%); Q : 1( 1.0%); R : 6( 5.9%)

S : 6( 5.9%); T : 3( 2.9%); V : 4( 3.9%); W : 0( 0.0%); Y : 20(19.6%)

Mufp6 v8

COMPOSITIONAL ANALYSIS

A : 3( 3.1%); C : 9( 9.3%); D : 5( 5.2%); E : 0( 0.0%); F : 3( 3.1%)

G : 12(12.4%); H : 0( 0.0%); I : 3( 3.1%); K : 8( 8.2%); L : 3( 3.1%)

M : 1( 1.0%); N : 7( 7.2%); P : 4( 4.1%); Q : 0( 0.0%); R : 6( 6.2%)

S : 4( 4.1%); T : 4( 4.1%); V : 4( 4.1%); W : 0( 0.0%); Y : 21(21.6%)

Mufp6 v9

COMPOSITIONAL ANALYSIS

A : 3( 3.1%); C : 9( 9.4%); D : 5( 5.2%); E : 1( 1.0%); F : 2( 2.1%)

G : 12(12.5%); H : 0( 0.0%); I : 3( 3.1%); K : 10(10.4%); L : 3( 3.1%)

M : 1( 1.0%); N : 11(11.5%); P : 3( 3.1%); Q : 0( 0.0%); R : 3( 3.1%)

S : 6( 6.2%); T : 3( 3.1%); V : 1( 1.0%); W : 0( 0.0%); Y : 20(20.8%)

***Mizuhopecten yessoensis***

Myfp1 v1

COMPOSITIONAL ANALYSIS

A--: 4( 0.8%); C : 0( 0.0%); D++: 48( 9.5%); E--: 5( 1.0%); F--: 1( 0.2%)

G--: 2( 0.4%); H : 5( 1.0%); I--: 5( 1.0%); K : 19( 3.8%); L--: 3( 0.6%)

M : 5( 1.0%); N+ : 50( 9.9%); P : 34( 6.7%); Q++: 90(17.8%); R : 25( 5.0%)

S- : 17( 3.4%); T++:189(37.4%); V--: 0( 0.0%); W : 1( 0.2%); Y--: 2( 0.4%)

Myfp1 v2

COMPOSITIONAL ANALYSIS

A--: 3( 0.9%); C : 1( 0.3%); D : 18( 5.6%); E--: 3( 0.9%); F- : 2( 0.6%)

G++: 87(26.9%); H- : 1( 0.3%); I--: 2( 0.6%); K- : 3( 0.9%); L : 33(10.2%)

M- : 2( 0.6%); N--: 0( 0.0%); P : 16( 4.9%); Q+ : 28( 8.6%); R : 20( 6.2%)

S : 29( 9.0%); T+ : 30( 9.3%); V : 15( 4.6%); W++: 18( 5.6%); Y : 13( 4.0%)

***Perna canaliculus***

Pcfp1 v1

COMPOSITIONAL ANALYSIS

A--: 4( 0.9%); C : 15( 3.4%); D--: 2( 0.5%); E--: 0( 0.0%); F--: 2( 0.5%)

G- : 12( 2.8%); H- : 1( 0.2%); I--: 4( 0.9%); K++:102(23.4%); L--: 7( 1.6%)

M--: 1( 0.2%); N : 9( 2.1%); P++: 92(21.1%); Q--: 1( 0.2%); R--: 1( 0.2%)

S--: 9( 2.1%); T--: 3( 0.7%); V++: 83(19.0%); W : 0( 0.0%); Y++: 88(20.2%)

Pcfp1 v2

COMPOSITIONAL ANALYSIS

A--: 4( 0.9%); C : 15( 3.5%); D--: 2( 0.5%); E--: 0( 0.0%); F--: 2( 0.5%)

G- : 12( 2.8%); H- : 1( 0.2%); I--: 4( 0.9%); K++:101(23.4%); L--: 7( 1.6%)

M--: 1( 0.2%); N : 9( 2.1%); P++: 91(21.1%); Q--: 1( 0.2%); R--: 1( 0.2%)

S--: 9( 2.1%); T--: 3( 0.7%); V++: 82(19.0%); W : 0( 0.0%); Y++: 87(20.1%)

Pcfp1 v3

COMPOSITIONAL ANALYSIS

A--: 2( 0.5%); C : 9( 2.2%); D--: 1( 0.2%); E--: 0( 0.0%); F--: 2( 0.5%)

G--: 6( 1.5%); H- : 1( 0.2%); I--: 1( 0.2%); K++: 98(24.3%); L--: 6( 1.5%)

M--: 1( 0.2%); N- : 5( 1.2%); P++: 91(22.5%); Q--: 1( 0.2%); R--: 1( 0.2%)

S--: 8( 2.0%); T--: 3( 0.7%); V++: 83(20.5%); W : 0( 0.0%); Y++: 85(21.0%)

Pcfp1 v4

COMPOSITIONAL ANALYSIS

A--: 2( 0.5%); C : 9( 2.3%); D--: 1( 0.3%); E--: 0( 0.0%); F--: 2( 0.5%)

G--: 6( 1.5%); H- : 1( 0.3%); I--: 1( 0.3%); K++: 94(24.2%); L--: 6( 1.5%)

M--: 1( 0.3%); N- : 5( 1.3%); P++: 87(22.4%); Q--: 1( 0.3%); R--: 1( 0.3%)

S--: 8( 2.1%); T--: 3( 0.8%); V++: 79(20.4%); W : 0( 0.0%); Y++: 81(20.9%)

***Perna viridis***

Pvfp1 v1

COMPOSITIONAL ANALYSIS

A++:105(18.7%); C : 0( 0.0%); D--: 1( 0.2%); E--: 3( 0.5%); F--: 2( 0.4%)

G : 30( 5.3%); H : 9( 1.6%); I- : 7( 1.2%); K++: 79(14.1%); L--: 4( 0.7%)

M- : 2( 0.4%); N- : 5( 0.9%); P++:110(19.6%); Q--: 0( 0.0%); R- : 8( 1.4%)

S--: 6( 1.1%); T++: 80(14.3%); V--: 12( 2.1%); W++: 91(16.2%); Y : 7( 1.2%)

Pvfp1 v2

COMPOSITIONAL ANALYSIS

A++: 80(18.6%); C : 0( 0.0%); D--: 1( 0.2%); E--: 3( 0.7%); F--: 2( 0.5%)

G : 30( 7.0%); H : 7( 1.6%); I- : 6( 1.4%); K+ : 56(13.0%); L--: 4( 0.9%)

M- : 2( 0.5%); N- : 5( 1.2%); P++: 84(19.5%); Q--: 0( 0.0%); R : 9( 2.1%)

S--: 4( 0.9%); T++: 58(13.5%); V--: 9( 2.1%); W++: 64(14.8%); Y : 7( 1.6%)

Pvfp3

COMPOSITIONAL ANALYSIS

A : 2( 2.9%); C : 11(15.7%); D : 4( 5.7%); E : 1( 1.4%); F : 6( 8.6%)

G : 5( 7.1%); H : 0( 0.0%); I : 4( 5.7%); K : 1( 1.4%); L : 4( 5.7%)

M : 2( 2.9%); N : 6( 8.6%); P : 2( 2.9%); Q : 2( 2.9%); R : 4( 5.7%)

S : 5( 7.1%); T : 3( 4.3%); V : 4( 5.7%); W : 1( 1.4%); Y : 3( 4.3%)

Pvfp5

COMPOSITIONAL ANALYSIS

A : 4( 2.3%); C : 29(16.5%); D : 3( 1.7%); E : 0( 0.0%); F : 4( 2.3%)

G : 25(14.2%); H : 0( 0.0%); I : 2( 1.1%); K : 23(13.1%); L : 7( 4.0%)

M : 2( 1.1%); N : 9( 5.1%); P : 9( 5.1%); Q : 4( 2.3%); R : 8( 4.5%)

S : 10( 5.7%); T : 5( 2.8%); V : 7( 4.0%); W : 0( 0.0%); Y : 25(14.2%)

Pvfp6

COMPOSITIONAL ANALYSIS (extremes relative to: swp23s.q)

A : 5( 4.1%); C : 17(13.9%); D : 5( 4.1%); E : 3( 2.5%); F : 7( 5.7%)

G : 13(10.7%); H : 1( 0.8%); I : 7( 5.7%); K : 5( 4.1%); L : 3( 2.5%)

M : 3( 2.5%); N : 6( 4.9%); P : 12( 9.8%); Q : 5( 4.1%); R : 3( 2.5%)

S : 5( 4.1%); T : 5( 4.1%); V : 9( 7.4%); W : 0( 0.0%); Y : 8( 6.6%)

**Section 5 (S5): Metal ion ligand binding sites of Mfps - Predicted binding residue (s)**

***Atrina pectinata* foot protein (Apfp1),**

Zn^2+^ binding site : D35 K92 D104 D112 K114 K137 D155 E160 K163 P164 G165 N167 G168 K171 G174 H175 G176 Y177 P179 S180 S181 Y182 G183 G185 Y186 Y191 Y192 D202 D204 A208 T209 G210 G211 C212 H214 G215 Y219 C223 Y226 E235 C240 M243 I249 N251 E255 D278 S284 H285 W289 K293 C296 W301 E305 K307 K314 H315 C316 A318 K328 V329 C332 C340 E341 P347 C348 C350 Y352 ,

Ca^2+^ binding site: I249 E255 E341.

Na^+^ binding site: Y162 K163 G166 N167 K171 G173 F178 P179 Y191 Y196 P218 Y219 G220 D278 E305 K307 Y308 G312 K314 H315 C316 V329.

No binding site detected for the following ions:( Cu^2+^, Fe^2+^, Fe^3+^, Mg^2+^, Mn^2+^, K^+^, CO^2-^_3_, NO^-^_2_, SO^2-^_4_, PO^3-^_4_)

***Dreissena polymorpha* foot protein (Dpfp1),**

Zn^2+^ binding site: C8 Y67 D92 E102 K106 E109 K130 Y134 Y141 Y155 H172 Y176 R179 Y183 Q207 E209 Y210 H211 Y223 E227 K230 Y234 D237 D241 P244 D248 Y249 D250 D267 K269 P275 D280 Y286 D289 D306 D319 N321 D326 D328 D341 N354 D367 D393,

Ca^2+^ binding site: P174 N243 D267 K269 D289 D293 G316 P317 P402 Y403,

Na^+^ binding site: C8,

K^+^ binding site: D267 P272 I276,

No binding site detected for the following ions:(Cu^2+^, Fe^2+^, Fe^3+^, Mg^2+^, Mn^2+^, CO^2-^_3_, NO^-^_2_, SO^2-^_4_, PO^3-^_4_).

***Mytilus californianus* (Mcfp)**

**Mcfp1 v1**

Zn^2+^ binding site: Y22 E28 S30 Y32 Y40 E42 A44 S46 H48 H50 H52 H54 H56 H57 L58 H60 H62 H64 H66 V68 H70 H72 H74 H76 V78 H80 I82 H84 H86 V88 H90 H92 H94 H96 V98 H100 H102 H104 H106 V108 H11 0 H112 H114 H116 V118 H120 H122 H124 H126 N127 V128 H130 H132 H134 H136 V138 H140 H142 H144 H146 V148 H150 H152 H154 H156 V158 H160 H162 H164 H166 V168 H170 H172 H174 H176 V178 H180 H182 H184 H186 V188 H190 H192 H194 H196 V198 H200 H202 H204 H206 V208 H210 H212 H214 H216 V218 H220 H222 H224 H226 V228 H230 K231 H232 H234 H236 V238 H240 H242 H244 H246 V248 H250 H252 H254 H256 V258 H260 H262 H264 H266 V268 H270 H272 H274 H276 V278 L279 H280 H282 H284 H286 V288 H290.

Ca^2+^ binding site: D458 E461.

Na^+^ binding site: Y32 G36 S46 H48 H50 H52 H54 H56 H60 H62 H66 H70 H72 H74 H76 H80 I82 H84 H86 H90 H92 H94 H96 H100 H102 H104 H106 V108 H110 H112 H114 H116 H120 H122 H124 H126 V128 H130 H132 H136 V138 H140 H142 H146 V148 H150 H152 H154 K155 H156 V158 H160 H162 H164 H166 H170 H172 H176 V178 H180 H182 H184 H186 V188 H190 H192 H194 H196 H200 H202 H204 H206 V208 H210 H212 H214 H216 H220 H222 H224 H226 H230 H232 H234 K235 H236 V238 L239 H240 H242 H244 H246 V248 H250 H252 H254 H256 V258 H260 H262 H264 H266 H270 H272 H274 H276 L279 H280 H282 H284 K285 H286 L289 H290 H292 H294 K295 H296 V298 H300 H302 H304 H306 H310 H312 H314 K315 H316 V318 E319 H320 H322 H326 H330 H332 H334 H336 V338 H340 S341 H342 H344 H346 V348 H350 H352 H356 H360 H362 H364 H366 H370 H372 H374 H376 H380 H382 H384 H386 H390 H392 H394 S395 H396 A399 H407 F409 R423 I424 R425 H426 H292 H294 K295 H296 H297 V298 H300 H302 H304 H306 V308 H310 H312 H314 H316 V318 E319 H320 H322 H324 H326 H327 V328 H330 H332 H334 H336 H337 V338 V339 H340 H342 H344 H346 V348 H350 H352 H354 H356 V358 H360 H362 H364 H366 I368 H370 H372 H374 H376 V378 H380 H382 H384 H386 I388 H390 H392 H394 H396 A399 H401 H403 H405 H407 E410 G411 N414 D415 D419 H426 Y430 N434 R437 G440 C452 E454 Y456 S459 C462 H470 F472 Q476 H478 H479 D490 D503 D507 D515 D526 D532 D543 H545 H554 H555 D559 D566 Y576 D577 H578 D587 D588 H589 D599 H600 D609 D610 D620 D621 H622 N624 D625 H644 H666 H673 H710 E711 D721 K728 Q735 D760 Q761 E766.

K^+^ binding site: M75 S79 I82 M83 H86 L99 H102 V103 V108.

No binding site detected for the following ions:( Cu^2+^, Fe^2+^, Fe^3+^, Mg^2+^, Mn^2+^, CO^2-^3, NO^-^_2_, SO^2-^_4_, PO^3-^_4_).

**Mcfp1 v2,**

Zn^2+^ binding site: Y22 E28 S30 Y32 I35 H37 Y40 E42 S46 H48 H50 H52 H54 H56 H57 L58 H60 H62 H64 H66 V68 H70 H72 H74 H76 V78 H80 I82 H84 H86 V88 H90 H92 H94 H96 V98 H100 H102 H104 H106 V108 H110 H112 H114 H116 V118 H120 H122 H124 H126 V128 H130 H132 H134 H136 V138 H140 H142 H144 H146 S147 V148 H150 H152 H154 H156 V158 H160 H162 H164 H166 N167 V168 H170 H172 H174 H176 V178 H180 H182 H184 H186 V188 H190 H192 H194 H196 V198 H200 H202 H204 H206 V208 H210 H212 H214 H216 V218 H220 H222 H224 H226 V228 H230 H232 H234 H236 V238 H240 H242 H24 4 H246 V248 H250 H252 H254 H256 V258 H260 H262 H264 H266 V268 H270 H272 H274 H276 V278 H280 H282 H284 H286 V288 H290 H292 H294 H296 V298 H300 H302 H304 H306 V308 H310 H312 H314 M315 H316 V318 L319 H320 H322 H324 H326 V328 H330 H332 H334 H336 H337 V338 V339 H340 H342 H344 H346 V348 H350 K351 H352 H354 H356 V358 E359 H360 H362 H364 H366 H367 V368 H370 H372 H374 H376 H377 V378 V379 H380 H382 H384 H386 V388 V389 H390 H392 H394 H396 V398 H400 H402 H404 H406 I408 H410 H412 H414 H416 V418 H420 H422 H424 H426 I428 H430 H432 H434 H436 A439 H441 H443 H445 H447 E450 G451 D455 D456 G457 D459 N461 H466 Y470 R477 G480 G481 R483 C492 E494 Y496 D498 H510 H518 H519 T521 S522 H532 D543 H544 D547 D566 Q591 H595 H596 D606 D610 D617 D627 D638 D639 H651 D661 H662 H706 H713 H739 D761 N787 K794 D800 Q801.

Cu^2+^ binding site: H150 H152.

Ca^2+^ binding site: D558 D561.

Mn^2+^ binding site: H212 Q257 H260 H262.

Na^+^ binding site: E42 A44 H50 H52 H54 H56 L58 H60 H62 H66 H70 H72 H74 H76 H80 I82 H86 H90 H92 H96 V98 H100 H102 H106 V108 H110 H112 H116 H120 H122 H126 L129 H130 H132 H136 V138 H140 H142 H144 H146 V148 H150 H152 H154 H156 V158 H160 H162 H164 H166 V168 H170 H172 H174 H176 H180 H182 H186 V188 H190 H192 H194 H196 V198 H200 H202 H206 V208 H210 H212 H214 N215 H216 H220 H222 H224 K225 H226 V228 L229 H230 H232 H234 T235 H236 V238 H240 H242 H246 V248 H250 H252 H254 H256 H260 H262 H266 V268 H270 H272 H274 H276 V278 H280 H282 H284 H286 V288 L289 H290 H292 H294 H296 V298 H300 H302 H306 V308 L309 H310 H312 H314 M315 H316 H320 K321 H322 H324 K325 H326 V328 H330 H332 H334 K335 H336 V338 H340 H342 H344 H346 H350 H352 H354 H356 H360 H362 H364 H366 H370 H372 H376 V378 H380 H382 H384 H386 V388 H390 H392 H394 H396 V398 H400 H402 H406 H410 H412 H414 H416 H420 H422 H424 H426 H430 H432 H436 A439 V44 0 H441 D455 D456 G457 D459 N461.

K^+^ binding site: H50 S51.

No binding site detected for the following ions:( Fe^2+^, Fe^3+^, Mg^2+^, CO^2-^_3_, NO^-^_2_, SO^2-^_4_, PO^3-^_4_).

**Mcfp2**

Zn^2+^ binding site: L8 C11 Q13 C15 L16 G17 T18 P20 Y23 E24 D25 D26 E27 D28 D29 K31 P32 T34 A35 K37 P41 K42 Y43 V46 P48 C49 K52 C54 K55 Y56 N57 Q59 C60 S65 S66 K68 C69 C71 G74 Y75 C80 N81 K83 N84 A85 C86 N89 P90 C91 K92 N93 K94 K96 C97 K102 T103 K105 C106 C108 G111 N112 L116 C117 E118 K119 V121 C122 N125 P126 C127 K128 N129 R130 C133 W136 K142 C143 C145 G148 Y149 G151 R153 C154 H157 V158 C159 N162 P163 C164 K165 N166 K167 G168 C170 D173 T176 K179 C180 C182 G185 Y186 C191 E193 N194 A195 C196 N199 P200 C201 S202 N203 G204 G205 C207 D210 G213 D214 T216 C217 D218 C219 G222 Y223 E227 C228 E229 K230 Y231 V232 C233 N236 C238 R239 N240 N241 G242 C244 S245 D247 G250 K253 C254 C256 G259 Y260 G262 T264 C265 H268 V269 C270 N273 C275 E276 N277 R278 C281 N286 K289 C290 C292 G295 Y296 T300 C301 E303 N304 V305 C306 N309 C311 Q312 N313 R314 G315 C317 P319 D324 K327 C328 C330 G333 Y334 G336 C339 E340 K342 P343 N344 P345 C346 K349 C351 K352 N353 G354 C357 Y359 T365 C366 C368 G371 Y372 H376 C377 D379 A381 Y382 P384 N385 P386 C387 R390 C392 K393 N394 G395 C398 K401 N403 C407 C409 G412 Y413 C418 A419 S425 E429 E430,

Ca^2+^ binding site: L6 P20 P21 P45 V46 P48 P62 N63 S66 C97 P99 K105 N120 C122 P124 P126 T139 C145 C170 D173 C180 C182 G185 S187 N194 C196 N203 G205 K206 C207 S208 D210 F212 D214 C217 E229 A234 N236 P237 R239 N241 S245 S246 D247 G248 G250 Y252 C254 C256 G259 Y260 S261 P263 H268 C270 N273 P274 N304 C311 N313 G315 C317 N321 D323 C328 G333 Y334 S335 P337 C339 K342 N344 C346 S348 C351 K352 N353 K356 C357 Y359 C366 C368 A369 G371 R373 G374 R375 N385 C387 R390 K393 N394 G412 S414 K416 C418 P423,

Na^+^ binding site: C11 Q13 C15 L16 G17 T18 A19 P20 P21 Q22 E24 D25 D33 K37 S39 P40 N47 K52 P53 C54 K55 Y56 N57 G58 N84 N89 P90 C91 K92 N93 K94 S95 E118 C122 S123 N125 P126 C127 K128 N129 R130 E155 H157 N162 P163 C164 K165 N166 K167 G168 K175 Q192 N194 C196 I197 N199 P200 C201 S202 N203 G204 G205 E229 C233 N236 P237 C238 R239 N240 N241 G242 N266 H268 C275 E276 N277 R278 G279 N309 Q312 N313 R314 C317 D323 N344 P350 C351 K352 N353 G354 G355 T378 K383 N385 P386 A388 P391 C392 K393 N394 G395 G396 P423,

SO^2-^_4_ binding site: K206 R220 P221,

No binding site detected for the following ions:( Cu^2+^, Fe^2+^, Fe^3+^, Mg^2+^, Mn^2+^, K^+^, CO^2-^_3_, NO^-^_2_, PO^3-^_4_).

**Mcfp3 v1**

Zn^2+^ binding site: Q21 D23 Y28 Y38 K39 N43 Y45 R47 Y50 W56 W61,

Ca^2+^ binding site: L12 I15 G26 N27 G48 Y50 G51,

Mg^2+^ binding site: R60 W61,

CO^2-^_3_ binding site: L12 V13 I15 R63,

SO^2-^_4_ binding site: D23 R47 W52 W56 K57 K58 G59 R60 W61 K64 Y65,

No binding site detected for the following ions:( Cu^2+^, Fe^2+^, Fe^3+^, Mn^2+^, Na^+^, K^+^, NO^-^_2_, PO^3-^_4_).

**Mcfp3 v2**

Zn^2+^ binding site: Q21 D23 K28 Y34 Y38 G39 Y42 Y48 R50 Y52 W54 K56 W58 W63,

Ca^2+^ binding site: G16 S22 G61,

Mg^2+^ binding site: Y27 K41,

Na^+^ binding site: Y48,

K^+^ binding site: K2 S3 S5 I6 L9,

No binding site detected for the following ions:( Cu^2+^, Fe^2+^, Fe^3+^, Mn^2+^, CO^2-^_3_, NO^-^_2_, SO^2-^_4_, PO^3-^_4_).

**Mcfp3 v3**

Zn^2+^ binding site: K3 Q21 D23 Y26 Y28 Y38 N39 Y42 Y45 N46 G47 Y48 Y51 H52 Y55 G56 W57 K59 W61 N62 W66 Y70 Y71,

Ca^2+^ binding site: G56 W57 N58 G60 W61 N62,

Na^+^ binding site: Y42 Y48 Y51.

No binding site detected for the following ions:( Cu^2+^, Fe^2+^, Fe^3+^, Mg^2+^, Mn^2+^, K^+^, CO^2-^_3_, NO^-^_2_, SO^2-^_4_, PO^3-^_4_).

**Mcfp3 v4**

Zn^2+^ binding site: K3 Q21 D23 D27 Y28 Y38 N39 Y45 Y48 Y51 H52 Y55 G56 K59 W61 N62 W66 Y70 Y71,

Ca^2+^ binding site: D23 D27 W57 N58 W61 G64,

Na^+^ binding site: Y48 Y51,

K^+^ binding site: D23 D27,

PO^3-^_4_ binding site: N58 G64.

No binding site detected for the following ions:( Cu^2+^, Fe^2+^, Fe^3+^, Mg^2+^, Mn^2+^, CO^2-^_3_, NO^-^_2_, SO^2-^_4_).

**Mcfp3 v5**,

Zn^2+^ binding site: K3 Q21 D23 Y28 Y38 N39 Y43 Y45 Y48 Y51 H52 Y55 G56 W57 K59 W61 N62 W66 Y70 Y71,

Ca^2+^ binding site: G56 W57 N58 G60 W61 N62,

Na^+^ binding site: Y48 Y51.

No binding site detected for the following ions:( Cu^2+^, Fe^2+^, Fe^3+^, Mg^2+^, Mn^2+^, K^+^, CO^2-^_3_, NO^-^_2_, SO^2-^_4_, PO^3-^_4_).

**Mcfp3 v6**

Zn^2+^ binding site: K3 Q21 D23 Y28 Y44 G45 Y48 Y51 K52 Y54 R56 Y58 K62 W64 W68 W73, Ca^2+^ binding site: D29 G57 Y58 G59 N61,

Mg^2+^ binding site: G46 K47,

Na^+^ binding site: Y48,

CO^2-^_3_ binding site: L30 Y32,

SO^2-^_4_ binding site: Y28 N49 K52 G57 G71 R72,

No binding site detected for the following ions:( Cu^2+^, Fe^2+^, Fe^3+^, Mn^2+^, K^+^, NO^-^_2_, PO^3-^_4_).

**Mcfp3 v7**

Zn^2+^ binding site: K3 Q21 D23 Y26 Y28 D29 Y34 Y44 N45 Y48 Y51 Y54 Y57 H58 Y61 K65 W67 N68 N69 G70,

Ca^2+^ binding site: F4 S5 T7 D23 N40 P41 W42 G53 N55 G56 Y57 W63 N64 W67 ,

Mn^2+^ binding site: N69 G70,

Na^+^ binding site: Q21 Y54 Y57 N64,

CO^2-^_3_ binding site: Y32,

No binding site detected for the following ions:( Cu^2+^, Fe^2+^, Fe^3+^, Mg^2+^, K^+^, NO^-^_2_, SO^2-^_4_, PO^3-^_4_).

**Mcfp3 v8**

Zn^2+^ binding site: K3 Q21 S22 D23 Y26 Y28 Y32 Y38 N39 Y43 Y45 Y48 Y51 H52 Y55 W57 K59 W61 N62 W66,

Fe^3+^ binding site: S22 D23 Y26,

Mn^2+^ binding site: L17 A19 V20 S22 D23 A24 Y26 Y28,

Na^+^ binding site: Y42 Y48 Y51,

K^+^ binding site: I15 L17 F18 A19 V20 S22 D23 A24 Y26 Y28 Y32,

No binding site detected for the following ions:( Cu^2+^, Fe^2+^, Ca^2+^, Mg^2+^, CO^2-^_3_, NO^-^_2_, SO^2-^_4_, PO^3-^_4_).

**Mcfp3 v9,**

Zn^2+^ binding site: K3 Q21 D23 Y26 Y28 Y38 N39 Y42 Y43 Y45 N46 Y48 Y51 H52 Y55 W57 K59 W61 N62 W66,

Mg^2+^ binding site: D23 H52,

Mn^2+^ binding site: L9 L12 V13,

Na^+^ binding site: Y48 Y51,

SO^2-^_4_ binding site: L12 V13 G16 Y38,

PO^3-^_4_ binding site: F18 A19 V20 Y42,

No binding site detected for the following ions:( Cu^2+^, Fe^2+^, Fe^3+^, Ca^2+^, K^+^, CO^2-^_3_, NO^-^_2_).

**Mcfp3 v10**

Zn^2+^ binding site: K3 Q21 D23 Y26 Y28 Y38 N39 Y43 Y45 Y48 Y51 H52 Y55 W57 K59 W61 N62 W66,

Ca^2+^ binding site: V13 I15 N39 G44 N46 Y55 W57,

Mg^2+^ binding site: D23 H52,

Na^+^ binding site: Y42 Y48 Y51,

No binding site detected for the following ions:( :(Cu^2+^, Fe^2+^, Fe^3+^, Mn^2+^, K^+^, CO^2-^_3_, NO^-^_2_, SO^2-^_4_, PO^3-^_4_).

**Mcfp3 v11**

Zn^2+^ binding site: K3 Q21 D23 Y28 Y38 N39 Y43 Y45 Y48 Y51 H52 Y55 G56 W57 K59 W61 N62 W66,

Fe^3+^ binding site: D23 G27,

Ca^2+^ binding site: D23 G27,

Mg^2+^ binding site: D23 H52,

Mn^2+^ binding site: D23 G27,

Na^+^ binding site: Y42 Y43 Y45 Y48 Y51,

No binding site detected for the following ions:( Cu^2+^, Fe^2+^, K^+^, CO^2-^_3_, NO^-^_2_, SO^2-^_4_, PO^3-^_4_).

**Mcfp5**

Zn^2+^ binding site: K2 C5 C18 D20 S23 D26 Y28 D30 Y32 Y33 N39 Y40 P41 G43 H45 G46 Y47 H48 G49 H50 Y52 K53 Y57 K59 H83 Y87 Y90 Y91,

Mg^2+^ binding site: K85 G86,

Na^+^ binding site: N39 S44 K53,

CO^2-^_3_ binding site: H48 G51 K53 Y56,

No binding site detected for the following ions:( Cu^2+^, Fe^2+^, Fe^3+^, Ca^2+^, Mn^2+^, K^+^, NO^-^_2_, SO^2-^_4_, PO^3-^_4_).

**Mcfp6 v1**,

Zn^2+^ binding site: C17 E21 N26 R28 Y30 C31 N33 K34 G35 C36 Y40 R46 C49 Y51 K57 D59 C60 N62 Y63 C66 C67 Y73 K77 Y78 C80 T81 K83 C86 D88 D89 Y106 C109 Y112 N113 C115 C116 Y121,

Fe^2+^ binding site: S32 Y79 N113,

Na^+^ binding site: K34 C36 R37 G39 Y40 A64 C67 R75 P87 D88 F107 N108 C109 S111 Y112 N113 C115 C116.

SO^2-^_4_ binding site: S22 N45 C49 Y51 G52 S53.

No binding site detected for the following ions:( Cu^2+^, Fe^2+^, Ca^2+^, Mg^2+^, Mn^2+^, K^+^, CO^2-^_3_, NO^-^_2_, PO^3-^_4_).

**Mcfp6 v2**

Zn^2+^ binding site: C17 E21 N26 R28 Y30 C31 N33 K34 G35 C36 Y40 R46 C49 Y51 K57 D59 C60 R62 Y63 C66 C67 Y73 K77 C80 K83 C86 D89 F106 D108 C109 Y112 N113 C115 C116 Y121,

Na^+^ binding site: C36 R37 G39 Y40 A64 C67 N75 P87 Y107 D108 C109 S111 Y112 N113.

SO^2-^_4_ binding site: F11 I13 T14 C17 G18 I19.

No binding site detected for the following ions:( Cu^2+^, Fe^2+^, Fe^3+^, Ca^2+^, Mg^2+^, Mn^2+^, K^+^, CO^2-^_3_, NO^-^_2_, PO^3-^_4_).

**Mcfp6 v3,**

Zn^2+^ binding site: C17 E21 N26 R28 Y30 C31 N33 K34 G35 C36 Y40 R46 C49 Y51 K57 D59 C60 Y63 C66 C67 Y73 K77 C80 K83 C86 D89 F106 D10 8 C109 Y112 N113 C115 C116 Y121, Ca^2+^ binding site: Y79 Y99,

Mg^2+^ binding site: N71 S75 T81,

Na^+^ binding site: K34 C36 R37 G39 Y40 A64 C67 S75 P87 F90 Y107 D108 C109 S111 Y112 N113 C115 C116.

No binding site detected for the following ions:( Cu^2+^, Fe^2+^, Fe^3+^, Mn^2+^, K^+^, CO^2-^_3_, NO^-^_2_, SO^2-^_4_, PO^3-^_4_).

**Mcfp7 v1**

Zn^2+^ binding site: Y28 R29 R30 Y32 K33 G34 S35 H36 S37 G39 G40 H41 H44 G45 H49 Y51 Y55,

Ca^2+^ binding site: G34 S35 Y57 K58,

Na^+^ binding site: S38 S42 Y51.

No binding site detected for the following ions:( Cu^2+^, Fe^2+^, Fe^3+^, Mg^2+^, Mn^2+^, K^+^, CO^2-^_3_, NO^-^_2_, SO^2-^_4_, PO^3-^_4_).

**Mcfp7 v2**,

Zn^2+^ binding site: K27 Y28 Y32 Y35 K36 G39 S40 H41 S42 G44 G45 H46 S47 G49 G50 H51 H54 G55 G56 K57 Y61,

Mg^2+^ binding site: S43 G44,

Na^+^ binding site: H51,

CO^2-^_3_ binding site: S42 G56.

No binding site detected for the following ions:( Cu^2+^, Fe^2+^, Fe^3+^, Ca^2+^, Mn^2+^, K^+^, NO^-^_2_, SO^2-^_4_, PO^3-^_4_).

**Mcfp8**

Zn^2+^ binding site: P21 Y25 Y28 K36 Y37 K39 Y41 Y44 Y48 R51 Y52 H53 G55 K56 Y57 K60 Y61 K64,

Ca^2+^ binding site: G46 K47 G66,

Mg^2+^ binding site: V22 Y25,

Na^+^ binding site: Y41,

No binding site detected for the following ions:( Cu^2+^, Fe^2+^, Fe^3+^, Mn^2+^, K^+^, CO^2-^_3_, NO^-^_2_, SO^2-^_4_, PO^3-^_4_).

**Mcfp9 v1**

Zn^2+^ binding site: E18 Y20 Y23 N24 Y26 D27 D29 Y31 K34 H36 H43 H46 H47 G48 Y53 H54 H60 H61 H67 H68 W76 H84 H85 H86 H87 H88 H89 G90 A91 H93 Y94 G95 H97 H98 H99 G100 G101 H102 H103 H104 H105 R106 R107 H108 R109 K110 H111 K112 G113 S114 S118 K120 Y122 Y124,

Mg^2+^ binding site: G35 H36 H108 H111,

Na^+^ binding site: D27 G32 K34 Y53 H54 V57 H60 V64 G65 H67 W76 G78 P79 A91,

K^+^ binding site: F10 G21 D29 G35 H36 V37 L38 I41 I70,

No binding site detected for the following ions:( Cu^2+^, Fe^2+^, Fe^3+^, Ca^2+^, Mn^2+^, CO^2-^_3_, NO^-^_2_, SO^2-^_4_, PO^3-^_4_).

**Mcfp9 v2**

Zn^2+^ binding site: Y20 Y23 N24 Y26 D27 D29 Y31 K34 H36 H43 G44 V45 H46 H47 V49 H53 H54 G58 H60 H61 S73 H77 H78 H79 H80 H81 H82 T84 V85 H86 Y87 G89 H90 H91 H92 G93 G94 H95 H96 Y97 H98 N99 H100 H101 H102 R104 H105 R106 K107 H108 K109 S111 S113 S115 K117 Y119 Y121,

Ca^2+^ binding site: V45 H47,

Na^+^ binding site: Y26 D27 G32 K34 G35 H36 L38 H54 G71 P72 S73 G93,

K^+^ binding site: Y23 G25 V37 L38 I41 V45 V62 I63.

PO^3-^_4_ binding site: H78 H80 V85,

No binding site detected for the following ions:( Cu^2+^, Fe^2+^, Fe^3+^, Mg^2+^, Mn^2+^, CO^2-^_3_, NO^-^_2_, SO^2-^_4_).

**Mcfp10,**

Zn^2+^ binding site: R3 C6 D18 H30 D35 E36 Y37 D49 H55 C75 C87 H92 R94 C97 Y130 D132 D133 D134 D135 Y138 H152 D158 N159 C172 N179 W181 C184 N187 Y188 H189 R191 C194 D210 D212 D214 D221 Y222 D223 C236 H240 D246 N247 C260 C272 Y276 H277 R279 C282 D303.

Mg^2+^ binding site: V45 S46 T169 D210 D220 D221 Y299 D300.

Na^+^ binding site: S21 S23 E36 G38 G40 I41 P42 S43 S65 F66 G68 I116 D117 D132 R160 S162 Y163 G164 G165 D210 D212 Y213 D214 R248 S250 Y251 G252 G253 D300 Y301 N302 D303 S305.

No binding site detected for the following ions:( Cu^2+^, Fe^2+^, Fe^3+^, Ca^2+^, Mn^2+^, K^+^, CO^2-^_3_, NO^-^_2_, SO^2-^_4_, PO^3-^_4_).

**Mcfp11**

Zn^2+^ binding site: Y3 C6 G20 Y21 Y24 H27 H28 H29 H31 H38 H41 H44 Y45 G46 H47 H48 Y52 H57 H63 C77 W86 C89 R92 H94 R96 R98 C99 R102 H107 K113 Y115 H117 S119 G121 H122 Y123 K125 H127 H129 Y131 H133 H135 Y137 H139 H141 H143 Y145 H147 G148 H149 H151 H153 H155 Y157 H159 H161 H163 Y165 H167 H169 H171 G172 H173 H175 F177 S178 H179 H181 H183 A185 Y187 Y191 K193 H195 R198 I199 H200 H201 H202 R203 S205 A210 C215 H216 C227 H230 H232 R234 Y236 H240 K243 H245 S249 H251 Y255 H257 H259 H260 H262 H263 H264 H266 H268 W270 H279 H285 H286 H291 H297 H308 H319 E326 H330 H341 H348 H351 H352 H360 H374 H380 H382 H385 H388 H393 E395 Y398 H402 H404 H412 H416 E422 H423 Y427 C436 E437 D447 C448 H453 R455 C458 H466 D470 D491.

Cu^2+^ binding site: H127 H129 H139 H143 H149 H159 H169 H173 H179.

Na^+^ binding site: H44 T62 G72 Y115 S119 Y123 H127 H129 Y131 H133 H135 Y137 S138 H139 H143 H147 H149 H151 Y157 H159 H169 H171 H175 H179 H183 R203 F247 A254 Y255 G256 G399 Y400 H402 G403 H404 Y415 H416 P419 Y427 D477 P481.

No binding site detected for the following ions:( Fe^2+^, Fe^3+^, Ca^2+^, Mg^2+^, Mn^2+^, K^+^, CO^2-^_3_, NO^-^_2_, SO^2-^_4_, PO^3-^_4_).

**Mcfp12,**

Zn^2+^ binding site: C3 G19 Y20 D24 Y26 D28 Y53 H59 H62 H63 H64 G65 H66 H67 A68 Y70 H73 K74 H79 H81 R113 C115 C127 D130 Y131 D133 D136 C137 H151 H154 Y156 H158 C182 C194 H199 H200 C204 H212 H218 Y220 H221 H223 H225 H239 C253 C265 H270 R274 C275 H283 Y291 S292 Y314 H318 H319 N325 C339 E350 C351 C361 H369 E377 D378 H381 D385 D390 Y391 D398 H399 Y401 Y402 H406 H412 H413 C426 C438 H440 Y442 H443 S444 T447 C448 R451 H456 D464 H465 H472 H476 H483 H488 H494 H500 V501 H505 H516 H527 H545 H549 H555 K558 H560 E563 K570 N585 H589 Y591 Y593 H595 H597 H599 H601 H603 H605 H607 H609 Y617 H621 D626 H627 H628 Q638 C641 D643 Y647 W649 D651 C652 H655 H656 H657 S658 R659 C662 H670 D673 Y679 D680 E682 D684.

Cu^2+^ binding site: H59 H81 H93.

Ca^2+^ binding site: N196 R409 D464 H465 L473 H494 I498 K532.

Mg^2+^ binding site: D28 D32 S305 I479.

Na^+^ binding site: Y26 H59 H63 H64 H66 H67 G69 Y70 G72 H73 G161 L162 G163 Y220 H221 H223 S293 G294 G303 N306 T309 D311 Y315 K316 H318 G320 D378 D385 D390 F397 H405 G407 R409 Y593 G594 H595 G596 H597 G598 H599 G600 H601 G602 H603 G604 H605 G606 H607 G608 D626 H627 R629 G633 G634 D643.

SO^2-^_4_ binding site: S34 V48 R91.

No binding site detected for the following ions:( Fe^2+^, Fe^3+^, Mn^2+^, K^+^, CO^2-^_3_, NO^-^_2_, PO^3-^_4_).

**Mcfp13**

Zn^2+^ binding site: E2 E5 D9 C14 R18 E20 R25 H28 C30 Y50 Y52 Y55 Y65 N69 Y70 C99 W108 C111 Y112 K114 H116 R118 C121.

Ca^2+^ binding site: V42 V43 G109 S110 C111 Y112 R118 S119 R120 C121 F122 R123 R124 Y149 A152 T154 Y155 Y156.

Mn^2+^ binding site: L16 I21 N22 G24 R25 R85.

Na^2+^ binding site: D9 G71 G94.

No binding site detected for the following ions:( Cu^2+^, Fe^2+^, Fe^3+^, Mg^2+^, K^+^, CO^2-^_3_, NO^-^_2_, SO^2-^_4_, PO^3-^_4_).

**Mcfp14**

Zn^2+^ binding site: C11 H19 E22 C23 Q24 N25 G27 C30 N31 D32 F33 D35 C36 R39 Y41 C42 C43 K46 R48 R50 D58 C63 H64 N65 I66 G67 T68 E69 C73 N76 C90 C92 Q93 P95 C98 S101 D105 E110 T111 C114 Y116.

Ca^2+^ binding site: N25 D32 D35 R39 K46 E69 A71 L96 G100 S101.

Na^+^ binding site: D32 D35 G37 Y38 R39 G61 C63 W82 N83 G100.

No binding site detected for the following ions:( Cu^2+^, Fe^2+^, Fe^3+^, Mg^2+^, Mn^2+^, K^+^, CO^2-^_3_, NO^-^_2_, SO^2-^_4_, PO^3-^_4_).

**Mcfp15**

Zn^2+^ binding site: R17 N19 D26 E29 H31 D33 P35 Y37 H39 G41 Y42 N43 Y46 D58 H60 N62 H64 S66 G67 N68 G69 H70 Y73 G74 Q76 H89 H93 C96 C100 H115 R118 C121 Y134 N136 C139 H140 S142 N143 Y146 C152 D155 R164 C167 D168 R170 Q172 C173 D178 H189 H190 C192 H195 E204 E206 C207 C214 Q215 F217 C218 C220 H222 A223 S224 R226 Y231 K233 Y238 C239 K242 H248.

Mg^2+^ binding site: S71 K166 K176 K180.

Na^+^ binding site: H39 G40 Y42 N43 L48 F55 P56 Y57 D58 H60 H70 H140 Y146.

K^+^ binding site: G15 S16.

No binding site detected for the following ions:( Cu^2+^, Fe^2+^, Fe^3+^, Ca^2+^, Mn^2+^, CO^2-^_3_, NO^-^_2_, SO^2-^_4_, PO^3-^_4_).

**Mcfp16**

Zn^2+^ binding site: V13 E21 Q22 E24 C25 K27 H29 C30 E31 R33 R35 K37 N39 C40 D41 C42 G43 C44 H45 N46 C47 C48 K51 Y52 K53 C54 D58 C59 S64 R66 K68 C69 C71 N73 C74 E77 D86 D88 D90,

Ca^2+^ binding site: V11 V13 E21 E31 G32.

Mg^2+^ binding site: V49 R50 S64.

Mn^2+^ binding site: K27 H29.

Na^+^ binding site: D41 C42 C44 H45 N46 C48 D58.

No binding site detected for the following ions:( Cu^2+^, Fe^2+^, Fe^3+^, K^+^, CO^2-^_3_, NO^-^_2_, SO^2-^_4_, PO^3-^_4_).

**Mcfp17**

Zn^2+^ binding site: C9 C20 S22 N23 K24 C25 D30 Y38 D62 R69 C74 N78 H86 D87 D88 Y89 D92 Y94 K99 C108 D109 N111 K118 C125 N133 C136 Y138 K140 C141 N142 C146 N147 C160 D163 D169 C174 N177 W188 F189 P190 C192 C193 G194 C195 K196 K197 Y198 C202.

Fe^3+^ binding site: I61 D62 V63 G65 M66 E96 P97 Q98 W102,

Ca^2+^ binding site: R58 S60 D62 R69 K71 K72 S106.

Mg^2+^ binding site: D62.

Na^+^ binding site: G100 S114 P152 G194 C195.

K^+^ binding site: C20.

SO^2-^_4_ binding site: I61 M66 L67 P93.

PO^3-^_4_ binding site: T64 G65 M66 L67.

No binding site detected for the following ions:( Cu^2+^, Fe^2+^, Mn^2+^, CO^2-^_3_, NO^-^_2_)

***Mytilus galloprovincialis***

**Mgfp1**

Zn^2+^ binding site: K5 C9 C12 D17 H30 G31 K54 H55 Y66 H67 Y76 Y429 K430 K432 K450 Y509 Y529 T658 K724 Q750 Y751.

Na^+^ binding site: S22.

No binding site detected for the following ions:( Cu^2+^, Fe^2+^, Fe^3+^, Ca^2+^, Mg^2+^, Mn^2+^, K^+^, CO^2-^_3_, NO^-^_2_, SO^2-^_4_, PO^3-^_4_).

**Mgfp3 v1**

Zn^2+^ binding site: Q21 D23 D26 Y28 Y32 Y38 N42 Y43 Y50 K54 W60 W65,

Ca^2+^ binding site: M1 N2 N3 I4 A25 D26 Y28 Y38 G40 G41 N42 N44 R49 Y50 G51 G52 Y53 W56 N57 W60.

Na^+^ binding site: Y38 Y50.

No binding site detected for the following ions:( Cu^2+^, Fe^2+^, Fe^3+^, Mg^2+^, Mn^2+^, K^+^, CO^2-^_3_, NO^-^_2_, SO^2-^_4_, PO^3-^_4_).

**Mgfp3 v2**

Zn^2+^ binding site: Q21 D23 D26 Y28 Y32 W38 G39 Y41 Y44 N45 R51 Y52 Y55 K59 W61 W65 R66 W70.

Ca^2+^ binding site: S25 D26 G54 Y55 G56 G57 Y58.

Mg^2+^ binding site: N31 G33 G42 R46,

Na^+^ binding site: W38 W61.

K^+^ binding site: D23 S25.

No binding site detected for the following ions:( Cu^2+^, Fe^2+^, Fe^3+^, Mn^2+^, CO^2-^_3_, NO^-^_2_, SO^2-^_4_, PO^3-^_4_).

***Mytilus edulis***

**Mefp1**

Zn^2+^ binding site: C9 C12 T15 D17 H30 Y34 Y94 Y163 Y193 P217 Y275 P279 Y311 P351 Y357. Fe^3+^ binding site: K324 S494.

Mg^2+^ binding site: G3.

No binding site detected for the following ions:( Cu^2+^, Fe^2+^, Ca^2+^, Mn^2+^, Na^+^, K^+^, CO^2-^_3_, NO^-^_2_, SO^2-^_4_, PO^3-^_4_).

**Mefp2**

Zn^2+^ binding site: C11 Q13 L14 C15 L16 G17 T18 P20 P22 Y23 D24 D25 D26 E27 D28 D29 T31 P32 V34 S40 R41 Y42 N46 P47 C48 K51 C53 K54 Y55 N56 C59 G64 S65 K67 C68 C70 G73 Y74 C79 K82 N83 A84 C85 N88 C90 K91 N92 K93 R95 C96 K101 T102 K104 C105 C107 G110 N111 L115 C116 E117 R118 V120 C121 N124 P125 C126 K127 N128 K129 C132 L135 K141 C142 C144 G147 Y148 G150 R152 C153 E154 H156 A157 C158 N161 P162 C163 K164 N165 N166 G167 C169 D172 K178 C179 C181 G184 Y185 C190 E192 N193 A194 C195 N198 P199 C200 R201 N202 G203 G204 C206 D209 D213 T215 C216 D217 C218 G221 Y222 C227 K229 Y230 V231 C232 N235 C237 K238 N239 S240 G241 C243 S244 D246 R252 C253 C255 G258 Y259 T263 C264 K265 N267 V268 C269 N272 R274 K275 N276 S277 C280 N282 K283 S285 S286 C289 C291 G294 Y295 G297 T299 C300 N303 A304 C305 N308 C310 N312 S313 C316 N318 S322 N324 C325 C327 G330 Y331 K335 C336 E338 H339 V340 C341 N344 C346 Q347 N348 R349 G350 C352 E355 D358 R361 C362 C364 G367 Y368 G370 T372 C373 D374 E375 N376 V377 C378 N381 C383 Q384 N385 K386 C389 P391 D392 D396 K399 C400 C402 G405 C411 E412 D413 K414 P415 N416 P417 C418 K421 C423 N425 G426 C429 Y431 K434 T437 C438 C440 G443 Y444 H448 C449 K452 A453 Y454 P456 N457 P458 C459 R462 C464 K465 N466 R467 C470 T475 C479 C481 G484 Y485 C490 A491 S494 P496 D500 E503.

Ca^2+^ binding site: R95 C96 C169 K178 N193 C195 N202 G204 K205 C206 S207 D209 F211 D213 C216 C218 G221 F223 S260 P262 N267 C269 N272 P273 C280 N282 C289 C291 G294 Y295 S296 G301 N303 C310 N312 C316 N318 C325 C327 G330 Y331 S332 C352 E355 C362 C364 G367 Y368 S369 T372 D374 N376 D396 F398 K399 C400 K407 P409 N416 C418 K421 K424 N425.

Na^2+^ binding site: T10 C11 T12 Q13 C15 L16 G17 T18 S19 R21 D24 D25 D26 E27 D28 D29 Y30 T31 P32 P33 V34 K36 R41 P44 V45 N46 P47 C48 K51 P52 C53 K54 Y55 N56 G57 N83 N88 Q89 C90 K91 N92 K93 S94 N124 P125 C126 K127 N128 K129 G130 E154 K159 N161 P162 C163 K164 N165 N166 G167 Q191 N193 C195 K196 N198 P199 C200 R201 N202 G203 G204 C232 N235 P236 C237 K238 N239 S240 G241 C243 S248 K265 N267 C269 N272 P273 R274 K275 N276 S277 G278 G301 N303 C305 K306 N308 P309 C310 K311 N312 S313 G314 C341 N344 P345 Q347 N348 R349 R357 D374 C378 N381 P382 C383 Q384 N385 K386 D395 N416 C423 K424 N425 T450 N457 P458 A460 R462 P463 C464 K465 N466 R467 G468 P495 Y498.

SO^2-^_4_, binding site: S296 V302 C305 Y406 G408.

No binding site detected for the following ions:( Cu^2+^, Fe^2+^, Fe^3+^, Mg^2+^, Mn^2+^, K^+^, CO^2-^_3_, NO^-^_2_, PO^3-^_4_).

***Mytilus unguiculatus***

**Mufp2**,

Zn^2+^ binding site: C11 Q13 C15 L16 G17 T18 T21 T22 Q23 Y24 D25 D26 D27 E28 D29 D30 Y31 K32 P33 T35 A36 K38 P42 K43 Y44 N48 P49 C50 K53 C55 K56 Y57 N58 Q60 C61 K69 C70 C72 G75 Y76 C81 N82 N85 A86 C87 N90 P91 C92 K93 N94 K95 C98 K103 K106 C107 C109 G112 N113 C118 E119 K120 N121 V122 C123 N126 C128 K129 N130 R131 C134 W137 K143 C144 C146 G149 Y150 R154 C155 E156 H158 V159 C160 P162 N163 C165 K166 N167 K168 C171 F172 P173 D174 K180 C181 C183 G186 Y187 C192 E193 K195 P196 N197 P198 C199 K202 C204 K205 N206 G207 C210 Y212 T218 C219 C221 G224 Y225 H229 C230 D232 A234 K236 P237 N238 P239 C240 R243 C245 K246 N247 G248 C251 N256 C260 C262 G265 Y266 C271 A272 S275 S278 E282 E283.

Fe^3+^ binding site: P89 N94.

Ca^2+^ binding site: T12 K56 Y150 E156 V157 H158 V159 P173 D174 G178 K195 P196 P198 S201 P203 Y212 N213 Y217 K236 P237 P239 V253 K254 G255 G257 K269.

Na^+^ binding site: F3 C15 L16 G17 T18 A19 P20 E28 P33 D34 P41 K43 N48 P54 C55 K56 Y57 N58 G59 N85 C92 K93 N94 K95 S96 K129 N130 R131 G132 H158 N163 C165 K166 N167 K205 N206 G207 G208 N238 P244 C245 K246 N247 G248 G249 P276.

CO^2-^_3_ binding site: N130 R131.

No binding site detected for the following ions:( Cu^2+^, Fe^2+^, Mg^2+^, Mn^2+^, K^+^, NO^-^_2_, SO^2-^_4_, PO^3-^_4_).

**Mufp3**

Zn^2+^ binding site: K3 Q21 Y27 D28 Y37 N38 Y41 Y42 Y44 N45 Y47 Y50 H51 Y54 W56 K58 W60 N61 W65 Y70.

Na^2+^ binding site: Y47 Y50.

No binding site detected for the following ions:( Cu^2+^, Fe^2+^, Fe^3+^, Ca^2+^, Mg^2+^, Mn^2+^, K^+^, CO^2-^_3_, NO^-^_2_, SO^2-^_4_, PO^3-^_4_).

**Mufp3 v1**

Zn^2+^ binding site: N3 Q21 D23 Y26 Y28 Y32 Y38 N39 Y42 Y43 Y45 N46 Y48 Y51 H52 Y55 W57 K59 W61 N62 W66 Y70 Y71.

Ca^+^ binding site: Y29 N39 Y42 Y43 Y45 N46 G47 G50 W57 W61.

Na^2+^ binding site: Y48 Y51.

No binding site detected for the following ions:( Cu^2+^, Fe^2+^, Fe^3+^, Mg^2+^, Mn^2+^, K^+^, CO^2-^_3_, NO^-^_2_, SO^2-^_4_, PO^3-^_4_).

**Mufp3 v2**

Zn^2+^ binding site: N3 Q21 D23 Y26 Y28 Y32 Y38 N39 Y45 Y48 Y51 H52 Y55 G56 W57 K59 W61 N62 W66 Y71.

Ca^2+^ binding site: N39 Y42 Y43 Y45 N46 G47 G50 G56 W57 N58 G60 W61 N62.

Na^+^ binding site: Y48 Y51.

No binding site detected for the following ions:( Cu^2+^, Fe^2+^, Fe^3+^, Mg^2+^, Mn^2+^, K^+^, CO^2-^_3_, NO^-^_2_, SO^2-^_4_, PO^2-^_4_).

**Mufp3 v3**

Zn^2+^ binding site: N3 Q21 D23 Y28 N32 Y38 K39 R47 Y50 W52 W56 W61.

Mg^2+^ binding site: G62 R63 K64.

Mn^2+^ binding site: A11 L14 I15.

CO^2-^_3_ binding site: I6 L10.

No binding site detected for the following ions:( Cu^2+^, Fe^2+^, Fe^3+^, Ca^2+^, Na^+^, K^+^, NO^-^_2_, SO^2-^_4_, PO^3-^_4_).

**Mufp3 v4**

Zn^2+^ binding site: N3 Q21 D23 Y26 Y28 Y32 Y38 N39 Y42 Y45 N46 G47 Y48 N49 Y51 H52 Y55 G56 W57 K59 W61 N62 W66 Y70 Y71.

Ca^2+^ binding site: G56 W57 N58 G60 W61 N62 Y70 L77.

Mn^2+^ binding site: L9 L12 V13.

Na^2+^ binding site: Y42 Y48 Y51.

No binding site detected for the following ions:( Cu^2+^, Fe^2+^, Fe^3+^, Mg^2+^, K^+^, CO^2-^_3_, NO^-^_2_, SO^2-^_4_, PO^3-^_4_).

**Mufp3 v5**

Zn^2+^ binding site: N3 Q21 D23 Y26 Y28 Y32 Y38 N39 Y42 Y43 C45 N46 G47 Y48 N49 Y51 H52 Y55 G56 W57 K59 W61 N62 W66 Y70 Y71.

Ca^2+^ binding site: G56 W57 N58 G60 W61 N62 Y70.

Na^2+^ binding site: Y42 Y48 Y51.

No binding site detected for the following ions:( Cu^2+^, Fe^2+^, Fe^3+^, Mg^2+^, Mn^2+^, K^+^, CO^2-^_3_, NO^-^_2_, SO^2-^_4_, PO^3-^_4_).

**Mufp3 v6**

Zn^2+^ binding site: N3 S5 Q21 D23 Y26 D27 Y28 Y38 N39 Y42 S45 Y47 N48 Y50 H51 G52 Y54 G55 K58 W60 N61 W65 Y69 Y70.

Ca^2+^ binding site: G40 G43 Y44 S45 G46 G49.

Na^2+^ binding site: Y38.

No binding site detected for the following ions:( Cu^2+^, Fe^2+^, Fe^3+^, Mg^2+^, Mn^2+^, K^+^, CO^2-^_3_, NO^-^_2_, SO^2-^_4_, PO^3-^_4_).

**Mufp3 v7**

Zn^2+^ binding site: N3 Q21 E23 Y26 Y28 Y38 N39 Y42 Y45 N46 G47 Y48 Y51 H52 Y55 G56 W57 K59 W61 N62 W66 Y70 Y71,

Ca^2+^ binding site: W57 W61 Y70 L77.

Na^+^ binding site: Y42 Y48 Y51.

CO^2-^_3_ binding site: R54 G56.

SO^2-^_4_ binding site: N46 K59 G68.

No binding site detected for the following ions:( Cu^2+^, Fe^2+^, Fe^3+^, Mg^2+^, Mn^2+^, K^+^, NO^-^_2_, PO^3-^_4_).

**Mufp3 v8**

Zn^2+^ binding site: N3 Q21 E23 Y26 Y28 Y32 Y38 N39 Y45 Y48 Y51 H52 Y55 G56 K59 W61 K62 W66 F71.

Mg^2+^ binding site: Y51 K62.

Na^+^ binding site: Y42 Y48 Y51.

No binding site detected for the following ions:( Cu^2+^, Fe^2+^, Fe^3+^, Ca^2+^, Mn^2+^, K^+^, CO^2-^_3_, NO^-^_2_, SO^2-^_4_, PO^3-^_4_).

**Mufp3 v9**

Zn^2+^ binding site: N3 Q21 E23 Y26 Y28 Y38 N39 Y42 Y45 Y48 Y51 H52 Y55 G56 W57 K59 W61 N62 W66 Y71

Ca^2+^ binding site: W57 N58 W61

Mn^2+^ binding site: I6 L9 L10.

Na^+^ binding site: Y48 Y51.

K^+^ binding site: L12 L14 N39 N46 W57 K59.

No binding site detected for the following ions:( Cu^2+^, Fe^2+^, Fe^3+^, Mg^2+^, CO^2-^_3_, NO^-^_2_, SO^2-^_4_, PO^3-^_4_).

**Mufp3 v10**

Zn^2+^ binding site: N3 Q21 D23 F28 Y38 N39 Y42 Y43 Y51 P52 Y55 W57 K59 W61 K62 W66 Y71.

Na^2+^ binding site: Y48 Y51.

No binding site detected for the following ions:( Cu^2+^, Fe^2+^, Fe^3+^, Ca^2+^, Mg^2+^, Mn^2+^, K^+^, CO^2-^_3_, NO^-^_2_, SO^2-^_4_, PO^3-^_4_).

**Mufp3 v11**

Zn^2+^ binding site: N3 Q21 E23 Y26 Y28 Y38 N39 Y42 Y45 Y48 Y51 H52 Y55 G56 W57 K59 W61 N62 W66 Y71.

Ca^2+^ binding site: G56 N58 G60 N62 W66.

Na^2+^ binding site: Y48 Y51 G72 N73.

No binding site detected for the following ions:( Cu^2+^, Fe^2+^, Fe^3+^, Mg^2+^, Mn^2+^, K^+^, CO^2-^_3_, NO^-^_2_, SO^2-^_4_, PO^3-^_4_).

**Mufp3 v12**

Zn^2+^ binding site: N3 Q21 E23 Y26 Y28 Y38 N39 Y42 Y45 N46 Y48 Y51 H52 Y55 G56 K59 W61 N62 W66 G67 Y70 Y71,

Ca^2+^ binding site: L17 V20 A24.

Mg^2+^ binding site: Y48 Y51.

Na^+^ binding site: Y48 Y51.

K^+^ binding site: L17 V20 A24.

CO^2-^_3_ binding site: R54 G56.

No binding site detected for the following ions:( Cu^2+^, Fe^2+^, Fe^3+^, Mn^2+^, NO^-^_2_, SO^2-^_4_, PO^3-^_4_).

**Mufp3 v13**

Zn^2+^ binding site: N3 Q21 D23 Y26 F28 Y38 N39 F45 Y48 F51 P52 Y55 G56 K59 K62 W66.

Na^+^ binding site: Y48 F51 G72.

K^+^ binding site: L12 L14 G16 N39 N46 Y55 G57.

No binding site detected for the following ions:( Cu^2+^, Fe^2+^, Fe^3+^, Ca^2+^, Mg^2+^, Mn^2+^, CO^2-^_3_, NO^-^_2_, SO^2-^_4_, PO^3-^_4_).

**Mufp3 v14**

Zn^2+^ binding site: Q21 D23 Y28 Y32 Y38 G39 Y45 Y48 Y52 G53 K56 W58 W62 W67.

Ca^2+^ binding site: G25 G26 G51 Y52 G53 N55.

Na^+^ binding site: Y48.

K^+^ binding site: R50 K56 G57 W58 N63.

No binding site detected for the following ions:( Cu^2+^, Fe^2+^, Fe^3+^, Mg^2+^, Mn^2+^, CO^2-^_3_, NO^-^_2_, SO^2-^_4_, PO^3-^_4_).

**Mufp6**

Zn^2+^ binding site: C17 D25 K27 R29 Y31 C32 N34 K35 G36 C37 Y41 K47 C50 N60 C61 N62 S63 Y64 C67 C68 L69 Y74 K78 C81 K84 C87 D90 Y107 C110 Y113 N114 C116 C117 Y122.

Ca^2+^ binding site: Y99 G111.

Na^+^ binding site: C37 R38 G40 Y41 A65 C68 K76 P88 N89 Y91 F108 N109 C110 S112 Y113 N114.

No binding site detected for the following ions:( Cu^2+^, Fe^2+^, Fe^3+^, Mg^2+^, Mn^2+^, K^+^, CO^2-^_3_, NO^-^_2_, SO^2-^_4_, PO^3-^_4_).

**Mufp6 v1**

Zn^2+^ binding site: C17 K21 D26 D28 R30 Y32 C33 N35 K36 G37 C38 Y42 R48 C51 K59 D61 C62 N63 Y65 C68 C69 Y75 K79 C82 K85 C88 D91 Y108 C111 Y114 N115 C117 C118 Y123.

Na^+^ binding site: C38 R39 G41 Y42 A66 C69 S77 P89 K90 Y92 F109 N110 C111 S113 Y114 N115.

No binding site detected for the following ions:( Cu^2+^, Fe^2+^, Fe^3+^, Ca^2+^, Mg^2+^, Mn^2+^, K^+^, CO^2-^_3_, NO^-^_2_, SO^2-^_4_, PO^3-^_4_).

**Mufp6 v2**

Zn^2+^ binding site: C17 K21 D26 D28 R30 Y32 C33 N35 K36 G37 C38 Y42 R48 C51 K59 D61 C62 N63 Y65 A66 G67 C68 C69 L70 R72 Y75 K79 C82 K85 C88 D91 Y108 Y109 C111 Y114 N115 C117 C118 Y123.

Cu^2+^ binding site: V12 L16 Y81.

Na^2+^ binding site: C38 R39 G41 A66 C69 C88 P89 Y109 N110 C111 S113 Y114 N115.

No binding site detected for the following ions:( Fe^2+^, Fe^3+^, Ca^2+^, Mg^2+^, Mn^2+^, K^+^, CO^2-^_3_, NO^-^_2_, SO^2-^_4_, PO^3-^_4_).

**Mufp6 v3**

Zn^2+^ binding site: C17 K21 D26 D28 R30 Y32 C33 N35 K36 G37 C38 Y42 D46 R48 C51 K59 D61 C62 N63 Y65 C68 C69 Y75 K79 C82 K85 C88 D91 Y108 C111 Y114 N115 C117 C118 Y123.

Ca^2+^ binding site: D28.

Na^2+^ binding site: C38 R39 G41 A66 C69 Y75 T77 P89 N90 F109 N110 C111 S113 Y114 N115.

No binding site detected for the following ions:( Cu^2+^, Fe^2+^, Fe^3+^, Mg^2+^, Mn^2+^, K^+^, CO^2-^_3_, NO^-^_2_, SO^2-^_4_, PO^3-^_4_).

**Mufp6 v4**

Zn^2+^ binding site: C17 K21 D26 D28 R30 Y32 C33 N35 K36 G37 C38 Y42 R48 C51 K59 D61 C62 N63 Y65 C68 C69 N73 Y75 K79 C82 K85 C88 D91 Y108 C111 Y114 N115 C117 C118 Y123.

Na^+^ binding site: C38 R39 A66 C69 T77 P89 F109 N110 C111 S113 Y114 N115.

No binding site detected for the following ions:( Cu^2+^, Fe^2+^, Fe^3+^, Ca^2+^, Mg^2+^, Mn^2+^, K^+^, CO^2-^_3_, NO^-^_2_, SO^2-^_4_, PO^3-^_4_).

**Mufp6 v5**

Zn^2+^ binding site: C17 K21 D26 E28 R30 Y32 C33 N35 K36 G37 C38 Y42 D46 R48 C51 K59 D61 C62 N63 Y65 C68 C69 N73 Y75 K79 C82 K85 C88 D91 Y108 C111 Y114 N115 C117 C118 Y123.

Na^+^ binding site: C38 R39 G41 A66 C69 T77 P89 F109 N110 C111 S113 Y114 N115.

No binding site detected for the following ions:( Cu^2+^, Fe^2+^, Fe^3+^, Ca^2+^, Mg^2+^, Mn^2+^, K^+^, CO^2-^_3_, NO^-^_2_, SO^2-^_4_, PO^3-^_4_).

**Mufp6 v6**

Zn^2+^ binding site: C17 K21 D26 D28 R30 Y32 C33 N35 K36 G37 C38 Y42 D46 R48 C51 K59 D61 C62 N63 S64 Y65 G67 C68 C69 N73 Y75 K79 C82 K85 C88 D91 Y108 C111 Y114 N115 C117 C118 R120 Y123.

Na^+^ binding site: C38 R39 G41 A66 C69 T77 P89 F109 N110 C111 S113 Y114 N115.

No binding site detected for the following ions:( Cu^2+^, Fe^2+^, Fe^3+^, Ca^2+^, Mg^2+^, Mn^2+^, K^+^, CO^2-^_3_, NO^-^_2_, SO^2-^_4_, PO^3-^_4_).

**Mufp6 v7**

Zn^2+^ binding site: C17 K21 D26 D28 R30 Y32 C33 N35 K36 G37 C38 Y42 R48 Y50 C51 Y53 K59 D61 C62 N63 Y65 C68 C69 Y75 K79 C82 K85 Y86 G87 C88 D91.

Na^+^ binding site: C38 R39 G41 Y42 A66 C69 S77 P89 K90 Y92.

K^+^ binding site: V12 L16 I19.

No binding site detected for the following ions:( Cu^2+^, Fe^2+^, Fe^e+^, Ca^2+^, Mg^2+^, Mn^2+^, CO^2-^_3_, NO^-^_2_, SO^2-^_4_, PO^3-^_4_).

**Mufp6 v8**

Zn^2+^ binding site: C12 K16 D21 D23 R25 Y27 C28 N30 K31 G32 C33 Y37 D41 R43 Y45 C46 Y48 K54 D56 C57 N58 Y60 G62 C63 C64 P66 Y70 K74 C77 K80 C83 K85 D86.

Mg^+^ binding site: N58 S59 P66 G82.

Na^+^ binding site: C33 R34 G36 A61 C64 T72 P84 K85 Y87.

No binding site detected for the following ions:( Cu^2+^, Fe^2+^, Fe^3+^, Ca^2+^, Mn^2+^, K^+^, CO^2-^_3_, NO^-^_2_, SO^2-^_4_, PO^3-^_4_).

**Mufp6 v9**

Zn^2+^ binding site: C6 D13 D15 D16 D18 Y21 K22 R24 Y26 C27 N29 K30 G31 C32 Y36 K42 Y44 C45 Y47 N55 C56 N57 Y59 A60 C62 C63 Y69 K73 C76 K79 Y80 C82.

Ca^2+^ binding site: D15 D18 T53 N55.

Na^+^ binding site: C32 R33 G35 Y36 A60 C63 K71 P83 Y86.

No binding site detected for the following ions:( Cu^2+^, Fe^2+^, Fe^3+^, Mg^2+^, Mn^2+^, K^+^, CO^2-^_3_, NO^-^_2_, SO^2-^_4_, PO^3-^_4_).

***Mizuhopecten yessoensis***

**Myfp1 v1**

Zn^2+^ binding site: M7 E9 D41 D61 D91 H134 D161 D171 D181 D211 D241 H264 D271 D281 D291 D301 D311 D325 D335 D345 D355 D365 D375 D395 D405 D415 D435 D445 D465 H468 D495 T500 H501 H502.

Ca^2+^ binding site: D121 T122 Q194 Q204 D241 E249 D251 N319 S362 T364 D365.

Na^+^ binding site: N195 T197 S198.

No binding site detected for the following ions:( Cu^2+^, Fe^2+^, Fe^3+^, Mg^2+^, Mn^2+^, K^+^, CO^2-^_3_, NO^-^_2_, SO^2-^_4_, PO^3-^_4_).

**Myfp1 v2**

Zn^2+^ binding site: D2 E6 D32 D52 D62 H84 D112 Q125 W181 S183 Q205 P207 T209 Q225 G234 Q235 R244 P247 Q255 P257 Q265 G274 Q285 P287 C291 G294 P297 G304 G314 Q315 P317.

Ca^2+^ binding site: F5 E17 Q215 S216 P217 G218.

Mn^2+^ binding site: T319 Y321.

Na^+^ binding site: P317.

K^+^ binding site: L233 Q235.

No binding site detected for the following ions:( Cu^2+^ Fe^2+^, Fe^3+^, Mg^2+^, CO^2-^_3_, NO^-^_2_, SO^2-^_4_, PO^3-^_4_).

***Perna canaliculus***

**Pcfp1 v1**

Zn^2+^ binding site: C4 C9 Y27 C33 K35 N36 C38 N41 C44 Y49 K52 C53 C55 Y57 G58 Y59 G61 C64 K67 Y73 Y77 Y81 Y89 K91 Y93 Y97 Y105 K107 Y113 Y117 Y121 K125 Y139 K141 Y149 Y157 K169 Y175 K191 K195 K203 Y215 K235 Y237 Y243 Y247 K253 K267 Y269 K271 K283 K285 K293 K297 K309 K327 K333 K337 K345 C347 K349 Y351 Y355 K357 Y359 K361 K367 K371 K391 C397 K399 C402 N405 C408 K416 C417 C419 N420 G422 Y423 R427 C428 H436.

Fe^3+^ binding site: K143 K323.

Ca^2+^ binding site: P236 Y237 P330 P350 Y351 H436.

Na^+^ binding site: F10 K289 Y317.

No binding site detected for the following ions:( Cu^2+^, Fe^2+^, Mg^2+^, Mn^2+^, K^+^, CO^2-^_3_, NO^-^_2_, SO^2-^_4_, PO^3-^_4_).

**Pcfp1 v2,**

Zn^2+^ binding site: C4 C9 Y27 C33 N36 C38 N41 C44 K47 Y49 K52 C53 C55 K56 Y57 Y59 G61 C64 K67 Y73 Y77 Y89 Y93 Y97 K99 Y113 Y117 K11 9 K121 K125 K129 K137 K143 Y145 Y153 K165 K169 Y171 K187 Y189 K199 Y211 K227 K235 Y239 Y261 Y265 K267 Y277 K285 K289 K293 K297 Y299 K305 K307 Y309 Y313 Y317 K319 K323 K341 C343 K345 Y347 K349 Y351 Y355 K357 K359 K363 K367 K375 K379 K383 Y385 K387 C393 K395 C398 N401 C404 S410 Y411 K412 C413 C415 N416 G418 Y419 G421 R423 C424 H432.

Ca^2+^ binding site: P338 P350 Y355 K367 P376 Y377 K379 H432.

Na^+^ binding site: Y303 P306.

K^+^ binding site: P174 K177 P178 V180 K181.

No binding site detected for the following ions:( Cu^2+^, Fe^2+^, Fe^3+^, Mg^2+^, Mn^2+^, CO^2-^_3_, NO^-^_2_, SO^2-^_4_, PO^3-^_4_).

**Pcfp1 v3**,

Zn^2+^ binding site: C4 C9 Q15 Y25 Y29 Y33 K35 Y41 K43 Y45 Y57 K59 Y65 K71 Y73 Y89 K97 Y99 Y121 Y133 K137 Y183 Y191 Y195 K203 K207 Y215 Y219 Y249 K257 Y259 K265 K269 Y271 Y275 K283 Y289 Y307 K309 Y311 C315 Y323 Y327 K329 K331 Y333 K335 Y337 Y349 K359 C365 K367 C370 N373 C376 Y381 S382 K384 C385 C387 N388 G390 Y391 R395 C396 D397 H404.

Fe^3+^ binding site: K153 Y179.

Ca^2+^ binding site: P228 Y229 P322 P326 Y327 V328.

Mg^2+^ binding site: K22 K23 P24.

No binding site detected for the following ions:( Cu^2+^, Fe^2+^, Mn^2+^, Na^+^, K^+^, CO^2-^_3_, NO^-^_2_, SO^2-^_4_, PO^3-^_4_).

**Pcfp1 v4**

Zn^2+^ binding site: C4 C9 Q15 K23 Y29 K31 K35 K39 K55 K59 K63 K67 K71 Y73 K77 K85 Y91 K99 Y101 Y109 K121 K125 K129 Y131 K137 Y141 Y145 Y149 K157 Y167 Y171 Y175 Y179 K183 K191 Y195 Y199 Y203 K205 K209 K219 Y221 K223 K227 K237 K241 K245 Y247 K249 K253 Y255 Y259 K261 K263 K267 Y273 K285 Y287 K289 Y295 K297 C299 K301 K305 Y307 K309 K313 K315 Y317 K319 K335 K343 C349 K351 C354 Y356 N357 C360 S366 K368 C369 C371 N372 Y375 C380 D381 H388.

Fe^3+^ binding site: Y217 K271.

Ca^2+^ binding site: P224 Y225 P302 Y303.

No binding site detected for the following ions:( Cu^2+^, Fe^2+^, Mg^2+^, Mn^2+^, Na^+^, K^+^, CO^2-^_3_, NO^2-^_2_, SO^2-^_4_, PO^3-^_4_).

***Perna viridis***

**Pvfp1 v1**

Zn^2+^ binding site: H23 T28 H42 H52 P264 P283 P304 S307 K344 T357 H362 T392 P393 K394 P404 A408 P425 T432 P433 W439 P443 T446 A449 W450 H451 H453 Y455 G457 Y458 G507 H513 D517 Y519 W543 A545 H547 H549 W551.

Fe^3+^ binding site: H547 H549.

Ca^2+^ binding site: D517 E528.

Mg^2+^ binding site: G525 K526 G548 G550 A559.

Na^+^ binding site: P443 G548 H549 W551.

No binding site detected for the following ions:( Cu^2+^, Fe^2+^, Mn^2+^, K^+^, CO^2-^_3_, NO^-^_2_, SO^2-^_4_, PO^3-^_4_).

**Pvfp1 v2**

Zn^2+^ binding site: H23 P25 K254 K260 T267 T302 W309 P313 P315 W317 T318 A319 W320 H321 H323 G324 G326 Y328 H383 Y389 W413 A415 H417 H419 W421 T422 W424.

Fe^3+^ binding site: H417 H419.

Ca^2+^ binding site: P123 K124.

Na^+^ binding site: M5 P33 G418 H419 W421.

No binding site detected for the following ions:( Cu^2+^, Fe^2+^, Mg^2+^, Mn^2+^, K^+^, CO^2-^_3_, NO^-^_2_, SO^2-^_4_, PO^3-^_4_).

**Pvfp5**

Zn^2+^ binding site: C12 C14 Y15 Q17 A18 Y19 D20 R22 D23 P24 C25 R28 C30 N32 G33 G34 C36 R38 S41 C45 C47 G50 Y51 G53 N55 C56 Y58 N59 C61 S64 P65 C66 K67 N68 G69 T71 C72 C74 K79 C82 C84 G87 C93 Q94 Y95 G96 P97 C98 N101 C103 N105 G106 C109 Y111 K119 C120 C122 G125 Y126 C131 Y136 Y137 K138 D139 C141 G142 C144 N146 G147 N149 C150 C152 N153 Y155 F159 C160 C162 G165 Y166 C171.

Ca^2+^ binding site: Y21 R22 P24 P27 P29 R38 K39 S41 S42 Y43 Q57 Y58 N59 S60 C74 L75 G77 Y95 P97 Y111 G114 L115 F117 K129 K134 R135 K138 C152 N153 G156 K157.

Na^+^ binding site: D23 V31 N32 G33 Q57 N59 K67 N68 G69 L104 N105 G106 Q132 D139 G142 L145 N146 I151 Y155 F159 G165 Y166.

CO^2-^_3_ binding site: C47 R48.

No binding site detected for the following ions:( Cu^2+^, Fe^2+^, Fe^3+^, Mg^2+^, Mn^2+^, K^+^, NO^-^_2_, SO^2-^_4_, PO^3-^_4_).

**Pvfp6**

Zn^2+^ binding site: C6 E24 Q28 C29 I35 C38 C40 I41 E43 N44 S45 E46 C47 D50 N52 C53 A56 C59 C60 D61 F62 C64 C66 N67 C70 C80 G84 Y87 F93 D97 C99 C102 C104 N105 D107 C112 K115 C117.

Ca^2+^ binding site: C102 C112.

Na^+^ binding site: Q16 D50 S51 C53 C60 Y87 V96 D97 C99 N100.

No binding site detected for the following ions:( Cu^2+^, Fe^2+^, Fe^3+^, Mg^2+^, Mn^2+^, K^+^, CO^2-^_3_, NO^-^_2_, SO^2-^_4_, PO^3-^_4_).
